# Durum Wheat cv. Svevo Reference Genome Rel.2.0: A Comprehensive Tool for Wheat Genomics

**DOI:** 10.1111/pbi.70673

**Published:** 2026-07-13

**Authors:** Elisabetta Mazzucotelli, Cristian Forestan, Gina Zastrow‐Hayes, David Swarbreck, Yael Lev‐Mirom, Emile Cavalet‐Giorsa, Matteo Bozzoli, Anna Maria Mastrangelo, Daniela Marone, Vincent Ranwez, Johanna Girodolle, Victor Llaca, Kevin Fengler, Charlotte Harris, Helena Toegelová, Pavla Navrátilová, Primetta Faccioli, Francesca Desiderio, Ana Paola Valladares, Gemy Kaithakottil, Rachel L. Rusholme‐Pilcher, Manuel Spannagl, Heidrun Gundlach, Klaus F. X. Mayer, Victoria C. Blake, Justin D. Faris, Steven S. Xu, Taner Z. Sen, Eric Yao, Julio Isidro y Sánchez, Chunyi Liu, Muhammad A. Farooq, Sandra Stefanelli, Chiara Cappucci, Leah Oren, Tamar Eilam, Mario Giorgioni, Alessandra Stella, Barbara Lazzari, Massimiliano Lauria, Aldo Ceriotti, Raul Pirona, Elahe Tavakol, Valentyna Klymiuk, Jennifer Ens, Harmeet Singh Chawla, Sean Walkowiak, Nicola Pecchioni, Filippo M. Bassi, Miguel Sanchez‐Garcia, Gabriella Sonnante, Pasquale L. Curci, Giovanni Giuliano, Giuseppe Aprea, Asher Pasha, Agata Gadaleta, Ilaria Marcotuli, Stefania Masci, Francesco Sestili, Samuela Palombieri, Davide Scaglione, Michele Morgante, Hana Šimková, Nicholas J. Provart, Simon G. Krattinger, Curtis J. Pozniak, Nathalie Chantret, Anthony Hall, Assaf Distelfeld, Roberto Tuberosa, Marco Maccaferri, Luigi Cattivelli

**Affiliations:** ^1^ Consiglio per la ricerca in agricoltura e l’analisi dell’economia agraria (CREA), Centro di ricerca Genomica e Bioinformatica Fiorenzuola d’Arda Italy; ^2^ Department of Agricultural and Food Sciences (DISTAL) Alma Mater Studiorum – Università di Bologna Bologna Italy; ^3^ Corteva Agriscience Genome Center of Excellence Johnston Iowa USA; ^4^ Earlham Institute Norwich UK; ^5^ Department of Evolutionary and Environmental Biology University of Haifa Haifa Israel; ^6^ King Abdullah University of Science and Technology (KAUST) Thuwal Saudi Arabia; ^7^ Consiglio per la ricerca in agricoltura e l’analisi dell’economia agraria (CREA), Centro di ricerca Cerealicoltura e colture Industriali Foggia Italy; ^8^ UMR AGAP Institut, Univ Montpellier, Inst Agro, CIRAD, INRAE Montpellier France; ^9^ Institute of Experimental Botany, Czech Academy of Sciences, Centre of Plant Structural and Functional Genomics Olomouc Czech Republic; ^10^ Plant Genome and Systems Biology Helmholtz Center Munich Neuherberg Germany; ^11^ Centre for Crop & Food Innovation Food Futures Institute, Murdoch University Murdoch Australia; ^12^ School of Life Sciences Technical University Munich Munich Germany; ^13^ Montana State University Bozeman Montana USA; ^14^ USDA‐Agricultural Research Service Western Regional Research Center Albany California USA; ^15^ USDA‐Agricultural Research Service Edward T. Schafer Agricultural Research Center Fargo North Dakota USA; ^16^ CBGP‐UPM‐INIA‐CSIC, INIA Parque Científico y Tecnológico de la U.P.M Madrid Spain; ^17^ CREA Research Centre for Forestry and Wood Roma Italy; ^18^ National Research Council Institute of Agricultural Biology and Biotechnology Milan Italy; ^19^ Department of Plant Genetics and Production Shiraz University Shiraz Iran; ^20^ Crop Development Centre University of Saskatchewan Saskatoon Saskatchewan Canada; ^21^ Department of Plant Sciences University of Manitoba Manitoba Canada; ^22^ Canadian Grain Commission Winnipeg Manitoba Canada; ^23^ ICARDA Rabat Morocco; ^24^ National Research Council Institute of Biosciences and Bioresources Bari Italy; ^25^ ENEA, Casaccia Research Centre Roma Italy; ^26^ College of Horticulture, Northwest A&F University Yangling Shaanxi China; ^27^ Department of Cell & Systems Biology/Centre for the Analysis of Genome Evolution and Function University of Toronto Toronto Ontario Canada; ^28^ Department of Soil, Plant and Food Sciences University of Bari Aldo Moro Bari Italy; ^29^ DAFNE University of Tuscia Viterbo Italy; ^30^ IGA Technology Services Udine Italy; ^31^ Istituto di Genomica Applicata Udine Italy; ^32^ Dipartimento di Scienze Agro‐alimentari, Ambientali e Animali Università degli Studi di Udine Udine Italy

**Keywords:** annotation, *de novo* assembly, domestication, durum wheat ( *Triticum turgidum* subsp. *durum*), haplotype‐based ancestry analysis, long‐read sequencing, QTLome, transcriptome atlas

## Abstract

Advancements in plant genome sequencing and assembly have enabled the production of increasingly accurate and contiguous genome sequences. Here, we present the chromosome‐level assembly of the durum wheat ( *Triticum turgidum* L. ssp. *durum*, cv. Svevo) reference genome produced using accurate long‐reads, optical mapping and Hi‐C. The new assembly (Svevo Rel.2.0) comprises 263 hybrid scaffolds with an N50 value of 112.3 Mb, arranged into 14 contiguous pseudomolecules spanning 10.4 Gb. The Svevo Rel.2.0 genome assembly was annotated using extensive short‐ and long‐read RNA sequencing data obtained from 60 tissue/treatment combinations. The resulting annotation comprises 68 154 high‐confidence protein‐coding genes, which have been integrated into a comprehensive transcriptome atlas accessible through an eFP browser. Annotation was manually curated for storage protein gene families and for Leucine‐Rich Repeat‐Containing Receptor genes yielding 3763 LRR‐CR loci. The genome assembly's accuracy and completeness were demonstrated by the correct reconstruction of the physical map of *Tg1‐B* (*Tenacious glumes 1*), a locus controlling the free threshing trait located on chromosome 2B that was not assembled in the previous genome release (Svevo Rel.1.0). A wealth of 6621 QTLs/MTAs from the literature were mapped onto Svevo Rel.2.0 to identify QTL hotspots and trait‐specific candidate genes. The ancestry of the durum genome to representative wild emmer populations from North‐Eastern and Southern‐Levant Fertile Crescent assessed by tracing haplotype transmission patterns revealed a clear mosaic pattern. This new durum reference genome, enhanced with advanced annotation and an expression atlas linked to QTLome data, is the most comprehensive tool available for durum wheat genomics.

## 1 Introduction

Durum wheat [ *Triticum turgidum* L. ssp. *durum* (Desf.) Husn, genome BBAA] was domesticated from wild emmer ( *T. turgidum* ssp. *dicoccoides*, Körn. ex Asch. & Graebn. Thell.) through two subsequent domestication and evolution‐under‐domestication steps represented by domesticated emmer ( *T. turgidum* ssp. *dicoccum*, Schrank ex Schübl.) and the more recent *T. turgidum* ssps., with the latest improved by modern plant breeding during the last century (Abbo et al. 2014). Today, durum is the 10th most important crop worldwide in terms of production (Beres et al. 2020), and it constitutes the base ingredient for many of the traditional dishes of the Mediterranean diet, such as pasta, couscous, bulgur, and various baked goods (Sall et al. 2019). Emmer was domesticated from wild emmer by early human settlers who selected plants carrying loss‐of‐function mutations in two homoeologous genes (*TtBtr1‐A, TtBtr1‐B*) that conferred the non‐brittle rachis phenotype (Avni et al. 2017). Domesticated wheat can be either hulled (domesticated emmer wheat and spelt) or free‐threshing (durum and bread wheat plus other marginally cultivated primitive wheats), depending on three main loci: the *Q* locus located on the long arm of chromosome (chr.) 5A (Haas et al. 2019; Sharma et al. 2019), and two *Tenacious glumes* (*Tg*) loci on the short arms of chr. 2A and chr. 2B. In emmer wheat, the dominant alleles of *Tg1‐A* and *Tg1‐B* determine, along with the recessive *q* allele, the non‐free‐threshing phenotype (Faris et al. 2014; Sharma et al. 2019). Free‐threshing likely evolved through two independent events; the first occurred at the tetraploid level via mutations at the *Tg1‐A* and *Tg1‐B* loci; however, because hybrids of free‐threshing tetraploid wheat and *Aegilops tauschii* are hulled, nascent hexaploid wheat must have originally been a hulled crop (McFadden and Sears 1946; Feldman and Levy 2023). Recently, the regulator of the tough glume in *Ae. tauschii* was identified (Dvorak et al. 2026), whereas the origins of the *tg1‐A* and *tg1‐B* alleles remain unresolved. Naked free‐threshing tetraploid wheat forms ( *T. turgidum* ssp. *turgidum*, *carthlicum*, *aethiopicum*, *turanicum*, *polonicum*, and *durum*) gradually replaced domesticated emmer in the Mediterranean Basin, Eastern Europe, and Western/Central Asia, thanks to the ease of threshing, better palatability for the animals, larger grains, kernel vitreousness, and increased productivity (Feldman 2001; Faris et al. 2014). Tetraploid wheat is the donor of the BB and AA genomes of hexaploid bread wheat ( *T. aestivum* L., BBAADD); therefore, tetraploid germplasm represents a direct and valuable source of beneficial alleles to boost genomics‐based improvement in both durum and bread wheat (Ogbonnaya et al. 2013; Peters Haugrud et al. 2025; Fisenko and Dragovich 2024).

The genome assembly of wild emmer wheat, accession Zavitan, was first published by Avni et al. (2017) and later refined using optical maps (Zhu et al. 2019). The first genome assembly of bread wheat, cultivar Chinese Spring (CS), was released in 2018 (International Wheat Genome Sequencing Consortium (IWGSC) 2018), followed by the publication of the first bread wheat pangenome (Walkowiak et al. 2020). The first durum wheat reference genome was released in 2019 (Svevo Rel.1.0, Maccaferri et al. 2019) and it was assembled on the basis of Illumina short‐reads through the DeNovoMAGIC technology (NRGene Inc.), with scaffolding supported by genetic mapping and chromosome conformation capture sequencing (Hi‐C). This assembly resolved the durum genome into 14 pseudomolecules accounting for 9.96 Gb (N50 = 6 Mb), along with an additional 499 Mb of unassigned scaffolds. By today's standards, the Svevo Rel.1.0 assembly would be considered incomplete, and efforts to resolve the assembly and annotations are warranted.

The advent of new genome sequencing platforms capable of delivering longer high‐quality reads (Wenger et al. 2019; Uhlen and Quake 2023), combined with optical genome mapping, has recently prompted the generation of more continuous and accurate assemblies of the wheat reference genomes for two bread wheat varieties. These include improved assembly for CS (Zhu et al. 2021) and a new assembly for the bread wheat variety Kariega (Athiyannan et al. 2022) as well as for the Langdon durum wheat variety (Chen, Li, et al. 2025). Here, we present a new chromosome‐level assembly of the durum wheat reference genome cv. Svevo, Svevo Rel.2.0, produced using the PacBio HiFi long‐read technology coupled with optical mapping and Hi‐C. The Svevo Rel.2.0 genome assembly was fully annotated and empowered with a detailed expression atlas and with the projection of 6621 QTLs from the literature that allow for the identification of QTL‐hotspots and trait‐specific candidate genes. The new assembly allowed the physical map of *Tg1‐B* (*Tenacious glumes 1*), a locus controlling the free threshing trait that was not assembled in Svevo Rel.1.0. It also facilitated an ancestry analysis of the durum genome, allowing for the tracing of the haplotype‐transmission pattern. The new reference genome implemented with advanced annotation, an expression atlas and QTLome landscape represents the most comprehensive genomic tool to date for durum wheat.

## 2 Results

### 2.1 The Svevo Rel.2.0 Genome Assembly

**FIGURE 1:**
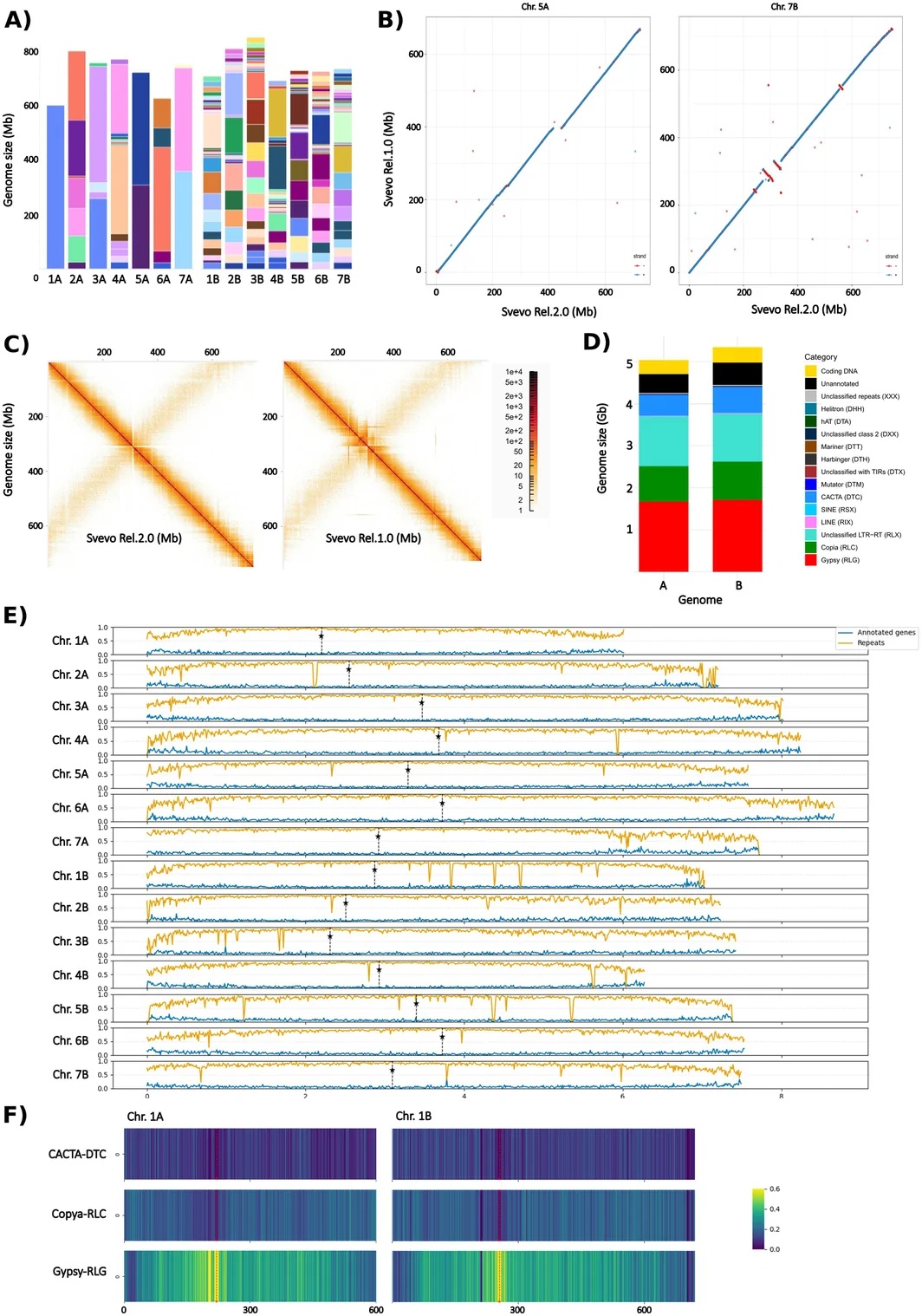
Svevo Rel.2.0 assembly and annotation features. (A) Distribution of HiFi/DLS hybrid scaffolds and non‐captured (unsized) scaffold gaps, by chromosome. Subgenome A and Subgenome B show a seven‐fold difference in contiguity because of the presence of unusually long simple‐sequence repeats. (B) Comparison of Svevo Rel.1.0 and Rel.2.0 assemblies for chr. 5A and chr. 7B showing large insertions and inversions. The plots identify sequence collinearity across the diagonal with blue dots and inversions (+/− alignment orientations) between the two genomes as red dots. Red or blue dots outside the diagonal identify duplications of genome segments. The comparisons of all chromosomes are shown in Figure S3. (C) Intrachromosomal Hi‐C contact matrices for the chr. 7B computed from alignment of Hi‐C reads to the Svevo Rel.2.0 and Rel.1.0 assemblies, highlighting the correction of mis‐assemblies detectable in Rel.1.0. The intensity of the colours corresponds to the interaction frequency. Intrachromosomal and interchromosomal maps of all chromosomes are shown in Figure S1, respectively. (D) Svevo Rel.2.0 annotated repeats and coding sequences summary and relative distribution between the A and B sub‐genomes. (E) Svevo Rel.2.0 chromosomes karyoplot showing the genomic density of annotated genes (blue) and transposable elements (TEs, orange). Densities were computed as the fraction of bases covered in non‐overlapping 1 Mb windows and normalized to values between 0 and 1 for each chromosome. Each chromosome is drawn to scale and aligned on the left, with the vertical axis (0–1) representing the fraction of window coverage. The plot highlights the contrasting distribution patterns of genes, which are enriched toward distal chromosomal regions, and TEs, which are more abundant in pericentromeric regions. Vertical dashed black lines indicate the position of the centromeres, marked with an asterisk. (F) Heatmap showing the genomic distribution of the three most abundant TE families (Copia‐RLC, Gypsy‐RLG, and CACTA‐DTC) across Svevo Rel.2.0 durum wheat chromosomes Chr1A and Chr1B. TE densities were calculated as the fraction of bases covered within non‐overlapping 1 Mb windows. Colour intensity reflects the local density of each TE family, with colour scale adjusted to a maximum fraction = 0.6. Vertical dashed red lines indicate the position of the centromeres.

The PacBio HiFi long‐reads (32× coverage) were assembled into 813 primary contigs (contig N50 = 30.8 Mb, sum = 10.428 Gb) and combined with 502 optical genome maps (map N50 = 67.3 Mb, sum = 10.462 Gb) to generate hybrid scaffolds. These were ordered and oriented through Hi‐C data, resulting in a genome assembly consisting of 263 hybrid scaffolds (scaffold N50 = 112.3 Mb), for a total genome length of 10.387 Gb. We were unable to incorporate 19.8 Mb of sequence into the chromosome assembly, which remains as unassigned scaffolds (Table 1 and Table S1). The number of scaffolds used to assemble individual chromosomes ranged from 1 (chr. 1A) to 41 (chr. 6B), with B chromosomes containing more scaffolds than their A homeologs (Figure 1A, Table S1b). Aligning the 14 pseudomolecules with optical maps revealed a mis‐orientation of a 623 kb segment in chr. 7B, caused by a nearly identical 67 kb repeat flanking both ends, and an inversion and interchange of two segments of 471 and 489 kb in chr. 2A that were manually corrected. The final genome assembly, Svevo Rel.2.0, represents a substantial improvement over Svevo Rel.1.0 in terms of both length and number of scaffolds (total assembly = 9.96 Gb, N50 = 6 Mb, unassigned scaffolds = 499 Mb; Maccaferri et al. 2019) (Table 1 and Table S1c). The comparison of the two releases revealed several structural differences. Most of the regions' insertions in Rel.2.0 as compared to Rel.1.0, represented new contigs that were not previously assembled, whereas inversions were confirmed by optical maps as scaffolds that were mis‐oriented in Rel.1.0. For example, a 29.7 Mb insertion was detected in the revised assembly of chr. 5A, and two major inversions were detected on chr. 7B (Figure 1B). High‐resolution Hi‐C contact maps generated after re‐mapping the Hi‐C reads to the final, post‐curated Rel.2.0 and Rel.1.0 assemblies clearly showed that inversions and mis‐assemblies on chr. 3A, 3B, 4A, 4B, 5A (Figure S1), and 7B (Figure 1C) were corrected in Rel.2.0 compared to Rel.1.0. Large insertions and inversions were confirmed in Svevo Rel.2.0 using molecular markers ordered along the durum wheat consensus map (Maccaferri et al. 2015). On the basis of the intersections of diagonals and anti‐diagonals in the Hi‐C contact matrices, reflecting *Rabl* configuration of *Triticeae* chromosomes (Cowan et al. 2001), the intervals corresponding to putative centromeric domains were identified in all but four chromosomes (Table S2, Figure S2). Next, we aligned and compared Svevo Rel.2.0 to the wild emmer (Zavitan WEWSeq v2.0, Zhu et al. 2019) and the bread wheat (CS, IWGSC RefSeq v2.1, Zhu et al. 2021) genomes. This analysis highlighted several major rearrangements, which were more pronounced when comparing Svevo Rel.2.0 to Zavitan than vs. CS (Figure S3). There was an exception to this where rearrangements on chr. 4B and chr. 6B detected between Svevo and CS were conserved between Svevo and Zavitan.

**TABLE 1:** Main statistics of the genome assembly and the gene annotation of Svevo Rel.2.0 in comparison with Svevo Rel.1.0, Zavitan WEWSeq v2.1, and Langdon.

Genome assembly	Svevo Rel.2.0	Svevo Rel.1.0	Zavitan^a^	Langdon^b^
Total assembly size (Gbp)	10.40	10.45	10.66	10.47
Sequence assigned to chromosomes (Gbp)	10.39	9.87	9.99	10.38
Number of contigs	813	474 837	491 383	2234
N50 Contig length (Mbp)	30.74	0.056	0.57	41.18
Longest Contig (Mbp)	193.22	0.475	0.61	295.80
Number of scaffolds	263	129 464	149 252	724
N50 Scaffold length (Mbp)	112.32	5.97	72.63	751.30
BUSCO completeness	99.94%	98.49%^c^	98.40%	98.80%
Annotation				
Number of annotated genes	216 555	369 963	338 361	144 055
Number of high‐confidence protein‐coding genes	68 154	66 559	67 182	68 342
Total repeat sequences (Gbp)	8.74	8.60	8.64	9.24
Retrotransposons (Gbp)	7.52	7.36	7.39	7.06
DNA transposons (Gbp)	1.16	1.19	1.21	1.83

^a^ Contig statistics were obtained from Avni et al. (2017), whereas scaffolds were obtained from Tingting Zhu et al. (2019).

^b^ Statistics from Chen, Li, et al. (2025); Chen, Chen, et al. (2025).

^c^ Recalculated in this study.

### 2.2 Genome Annotations

The Svevo Rel.2.0 assembly was annotated to delineate repeats and gene models. Transposable elements (TEs) were identified and classified by similarity search against the Triticeae‐specific section of the REdat_9.7 PGSB transposon library (Spannagl et al. 2016; Walkowiak et al. 2020). TEs accounted for 83.9% of the assembly (Table S3), being more abundant in the pericentromeric regions and relatively evenly distributed across the two subgenomes (Figure 1D,E and Figure S4). Among the different families, long terminal repeats (LTR) elements occupied 7.49 Gb (71.9%) of the whole genome, with the two major superfamilies within the LTR elements, Gypsy (RLG, 32.6%) preferentially enriched in the centromeric region, whereas Copia (RLC, 16.9%) did not show similar enrichment along the chromosomes (Figure 1F and Figure S5). DNA transposons were less abundant, occupying only 11.2% of the genome, with the CACTA superfamily being the most abundant one (10.7% of the genome).

The annotation pipeline combined evidence from repeat annotation, newly generated short and long read RNA sequencing data, hexaploid wheat gene models, and protein reference sequences to predict transcript sequences and provide functional annotation. For transcriptomic data, RNA‐seq was conducted on a total of 174 samples, including RNA‐seq from 30 organs, tissues, and developmental stages under standard growth conditions (Table S4a), whereas 28 samples corresponding to biotic, abiotic, and nutrient stress conditions (Table S4a) were incorporated in the pipeline as long read sequences. The isoforms were verified using Oxford Nanopore Technology (ONT) long‐read cDNA sequencing for 36 combinations of the 174 RNA samples (Table S4b). In addition, public durm wheat short‐read RNA‐seq data (Maccaferri et al. 2019) and publicly available bread wheat IsoSeq transcripts (Clavijo et al. 2017) were incorporated. In total, the annotation process leveraged approximately 6 billion RNA‐Seq read pairs and 82 million long transcriptomic reads.

These combined datasets were used to annotate 68 154 high‐confidence (HC) protein‐coding genes (Table S5, Figure S6). Additionally, 93 888 protein‐coding loci were classified as low‐confidence (LC) genes, due in part to partially supported gene models, gene fragments, and non‐conserved orphan genes. Furthermore, 4256 HC TE genes, 3241 long non‐coding RNAs, and 58 pseudogenes (prolamin genes only) were also identified. The extensive transcriptomic dataset enabled the detailed characterization of alternative splicing events and untranslated regions (UTRs), revealing that HC coding genes averaged 2.12 transcripts per gene, with 97% of gene models annotated with both 5′ and 3′ UTRs (Table S5). The annotation of the Svevo Rel.2.0 genome also revealed 129 514 putative transcription factor binding sites (TFBS) in HC genes (q‐value < e‐05) and 1 804 712 CpG‐islands, corresponding to a mean distribution of a CpG island every 5761 bp (Dataset 1). Among the 68 154 HC genes predicted in Svevo Rel.2.0, 46 887 loci showed a one‐to‐one correspondence with HC loci in Svevo Rel.1.0. An additional 10 233 loci partially overlapped with either partially or mis‐annotated HC and LC loci in Rel.1.0 (Figure S7), whereas 15 388 loci were not represented in the previous annotation, including major well‐known developmental regulatory genes such as *Ppd‐B1* (Würschum et al. 2019) and *VRN2/ZCCT‐A2* (Hirsz et al. 2026) (Figure S7D). These new HC annotations are countered by about 11 000 HC Rel.1.0 genes reclassified as LC in Svevo Rel.2.0 (Dataset 2). The completeness of the Svevo Rel.2.0 assembly and annotation was quantified by using the Benchmarking Universal Single‐Copy Orthologs (BUSCO, Simão et al. 2015). A total of 4468 (99.94%) BUSCO genes (v4.0.6, poales_odb10, Manni et al. 2021) were identified as complete, compared with 98.49% completed BUSCO genes in Rel.1.0. Of these, 91.26% of BUSCO genes were present in the expected two copies, representing a significant increase from 82.09% of the Rel.1.0 annotation, whereas the number of single‐copy BUSCO genes decreased to 140 (2.86%) from the 602 (12.30%) in Rel.1.0 annotation (Table S6). The OMArk analysis (Nevers et al. 2024) revealed a smaller proportion of missing genes (0.23% vs. 3.18%) and a lower proportion of unknown genes (13.74% vs. 34.37%) in the new release as compared to Svevo Rel.1.0 (Figure S8). The completeness and accuracy of the Svevo Rel.2.0 genome assembly were further validated by verifying the presence of 216 experimentally characterized genes (Maccaferri et al. 2019) and 6241 Triticeae validated gene records from GrainGenes. Compared to the previous Svevo Rel.1.0 version, Rel.2.0 exhibited a higher number of mapped genes, along with improved sensitivity and precision at both locus and exon level in gene annotation, evaluated on the basis of query features that agreed or were missed in the corresponding reference annotation features (Table S7).

Gene models were named as TrturSVE1A02G00000010, where 1A and 02 specify the location of the gene on chr. 1A and the second annotation version, respectively. All annotated features are available in the durum wheat Genome Browser (https://graingenes.org/jb/?data=/ggds/whe‐svevo2). Correspondence among gene IDs in the Rel.2.0 and Rel.1.0 is provided in Dataset 2. The newly generated transcriptomic dataset (Table S4) was mapped to the new SvevoRel.2.0 annotated genome to build a transcriptome atlas of durum wheat gene expression (Datasets 3–5). To explore and compare the gene expression, an electronic Fluorescent Pictographic Browser dedicated to durum wheat was established (Durum Wheat eFP Browser, https://bar.utoronto.ca/efp_durum_wheat/cgi‐bin/efpWeb.cgi), which allows users to visualize and download gene‐specific expression data as electronic fluorescent pictographs or histograms. The eFP Browser is organized in three sections, showing data across organs (root, leaf, spike, and seed) and developmental stages, upon abiotic and nutritional stress treatments (drought, cold, heat, salt, and low nitrogen), and in response to *Fusarium graminearum* infection (Figure S9).

### 2.3 Manual Curation of Prolamin Genes

**FIGURE 2:**
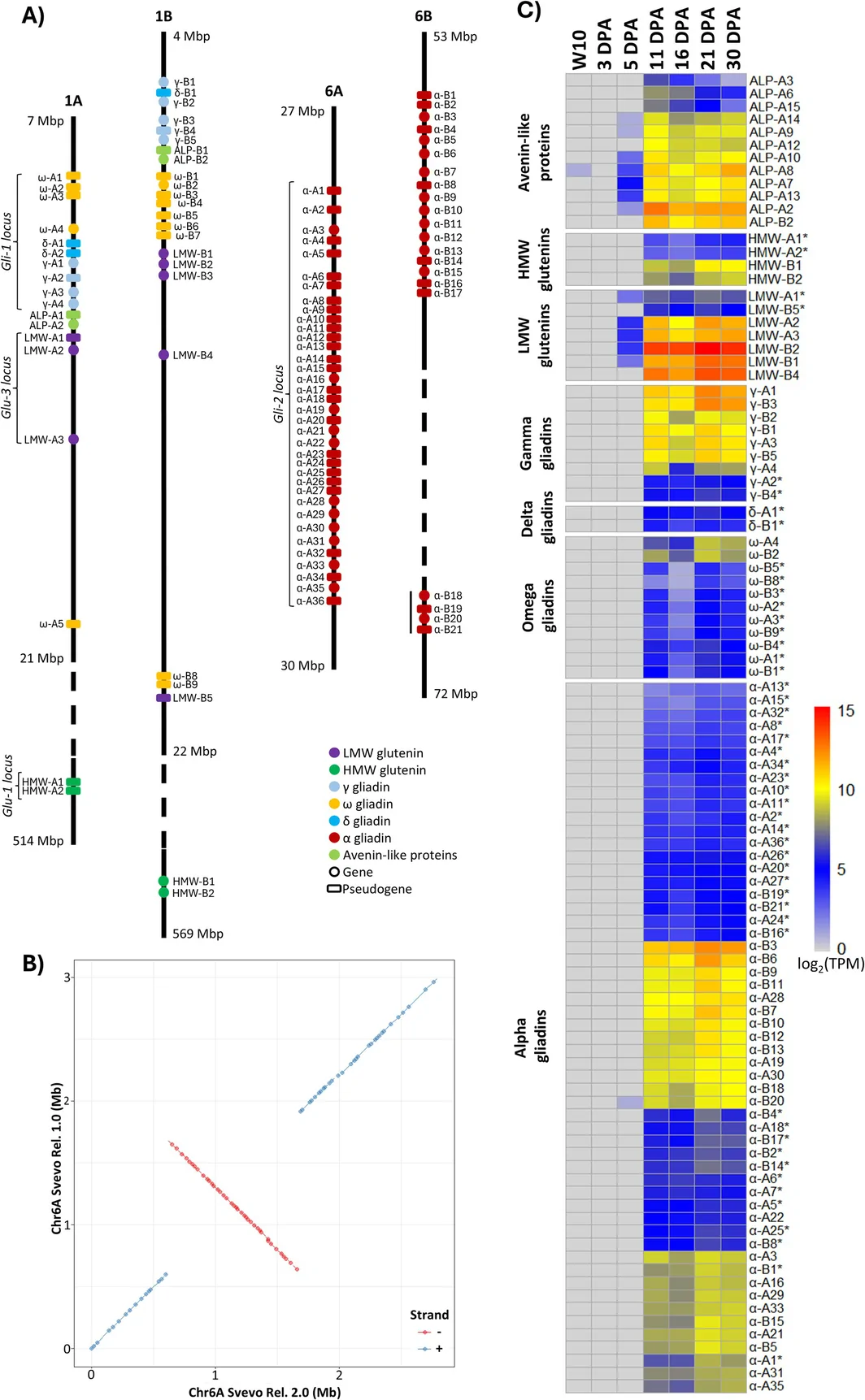
Structural organization of prolamin loci in Svevo Rel.2.0. (A) Genome structure of prolamin genes. Genes and pseudogenes are represented by circles and rectangles, respectively. Black bar at the end of chr 6B indicates the bipartite structure at the *Gli‐2* locus. Distances are not in scale. (B) The alignment between Svevo Rel.2.0 and Rel.1.0 revealed an inversion on chr 6A spanning ca. Mb in size. (C) Heatmap of prolamin gene expression during durum wheat grain development. Expression levels (TPM) obtained by RNA‐seq across developmental stages (W10 ovaries to 30 days post‐anthesis, DPA). The data are log_2_‐transformed. Genes are grouped by prolamin class: α‐, γ‐, δ‐, and ω‐gliadins, HMW and LMW glutenins, and avenins (ALP). Pseudogenes are indicated with an asterisk (*). The colour gradient reflects expression intensity, ranging from low (blue) to high (red), whereas grey indicates no detectable expression (0 TPM). Genes with a sum of TPM among all stages < 10 were filtered out.

Major determinants of wheat end‐use functionality include members of the prolamin superfamily, namely gliadins (α‐, γ‐, ω‐, and δ‐gliadins), high‐ and low‐molecular weight glutenin subunits (HMW‐GS and LMW‐GS, respectively), as well as avenin‐like proteins (ALPs). These proteins are encoded by multigene families clustered in several loci (Huo, Zhang, et al. 2018; Huo, Zhu, et al. 2018; Wang, Li, et al. 2020; Paris et al. 2021) and are characterized by highly repetitive sequences, two features that make their correct assembly challenging. Manual curation of the Svevo Rel.2.0 annotation identified 112 prolamins located on chr. 1A, 1B, 4A, 6A and 6B, and 7A, including 57 α‐, 9 γ‐, 14 ω‐ and 3 δ‐gliadins, 4 HMW‐GS and 8 LMW‐GS, and 17 ALPs (Figure 2A, Table S8). A significant proportion (51%) was classified as pseudogenes, including 34 α‐, 2 γ‐, 12 ω‐, and 3 δ‐gliadins, 2 HMW‐ and 2 LMW‐glutenin, and 3 ALPs. In Svevo Rel.1.0, 124 sequences were annotated as prolamin genes, although only 102 of them were correctly mapped in the expected chromosomal regions. Chromosome alignment between Svevo Rel.2.0 and Rel.1.0 highlighted an inversion on chr. 6A spanning around 1 Mb in size (28.228–29.336 Mb) that included 13 α‐gliadins among the 36 annotated on this chromosome (Figure 2B, Table S8). In addition, the Svevo Rel.2.0 assembly also includes prolamin genes not previously resolved because of the presence of repetitive regions (e.g., ω‐gliadins and HMW‐GS genes). Furthermore, Svevo Rel.2.0 assembly confirmed the bipartite structure at the *Gli‐B2* locus on chr 6B (Figure 2A, Halstead‐Nussloch et al. 2021), and revealed variation in the position of cysteine residues putatively implicated in polymer formation between the LMW‐GS encoded by the A and B genomes (Figure S10). Expression analysis (Figure 2C) showed that for most genes, expression can be detected at either 5 or 11 days post‐anthesis (DPA) and that is then maintained until the last time‐point examined (30 DPA). As expected, expression was generally low in the case of pseudogenes.

### 2.4 Manual Curation of Leucine‐Rich Repeat‐Containing Receptor (LRR‐CR) Gene Families

A total of 3763 LRR‐CR loci were identified in Svevo Rel.2.0 (Tables 2, S9, and S10) through an iterative annotation process combining manual curation and homology‐based transfer, following the method described in Gottin et al. (2021). Among these, 2461 genes were classified as Nonsense Mutation‐Containing Genes (NsMCG). Each NsMC gene is annotated with information regarding the predicted impact of nonsense mutations on transcription, including frameshifts, premature stop codons, aberrant splice sites, and absence of start or stop codons. These annotations enabled reconstruction of the full‐length protein sequences for each NsMCG, inference of their putative structures prior to mutation, and classification on the basis of conserved domain architecture. This information is essential for allele discovery strategies aimed at comparing susceptible and resistant varieties, as well as for investigating the evolutionary trajectories of these NsMC genes. Notably, we found a significantly higher proportion of NsMCGs (65%) compared to rice, where only 30% had been previously reported (Gottin et al. 2021).

**TABLE 2:** Summary statistics of predicted LRR‐CR genes and their comparison with Svevo Rel.1.0 annotation.

	LRR‐CR in Svevo Rel.1.0			LRR‐CR in Svevo Rel.2.0		
	HC	LC	total	Functional	NsMCG	total
Total N° of genes	1899	226	2125	1302	2461	3763
NLR	918	106	1024	603	1580	2183
LRR‐RLK	614	22	636	493	431	924
LRR‐RLP	367	98	465	206	450	656
Cumulated protein size (a.a)	1 692 503	124 177	1 816 680	1 256 087	2 218 889	3 474 976
Total number of LRR motifs	25 547	1803	27 350	19 356	33 935	53 291

Abbreviation: NsMCG, Non‐sense mutation containing genes.

The LRR‐CR proteins from Svevo Rel.2.0 were classified as NLRs, LRR‐RLKs, and LRR‐RLPs on the basis of their key domains, and this classification was compared with analysis of Svevo Rel.1.0 (Tables 2, S9, and S11). The newly curated LRR‐CR annotation was used for Rel.2.0, whereas the standard automatic annotation (HC and LC gene models) was employed for Rel.1.0. The number of LRR‐CR genes was approximately 77% higher for Rel.2.0 compared to Rel.1.0 (3763 vs. 2125), the cumulative protein length increased by 90% (3 474 976 vs. 1 816 680), and the cumulative length of NB‐ARC domains was nearly twice as high (571 366 vs. 262 721). These data demonstrate the high quality of the iterative annotation approach (Gottin et al. 2021) that combines manual curation and homology‐based transfer, which was used for Svevo Rel.2.0.

### 2.5 The Completeness and Accuracy of Svevo Rel.2.0 Reveals the Molecular Mechanism Underlying the *Tg1‐B* Locus

**FIGURE 3:**
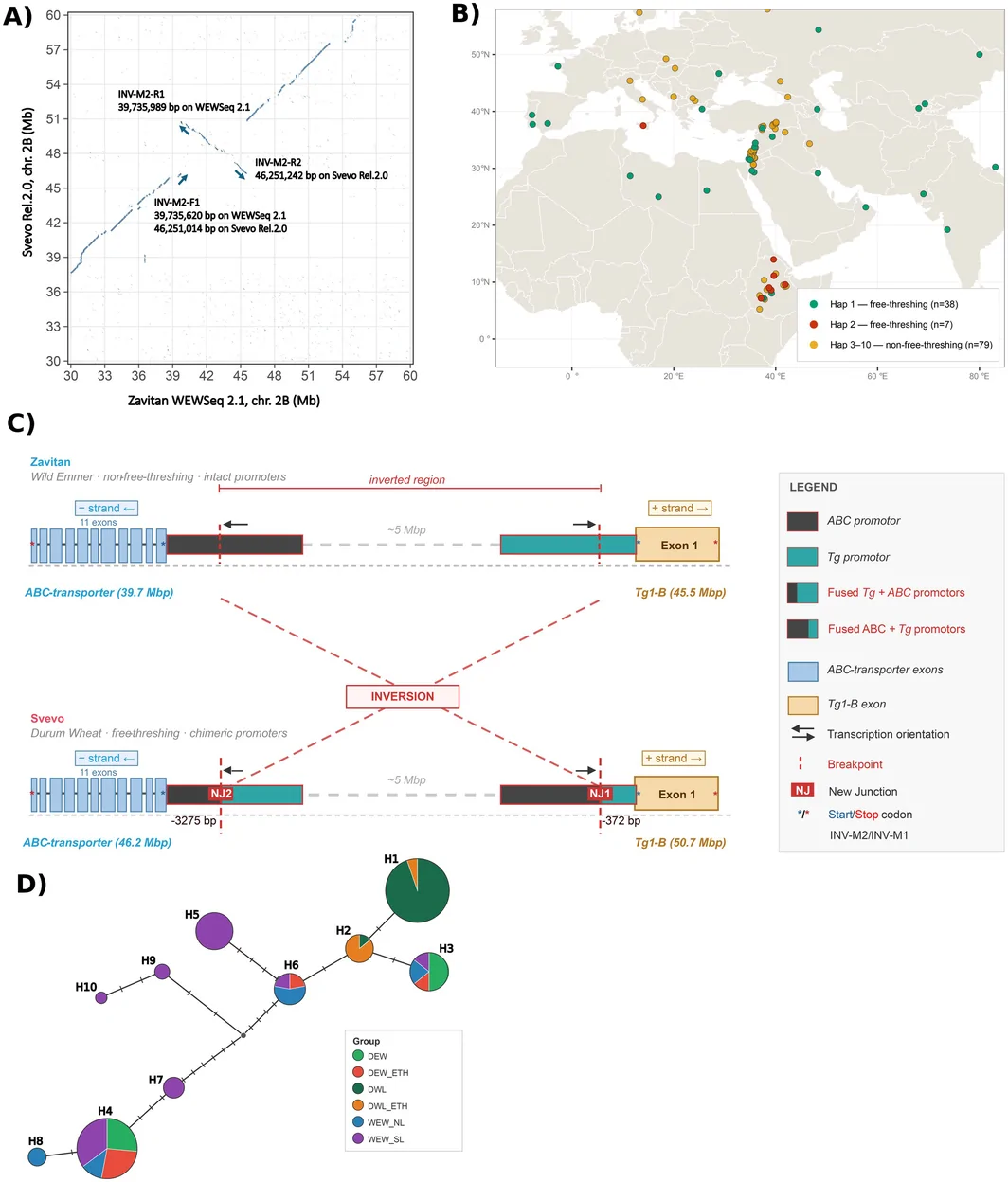
Structural variation and haplotype distribution at the *Tg1‐B* locus. (A) Alignment between the Zavitan WEWSeq 2.1 and Svevo Rel.2.0 genome assemblies for Chromosome 2B (30–60 Mb). The plot reveals a~5 Mb inversion between the two genomes. Blue arrows indicate the locations of the INV‐M2 assays primers on the two assemblies relative to the ~5 Mb structural rearrangement. (B) Geographic haplotype distribution of the *Tg1‐B* locus across accessions of Wild Emmer Wheat (WEW), Domesticated Emmer Wheat (DEW), and Durum Wheat (DW). Green and red dots represent the free‐threshing haplotypes (Hap 1, *n* = 38; Hap 2, *n* = 7), whereas yellow dots represent the non‐free‐threshing haplotypes (Hap 3–10, *n* = 79). (C) Schematic representation of promoter fusion driven by inversion at the *Tg1‐B* locus on Chromosome 2B. In Zavitan (wild emmer, non‐free‐threshing), the promoters for the ABC‐transporter and the *Tg1‐B* exon are intact. A~5 Mbp inversion in Svevo (durum wheat, free‐threshing) results in new junctions (NJ1 and NJ2). This structural rearrangement creates chimeric regulatory landscapes, forming two fused promoter regions distinct from the original wild emmer configurations. Specifically, a fused *Tg1‐B* + *ABC* promoter drives *Tg1‐B* in Svevo, whereas a fused *ABC* + *Tg1‐B* promoter drives the ABC‐transporter. (D) TCS haplotype network of *T. turgidum* accessions on the basis of 24 SNPs at Chr2B:50790476–50 792 842 (Svevo Rel.2.0). Node area is proportional to haplotype frequency (scale inset). Pie segments indicate population group composition: Durum Wheat Landraces (DWL; teal), Ethiopian Durum Wheat Landraces (DWL_ETH; orange), Domesticated Emmer Wheat (DEW; green), Ethiopian Domesticated Emmer Wheat (DEW_ETH; red), Southern Levant Wild Emmer Wheat (WEW_SL; purple), and Northern Levant Wild Emmer Wheat (WEW_NL; blue). Tick marks on edges denote mutational steps. H2 and H3 share identical SNP haplotypes and are separated by the chr2B inversion (H2: Inversion present; H3: Absent).

The new genome assembly allowed anchoring and dissecting of a QTL previously identified for the tenacious glume phenotype on the short arm of chr. 2B (*Tg1‐B*) using the *T. turgidum* ssp. *durum* (Svevo cv.) × *T. turgidum* ssp. *dicoccoides* (Zavitan accession) recombinant inbred line population (Avni et al. 2014; Lev‐Mirom, Avni, et al. 2026). Alignment of the *Tg1‐B* locus in Zavitan (WEWSeq V2.1) with the orthologous region in Svevo (Rel.2.0), using genome‐anchored locus‐specific markers, revealed a 5 Mb inversion. This structural rearrangement is characterized by a reversed order of markers between IWB29368 and IWB68977 (Figure S11). Comparative genomic alignment between the Zavitan WEWSeq 2.1 and the Svevo Rel.2.0 assemblies facilitated the precise identification of the breakpoints flanking this ~5 Mb inversion (Figure 3A). This structural variation, initially indicated by the genetic map (Figure S11), was further validated through the development of two locus‐specific molecular markers (INV‐M1 and INV‐M2), which confirmed the inverted orientation between the two genomes (Figure 3A). To investigate the role of the ~5 Mb inversion, we initially explored the occurrence of this structural rearrangement in a panel of 285 accessions with different degrees of threshability on the basis of their domestication level by using a specific marker assay on the inversion (INV‐M1, Figure 3A Table S12). The results revealed that the ~5 Mb inversion on chr. 2B is strongly associated with the free‐threshing phenotype and suggests that it played a key role in the transition from hulled to free‐threshing wheat during domestication.

More recently, the gene *AET2Gv20109500* was identified as the regulator of the tough‐glume (*Tg*) trait in the diploid species *Ae. tauschii*, with a mutated allele at the *Tg1‐D* locus driving the free‐threshing phenotype in hexaploid wheat (*tg1‐D*) (Dvorak et al. 2026). We performed a BLAST analysis using *AET2Gv20109500* as a query against tetraploid reference genomes. The B‐genome ortholog (designated *Tg1‐B*) was found on an unanchored scaffold in the Svevo Rel.1.0 assembly but was successfully mapped to chr. 2B in Svevo Rel.2.0 (Gene ID: *TrturSVE2B02G00464940*). Notably, the breakpoints of the ~5 Mb inversion were precisely mapped to 378 bp upstream of the *Tg1‐B* start codon (INV‐M1) and 3275 bp from an ABC‐transporter gene (*TrturSVE2B02G00463250*; INV‐M2). In Svevo, this structural rearrangement resulted in the formation of two chimeric promoters, creating regulatory configurations distinct from the ancestral wild emmer wheat (Zavitan) architecture (Figure 3C). Next, to identify the origin of the inversion, we performed a haplotype analysis of *Tg1‐B* using a core collection of 159 previously described genotypes and corresponding resequencing data (Lev‐Mirom, Ashkenazy, et al. 2026). This analysis fully validated the linkage between the ~5 Mb inversion in the *Tg1‐B* locus and the free‐threshing phenotype (Figure 3B, Table S13). Furthermore, we identified a rare progenitor haplotype, defined as being closely related to the wild‐type (tough glume) but harbouring the causative ~5 Mbp inversion (free‐threshing). This specific haplotype was restricted to seven genotypes, six of which are classified as Ethiopian wheat (Figure 3D).

### 2.6 The Projection of the Durum Wheat QTLome Onto Svevo Rel.2.0 Unveils QTL‐Hotspots and Trait‐Specific Candidate Genes

Although the first version of the durum QTLome contained 2105 QTLs from 120 studies published up to 2018 (Maccaferri et al. 2019), the current version leverages the Svevo Rel.2.0 assembly to incorporate 6621 QTLs from 238 studies published by 2024, primarily GWAS‐based (4504 QTLs) (Table S14). Most phenotypic traits documented in the literature were considered, including key traits of domestication, developmental, and morphological traits like phenology and root architecture, responses to biotic and abiotic stresses, and crop performance‐related factors such as yield and components, and quality. The contiguity and completeness of Svevo Rel.2.0 impacted the number of molecular markers correctly anchored onto the genome, resulting in high accuracy and resolution of QTLs projected onto the genome. In Svevo Rel.1.0, 3370 SNPs from the Illumina 90 K Wheat array (Wang et al. 2014) mapped uniquely on the unknown chromosome, this number was reduced to 165 in Svevo Rel.2.0. In total, 53 QTL peak markers, 60 left and 63 right flanking markers previously mapped on the unassembled portion of Svevo Rel.1.0, were assigned to specific chromosomes in Svevo Rel.2.0, allowing anchoring of the corresponding QTLs onto the genome assembly.

To illustrate the potential of the new durum QTLome, we identified QTL hotspots for several agronomically relevant traits, including disease resistance as a qualitative trait with a low/moderate environmental effect and thousand kernel weight (TKW) and root morphology as complex quantitative traits with a high environmental effect (Table S15). We verified the coincidence of some QTLs with well‐studied genes with validated phenotypic effects from functional studies, thus bringing additional evidence to the QTL effect. In hotspots encompassing a few HC genes, a further refining of candidates could be pursued on the basis of the gene expression, thus proving the usefulness of the QTLome coupled with the expression atlas to define and refine genomic regions for specific traits.

Forty‐six hotspot regions for response to diseases and pest resistance were defined. They were composed of 3 to 19 QTLs, with a minimum overlapping region ranging from 0.9 to 8.9 Mb, and are located on all chromosomes except 3A and 4B. Co‐location of disease resistance hotspots with cloned R genes originally mapped in tetraploid wheat was verified for *Sr13* (Disease_36, chr. 6A, Zhang et al. 2017) and *Lr14* (Disease_46, chr. 7B, Kolodziej et al. 2021). Then, the inspection of hotspots for the type of diseases and candidate genes highlighted the potential interest of a few regions. Indeed, four hotspots (Disease_3, _8, _23, _29) included loci associated with resistance to five different diseases (Table S15), and nine hotspots contain more than 10 NsMCGs, with the telomeric Disease_17 on chr. 4A and Disease_40 on chr. 7A encompassing 45 and 42 NsMCG, respectively (Table S17). NsMCG may point out alternative resistance alleles effective within the tetraploid germplasm. Analysing the expression profile of candidate genes upon Fusarium infection highlighted other potentially relevant QTL hotpots, among them seven containing at least two QTLs for Fusarium Head Blight (FHB) resistance (Disease_1, _6, _7, _13, _20, _22, _40). For instance, the hotspots Disease_20 encompass two genes (*TrturSVE5A02G01361870* and *TrturSVE5A02G01361880*), which were 215‐fold and 58‐fold upregulated in response to Fusarium, respectively, out of the three annotated as disease‐related genes (Table S16).

**FIGURE 4:**
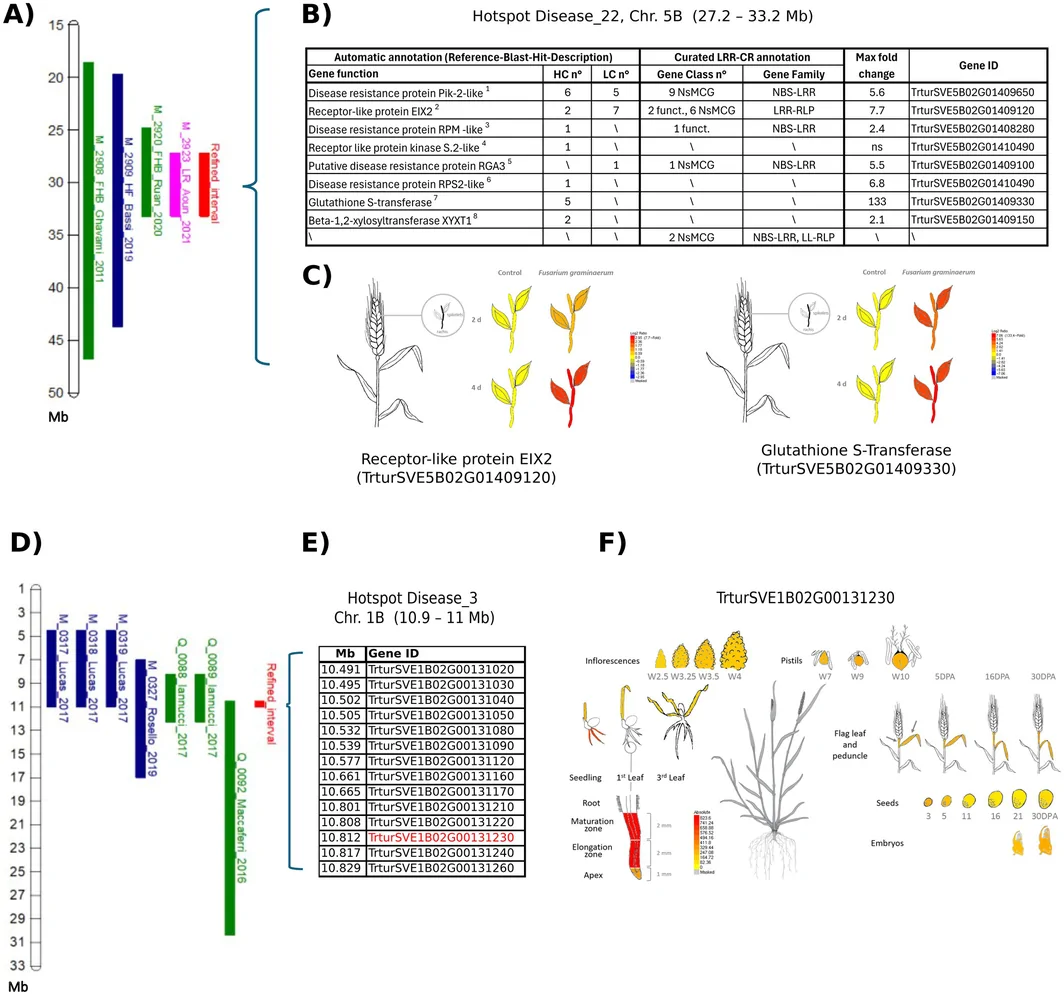
The QTL hotspots Disease_22 and Root_3. (A) The telomere of the short arm of chr. 5B is shown together with 4 overlapping QTL, represented as coloured bars (blue for QTL for Tan Spot resistance, green for QTLs for Fusarium resistance, and red for the QTL for Leaf rust resistance), which define the QTL hotspots Disease_22 with a minimal interval region of about 6 Mb (from 27.2 to 33.2 Mb). (B) Summary of the disease‐related gene functions (both HC and LC), including NsMCGs present in the minimal interval region, and ID of genes with maximum expression value in response to Fusarium infection (in the right column). (C) The pictogram depicting the expression of two candidate genes, the GST *TrturSVE5B02G01409330* and the resistance gene *TrturSVE5B02G01409120*, derived from the Durum Wheat eFP Browser. (D) The telomeric region of the short arm of chr. 1B is represented together with 7 overlapping QTL that define the QTL hotspot Root_3, with a minimal interval region of 0.5 Mb (from 10.49 to 11 Mb, marked in red) encompassing 14 HC genes. (E) HC genes in the hotspot minimal interval region. (F) Expression of *TrTurSVE1B02G00131230*, which encodes for a vacuolar‐type H(+)‐ATPase C3 (VHA‐C3) transmembrane subunit.

Interestingly, the hotspot Disease_22 encompasses a cluster of five *Glutathione S‐Transferase* (GST), two G*lycosyltransferase* (GSST), a cluster of 11 disease resistance protein Pik‐2‐like genes (6 HC), nine receptor‐like protein EIX2 (2 HC), and 18 NsMCGs (Tables S15 and S17). The two genes encoding GSTs (*TrturSVE5B02G01409240* and *TrturSVE5B02G01409330*) were 25‐ and 134‐fold upregulated upon *Fusarium* infection, respectively, whereas a 2.4–7.7‐fold upregulation was shown by a few disease resistance genes (Figure 4A–C, Tables S15 and S16). Notably, the hotspot Disease_2 is sustained by four QTLs, all added with the new version of the QTLome. A role in *Fusarium* response for GST and GSST enzymatic functions was previously suggested by the integration of metaQTL analysis with transcriptomics and proteomics (Zheng et al. 2021) and by the cloning of *Fhb7* from *Thinopyrum ponticum* (Wang, Sun, et al. 2020). In tetraploid wheat, strong genetic resistance to FHB has not been reported so far, and only minor QTLs have been mapped (Viviani et al. 2025). The *cv*. Svevo is largely susceptible to FHB; thus, these candidate genes could contribute to the plant response to *Fusarium* despite being insufficient for providing full resistance.

A total of 448 QTLs associated with TKW were compiled; among them, 107 QTLs were grouped into 26 hotspots with a size ranging from 1.5 to 9.3 Mb (Table S15). Candidate genes with a functional role on grain weight in wheat or rice and relevant expression data pointed out QTL hotspots with a potential interest. For instance, the hotspot TKW_11 on chr. 3B encompasses the gene *TrturSVE3B02G00956290* (Table S15), which encodes a PLATZ transcription factor specifically expressed in grains at 5 and 11 DAP. This gene shows high similarity to *OsFL3* in rice, whose knockout results in reduced grain weight (Guo et al. 2022). Manual annotation of a cluster of eight sucrose:sucrose 1‐fructosyltransferase genes under the hotspot TKW_12 on chr. 4A revealed a sucrose:sucrose 1‐fructosyltransferase (*1‐SST*), two copies of a sucrose:fructan 6‐fructosyltransferase (*6‐SFT*), and five copies of vacuolar invertase genes. A high, stem‐specific expression level was observed for the *6‐SFT TrturSVE4A02G01119200*, whereas the paralogue *TrturSVE4A02G01119230* and the invertase *TrturSVE4A02G01119300* were highly expressed in pistils and kernels at early developmental stages (Figure S12). In bread wheat, most of the carbon flux from sucrose to fructan is mediated by 6‐SFT (Vijn and Smeekens 1999), for which allelic variations and gene expression were related to kernel weight and stem fructan concentrations, respectively (Yue et al. 2015; Xue et al. 2008). Overall, these genes are known to be associated with variability in water‐soluble carbohydrate content (Gaur et al. 2022), which plays an important role in buffering wheat grain yield under terminal drought (Ovenden et al. 2017). Notably, TKW_12 included many QTLs for stress‐induced variation in seed weight as well as QTLs for Grain Yield (GY) and GY stability indices (Table S15; Sukumaran et al. 2018; Negisho et al. 2022, Zaïm et al. 2024), highlighting the role of this region to achieve stable grain weight, likely on the basis of a positive impact of fructan metabolism. Notably, for both TKW_11 and TKW_12, most of the contributing QTLs were added with the new version of the QTLome, and some of them defined the narrow size of the confidence interval.

A total of 626 QTLs related to root morphology and function were compiled from the literature, with 208 of them clustering into 43 hotspots distributed across all chromosomes except 7B (Table S15). Correspondences with genes known for their role in root morphology/development were found for 22 hotspots, supporting the role of the underlined QTLs in root architecture. For instance, the hotspot Root_25 on chr. 4B (6 QTLs, 9 Mb, 67 HC genes) harboured the gene *TrturSVE4B02G01227910* that is specifically expressed in roots and is homologous to the gene *TaMORb* (*MORE ROOT* in wheat) that determines crown root initiation in bread wheat (Li et al. 2016) (Figure S13). Interestingly, the homologous gene in rice *OsLBD3‐2/OsCRL1* (Os03t0149100) showed a similar role (Luo et al. 2022). Notably, Root_25 was defined by two QTLs in the old version of the QTLome; four additional QTLs were included in the new version, and two of them contribute to defining the minimum region in which the *TaMORb* candidate gene was identified. Other interesting examples are hotspots Root_38 and Root_42 in homoeologous positions on the long arms of chr. 6A and 6B, respectively. Root_38 harbours a major QTL on root growth angle repeatedly described in diverse tetraploid genetic backgrounds, showing beneficial effects under drought (Maccaferri et al. 2016; Alahmad et al. 2019; Sciara et al. 2025). The two homeologous regions included *TrturSVE6A02G01686810* in chr. 6A and *TrturSVE6B02G01842660* on chr. 6B, both specifically expressed in the maturation zone of the juvenile root (Figure S14). These genes code for a protein highly similar to the OsDH1/OsLOB16 rice protein (*Os02t0820500*), which contains a binding motif to WOX11, a WUSCHEL‐related homeobox domain transcription factor key regulator of crown root growth, lateral root initiation, root hair formation, and response to abiotic stresses in rice (Jiang et al. 2017). Another intriguing region is Root_3 on chr. 1B, where expression profiling clearly pointed out a candidate gene (Figure 4D–F). Among the 14 HC genes located in the region, only the gene *TrturSVE1B02G00131230* showed a significant expression in root tissues. It encodes a putative vacuolar‐type H(+)‐ATPase C3 (*VHA‐C3*) transmembrane subunit. In Arabidopsis, mutants in the homologous gene (*AT4G38920*) with reduced *VHA‐c1* gene expression showed a reduction in root growth (Padmanaban et al. 2004), whereas alfalfa overexpressing the *ScVHA‐C* gene showed increased root length compared to the wild type in salt stress conditions (Wang et al. 2016).

### 2.7 The Svevo Modern Durum Wheat Genome Is a Mosaic of Haplotypes Inherited From Wild Emmer of Both North‐Eastern and Southern‐Levant Origin

Thanks to its improved contiguity and gene space annotation, Svevo Rel.2.0 enabled haplotypes to be defined and traced back to a representative set of wild tetraploids with increased accuracy and depth, at both macro‐ and micro (gene) levels. Svevo haplotypes were traced back to a set of 12 *T. timopheevii* ssp. *araraticum* (wild) and ssp. *timopheevii* (domesticated, GGAA) accessions and 42 Wild Emmer Wheats accessions (WEW, *T. turgidum* ssp. *dicoccoides*) representative of the two main regions of origin, Israel/Southern‐Levant (SL‐WEW) and Turkey/North‐Eastern Fertile Crescent (NE‐WEW, Table S18). After mapping of exome sequencing data from these 54 ancestor accessions onto the Svevo Rel.2.0 genome assembly, 660 673 polymorphic SNPs were identified and parsed in haplotype windows of 100 non‐overlapping SNPs to paint high‐resolution chromosome maps of ancestry introgression at both long‐range and single‐gene levels (Figure S15 and Table S19). Svevo chromosome regions were claimed as ‘mainly inherited’ from one of the three main donor populations when the local Svevo haplotypes were found to trace to population‐specific wild haplotypes.

**FIGURE 5:**
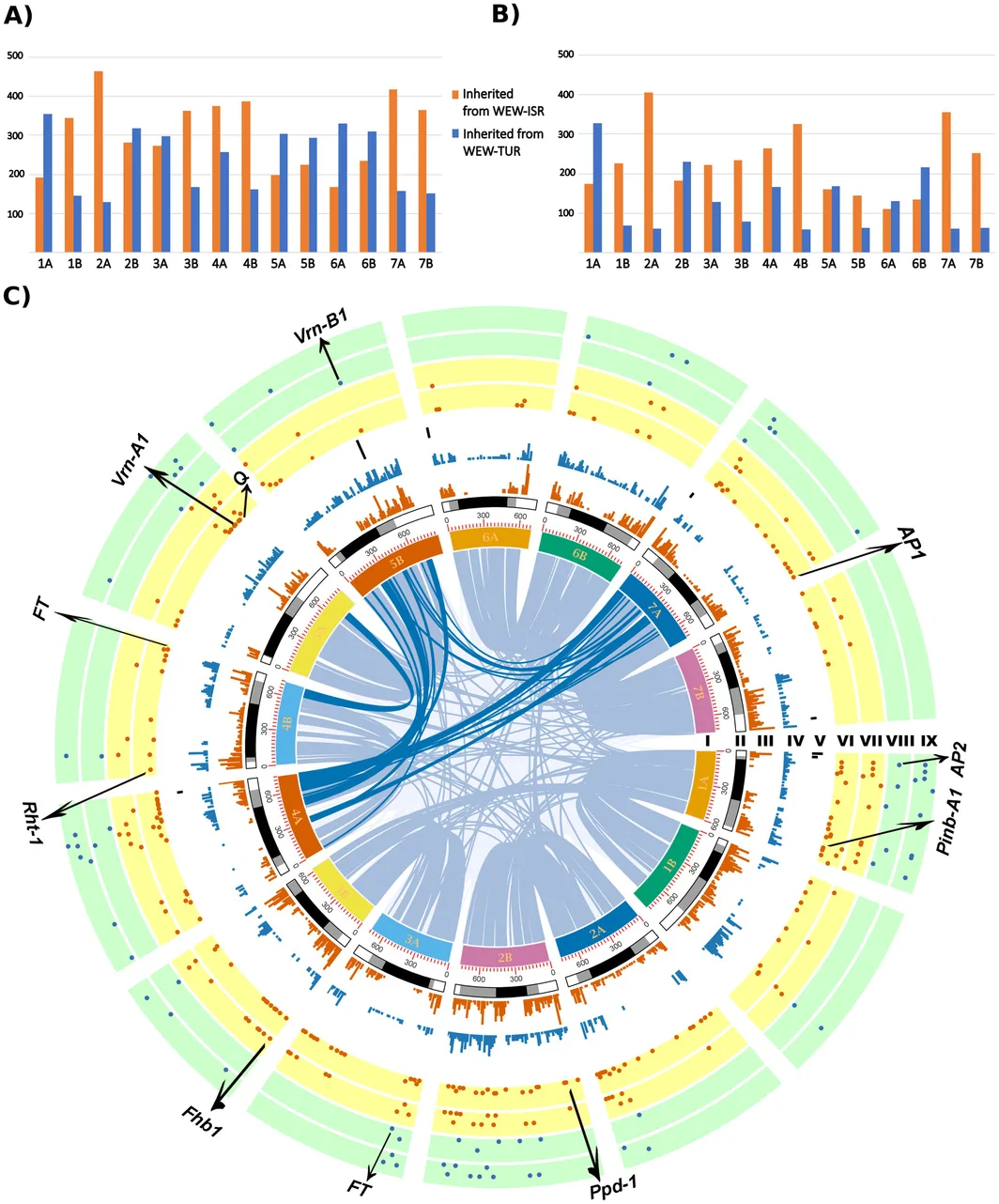
Structural and functional synteny landscape of the durum wheat genome. (A) Plots of chromosome ancestry of the *cv*. Svevo at 75% conservation threshold, with chromosome portion inherited from SL‐WEW as orange bars and from NE‐WEW as blue bars. (B) Plots of chromosome ancestry of the *cv*. Svevo at 95% conservation threshold, with chromosome portion inherited from SL‐WEW as orange bars and from NE‐WEW as blue bars. (C) Tracks from inside to outside: (I) Chromosome name and size (major tick 300 Mb and smaller tick 30 Mb); (II) Chromosome partitioning into regions: Distal (white), peri‐centromere (dark grey), and centromere (black); (III) Intervals inherited from Israel/Southern‐Levant (SL‐WEW) at 75% (orange bars); (IV) Intervals inherited from Turkey/North‐Eastern Fertile Crescent NE‐WEW at 75% (blue bars); (V) Intervals inherited from GGAA *T. timopheevii* ssp. *araraticum* and ssp. *timopheevi* at 75% (black bars); (VI) Developmental and (VII) Stress‐related genes inherited from SL‐WEW (brown dots); (VIII) Developmental and (IX) Stress‐related genes inherited from Turkey accessions (blue dots). Links in the center show synteny between the sub‐genomes: Pale‐blue indicates links between homoeologous chromosomes and dark blue indicates links between large translocated regions, confirming previous data (Maccaferri et al. 2019). Some of the important domestication genes derived from SL‐WEW and NE‐WEW have been highlighted, that is, Vernalization gene (*Vrn*); Spike architecture (*Q*); Flowering locus T‐like genes (*FT*); Reduced height genes (*Rht*); Fusarium head blight resistance genes (*Fhb*); Photoperiod‐responsive genes (*Ppd‐1*); Apetala (*AP1‐2*); Puroindoline B (*PinB*).

This analysis revealed that inheritance of the Svevo genome haplotypes was balanced from the two main WEW populations, NE and SL, with the latter showing a slight prevalence. SL‐WEW indeed contributed 41.96% (2.09 Gb) and 42.95% (2.20 Gb), respectively, to the A and B Svevo genome content at the 75th percentile conservation threshold. In contrast, WEW from Turkey/North‐Eastern Fertile Crescent contributed 36.79% (1.83 Gb) and 30.24% (1.55 Gb) to the A and B genomes, respectively. At chromosome level (Table S19, Figure 5A,B), with the 75th percentile threshold, chromosomes 1A, 5A, 5B, 6A, and 6B showed a prevalent contribution from NE‐WEW (40%–60% and 32%–27% for NE‐ and SL‐WEW, respectively), whereas an opposite ancestry transmission pattern characterizes the chromosomes 1B, 2A, 3B, 4A, 5B, 7A, and 7B (48%–58% and 16%–35% for SL‐ and NE‐WEW, respectively). Additionally, a small portion, 0.34% (17.43 Mb), was clearly contributed by *T. timopheevii* (GGAA) (Figure 5C, Figure S14). Domestication, evolution under domestication (Abbo et al. 2014), gene flow, recombination, and subsequent breeder's selection might have shaped the retention patterns: the distal regions showed conserved ancestry with SL‐WEW and NE‐WEW for 18.46% and 12.47% of the genome, respectively, whereas pericentromeric and centromeric regions harboured larger, domestication‐related linked blocks (SL‐WEW: 29.34% and 37.1%, respectively; NE‐WEW: 19.73% and 34.82%, respectively). When the transmission rate was evaluated using known genes on the basis of the 95th percentile transmission conservation threshold, functional asymmetry emerged in developmental (39.9%) and stress‐response (34.8%) genes, as well as associated with the presence of *Q* (spike architecture) and *Fhb1* (Fhb resistance), underscoring the SL‐WEW's dominant role in adaptive evolution. Instead, the NE‐WEW contribution was evinced only at lower thresholds, aligning with its diminished genomic footprint (Table S20).

As a case study, we examined in greater detail the correspondence between the outputs of the haplotype tracing pipeline and the genomic positions of genes known to be associated with the evolution under domestication. We examined the ancestral haplotype origin of a set of 34 Svevo genes, all known to be involved in the regulation of the vegetative‐to‐generative transition (developmental regulatory genes) and spikelet primordia differentiation in response to environmental factors, including the *Ppd1*, *Vrn‐1*, *ZCCT*, *PhyC*, *TaFT‐1*, *Leafy‐like, APO*, and *Constans‐1* (Fernández‐Calleja et al. 2021; Paraiso et al. 2024; Shaw et al. 2020) loci and their respective homeologs. Haplotype tracing across these genes revealed that, in 24 out of 34 loci (70.6%) examined, the Svevo haplotype could be unambiguously assigned to the SL‐WEW population, with 20 of them exhibiting complete (100%) attribution (Table S21). This pronounced bias indicates that the haplotype architecture underlying key developmental processes in modern durum wheat (Svevo) was most likely preferentially inherited from the SL‐WEW lineage to a substantially greater extent than observed at the genome‐wide level. Moreover, considering the major developmental regulators, including *Ppd‐A1*, *Ppd‐B1*, *Vrn‐A1*, *ZCCT‐A*, *PhyC‐A*, *TaFT‐A1*, and *TaFT‐B1*, the Svevo haplotype was entirely (100%) traced back to the SL ancestral source. Conversely, Svevo carried haplotypes closer to the ancestral NE‐WEW lineages at only eight genes, including *Vrn‐B1*, *Leafy‐like‐B, APO* (Paraiso et al. 2024), and *Constans‐B1*al.

## 3 Discussion

The combination of Illumina short‐reads with the DeNovoMAGIC technology (NRGene Inc.), and scaffolding supported by genetic mapping and chromosome conformation capture (Hi‐C seq), generated the first published durum wheat reference genome (Svevo Rel.1.0, Maccaferri et al. 2019). In the current study, improved long‐read sequencing technologies and assembly algorithms have enabled the generation of a novel genome assembly of the durum wheat reference genome *cv*. Svevo markedly improved in quality. By leveraging HiFi long‐read sequencing, optical mapping, Hi‐C seq information, and extensive curation, we achieved greater contiguity, accuracy, and completeness compared to the previous version Svevo Rel.1.0, revealed by a greater assembly length (10.387 Gb vs. 9.96 Gb) and contiguity (868/259 primary/hybrid scaffolds vs. 129 464 scaffolds), and fewer unanchored scaffolds length (19.8 Mb vs. 499 Mb). The new reference genome assembly of Svevo represents the gold standard for the ongoing tetraploid wheat pangenome initiative.

In Svevo Rel.2.0, we still observed significant differences in contiguity between subgenome A and B, largely driven by differences in genome architecture, particularly the extensive presence of non‐genic, long simple sequence repeats in subgenome B. Although each chromosome in the A subgenome is captured by 1 to a maximum of 14 hybrid scaffolds, subgenome B chromosomes are each captured by 28–41 hybrid scaffolds (Table S1). Reduced contiguity in the B subgenome was also reported in the PacBio HiFi‐based assembly of the common wheat cultivar Fielder, as well as in Illumina‐based assemblies included in the bread wheat pan‐genome (Walkowiak et al. 2020). It was suggested to be a result of younger repeats in the B genome (Sato et al. 2021). Besides the key role of high‐quality long‐read sequencing, the quality of the Svevo Rel.2.0 annotation was improved on the basis of a broad and well‐structured transcriptomic dataset, enabling the annotation of thousands of loci not represented in the previous annotation, with a more precise gene model prediction. The inclusion of long‐read cDNA sequencing further enhanced the accuracy of splicing and UTR definition, resolving chimeric annotations present in Rel.1.0. Overall, the quality of Svevo Rel.2.0 is in line with that of long‐range sequencing‐based assemblies generated for other wheat varieties, including CS (Zhu et al. 2021), Kariega (Athiyannan et al. 2022), Langdon (Chen, Li, et al. 2025), and Kronos (Seong et al. 2026) and is particularly outstanding for having few unanchored scaffolds (19.8 Mb) and a high gene integrity (Busco 99.94%).

Besides statistics, we provided two case studies demonstrating that the higher accuracy and integrity of Svevo Rel.2.0 assembly also led to improved gene annotation. Seed storage proteins are encoded by multigene families clustered in a few loci and characterized by repeat sequences that make it challenging to achieve a correct assembly (Wang, Li, et al. 2020; Paris et al. 2021). The quality of Svevo Rel.2.0 assembly allowed us to obtain complete full‐length sequences of all annotated prolamins, including those not previously resolved in Svevo Rel.1.0 because of the presence of repetitive regions that may have affected the correct assembly. In addition, the improved assembly of Svevo Rel.2.0 revealed a bipartite structure at the *Gli‐B2* locus on chr. 6B, and also corrected an inversion spanning a 1 Mb region on Chr 6A (Figure 2B), previously reported in hexaploid wheat (Huo, Zhang, et al. 2018; Huo, Zhu, et al. 2018; Halstead‐Nussloch et al. 2021). Among the different prolamins, LMW‐GS gives a major contribution to gluten quality in durum wheat (Ruiz and Giraldo 2021). In Svevo Rel.2.0, we identified 4 LMW‐GS genes in the B subgenome, whereas in Rel.1.0, only one pseudogene could be assigned to chr. 1B. Consistent with previous observations (Muccilli et al. 2010), a clear distinction was observed in the structure of LMW‐GS present in the A and B subgenomes. Chr. 1A encodes (in addition to a pseudogene) an i‐type subunit and a subunit in which one of the two cysteine residues predicted to be involved in interchain disulfide bonds is located close to the N‐terminus of the mature protein. Conversely, all LMW‐glutenin genes on the B subgenome have a first cysteine residue potentially available for polymer formation at position 65 or 66, in the repetitive domain (Figure S10). It will be interesting to assess how variations in cysteine location and other structural differences between LMW‐GS can modulate the impact of A and B subgenomes on the gluten network structure and dough strength.

Regarding LRR‐CRs, the A and B subgenomes contain 1.7 and 1.9 times more genes than rice, respectively, and the proportion of copies containing nonsense mutations (NsMCG) is twice as high (about 65% in Svevo vs. 30% in rice). These observations confirm the occurrence of continuously ongoing gene duplication events in the *Triticeae* lineage, but also highlight that durum wheat tolerates a larger fraction of putative non‐functional genes. The accuracy of the Svevo Rel.2.0 assembly, combined with an iterative annotation approach including manual curation (Gottin et al. 2021), allowed a comprehensive and curated annotation of LRR‐CRs, encompassing both functional genes and NsMCG, the latter potentially representing alternative alleles associated with disease susceptibility.

The contiguity and accuracy of Svevo Rel.2.0 were definitively proven by the successful physical mapping of the tough glume locus *Tg1‐B* on chr. 2B. Likely because of physical rearrangements resulting in recombination suppression, scaffolds from the *Tg1‐B* region remained unassigned in Svevo Rel.1.0, which relied on genetic data from a Svevo × Zavitan mapping population (Avni et al. 2014; Lev‐Mirom, Avni, et al. 2026). However, long‐read sequencing across the locus allowed for a correct assembly, revealing a 5 Mbp inversion associated with the free‐threshing phenotype. Inversions in various species are known to confer adaptive traits by disrupting genes, altering promoter regions, or modifying local chromatin regulation (Crow et al. 2020); here, this major structural rearrangement serves as the functional driver of the free‐threshing trait in tetraploid wheat, addressing a long‐standing gap in domestication history. The complete linkage between this inversion and the free‐threshing phenotype across 159 genotypes, including the identification of a rare progenitor haplotype primarily in Ethiopian wheat, suggests that this structural variation was a singular, pivotal evolutionary event. Our current haplotype analysis definitively traces this progenitor lineage back to Ethiopian durum genotypes, allowing us to construct a compelling narrative that links a major biotechnological leap in wheat evolution to the trade networks of the Roman Empire and the Ethiopian highlands. This finding suggests a new paradigm of wheat evolution from simple point mutations to large‐scale structural variations, providing molecular evidence for the transition from hulled emmer to the easily processed varieties that underpin modern agriculture.

To maximize the leverage of Svevo Rel.2.0 as an integrated platform for the durum wheat genomics community, we provided this reference assembly with additional tools. The transcriptional landscape of the *cv*. Svevo was comprehensively described in an RNA‐Seq‐based atlas, which can be iteratively explored through a dedicated eFP Browser, analogously to bread wheat. In addition, the catalogue of tetraploid wheat genetic loci projected on the Svevo Rel.1.0 (Maccaferri et al. 2019) was updated with more recent insights, and broadened the phenotypic traits considered, then projected on Svevo Rel.2.0. Increasing the QTLs catalogue enhanced the quality of the durum QTLome since the newly added QTLs made the QTL hotspots more robust, as sustained by a larger number of evidence from different genetic materials and/or narrowing confidence intervals, and facilitated the identification of candidate genes, possibly supported by orthologous relationship or gene expression evidence. Although gene candidates require further validation, the phenotypes of corresponding mutants or transgenics can substantiate the genetic effects of the hotspots. The integration of a highly curated genome annotation with a high‐resolution QTLome and a multi‐stress/organ expression atlas provides a powerful framework for identifying relevant genomic regions, closely linked molecular markers, and candidate genes, acting as a very useful guide in studies aimed at cloning causal genes for traits of agronomic importance. In bread wheat, QTL from interval mapping have been catalogued in QTLome and further elaborated in meta‐QTLs for many different traits, that is, FHB (Venske et al. 2019), grain yield (Saini et al. 2022), thermotolerance (Kumar et al. 2021), and quality (Quraishi et al. 2017) and then compared with available results of GWAS (Yang et al. 2021; Chen, Chen, et al. 2025) and transcriptomics (Vasistha et al. 2024) to further confirm the reliability and robustness of the QTL hotspots. The increasing and intensive cataloguing of QTLs and subsequent meta‐analysis has effectively revealed the complex genetic architecture of multigene quantitative traits in bread wheat (Sharma et al. 2023). Similarly, with durum wheat, decades of genetic mapping have yielded a wealth of data, enabling robust integration in a durum QTLome.

Since its conceptualisation (Salvi and Tuberosa 2015), the crop QTLome has been conceived as an approach to refine, summarise, and increase the accuracy of mapping QTLs and to translate this information to breeders. Major‐effect QTLs are rare in crop germplasm and have often already been exploited in breeding programs. Instead, most fundamental and complex agronomic traits are underpinned by a plethora of minor QTLs that interact strongly with the environment, which limits the practical significance of discovering individual QTLs. Although a QTLome analysis cannot increase the inherent biological effect of the individual QTLs that define complex traits, integrating these data provides a comprehensive overview of the genetic architecture of the trait. Indeed, a comprehensive QTLome streamlines the identification of consensus regions (QTL hotspots) where multiple QTLs for related traits converge. Then, the co‐localization of QTLs across multiple studies—even those with minor effects—can reveal stable and robust drivers of target traits and narrow their genomic intervals. A detailed QTLome allows breeders to prioritize major and minor QTLs with more stable effects, identify the specific germplasm required for their introgression, and elucidate the correlations and trade‐offs between different traits. Importantly, the integration of information embedded in the components of this advanced genomic toolbox presented herein has the potential to enhance and expand our understanding of wheat—both durum and bread wheat diversity and functionality, that is, toward cloning more major QTLs and unravelling the gene networks relevant for plant development and response to abiotic and biotic stresses. This includes insights into genetic diversity, the origin, and distribution of allelic variants, and the genetic architecture of key phenotypes. In the coming years, we expect that the integration between QTLomes and pangenomes will provide even more robust evidence of the role of natural variation underpinning phenotypes of interest, enabling researchers to identify and focus on genomic regions that warrant deeper investigation. As an example, we present the mapping of traceable wild emmer haplotypes onto the Svevo genome. By distinguishing between the two primary wild emmer populations—those from the North‐Eastern Fertile Crescent and the Southern‐Levant—we revealed that the modern Svevo genome exhibits a complex mosaic structure, incorporating haplotypes from both ancestral sources in different regions of the genome. Similar complex haplotype patterns, indicative of extensive gene flow among multiple wild populations, agreed with what has already been observed previously on the basis of molecular markers, though at a much lower level of resolution (Luo et al. 2007; Civan et al. 2013). Compared to previous studies (Ozkan et al. 2002), we detected a higher –than‐ expected overall contribution of the Southern‐Levant WEW haplotypes to the Svevo genome, a finding that deserves deeper investigations. When considering a set of known regulators highly relevant for vegetative‐to‐generative switch (e.g., phenology) and therefore adaptation, evolutionary dynamics, and breeding, the contribution of Southern‐Levant ancestral wild emmer haplotypes was beyond our expectation (70.58% total contribution as compared to 41.96% observed genome‐wide), potentially indicating Southern‐Levant Fertile Crescent as the site contributing the ancestral diversity most relevant for shaping modern durum wheat. Linking these findings with gene annotation underscores the potential significance of specific chromosome regions, genes, and gene families in shaping the evolutionary trajectory of tetraploid wheat.

## Author Contributions

Project establishment and coordination: R.T., M.Ma., C.J.P., and L.C. Genome sequencing and assembly: G.Z.‐H., V.L., K.F., and C.H. Manual curation of the assembly and centromere determination: E.M., C.F., M.B., H.T., P.N., H.S., V.K.,S.W., J.E., and H.S.C. RNAseq data were contributed by: J.D.F., S.S.X., J.I.S., A.S., N.P., F.M.B., M.S.‐G., G.S., P.L.C., G.G., G.A., A.G., I.M., S.M., F.S., S.P., and M.Mo. Genome annotation: D.S., P.F., M.G., G.K., R.L.R.‐P., M.S., H.G., K.F.X.M., A.H., C.F., and B.L. Manual curation of prolamin genes: M.L., A.C., R.P., and E.T. Manual curation of LRR‐CR genes: V.R., J.G., and N.C. Genome browser: V.C.B, E.Y., and T.Z.S. Electronic Fluorescent Pictographic (eFP) Browser: A.P. and N.J.P. Physical map of *Tg1‐B* locus and *Tg1‐B* haplotype distribution in tetraploid germplasm: Y.L.‐M., F.D., L.O., T.E., C.L., S.S., M.Ma., and A.D. Haplotype‐based ancestry analysis: E.C.‐G., M.Ma., C.L., M.A.F., S.S., and S.G.K. QTLome projection E.M., A.M.M., C.F., D.M., F.D., A.P.V., C.L., S.S., C.C. E.M., and L.C. drafted the manuscript with input from all authors. All co‐authors contributed to and edited the final version. All authors read and approved.

## Funding

This work was supported by European Union Next‐Generation‐EU, Investimento 1.4‐D.D. (1032 17/06/2022, CN00000022). Joint FACCE‐JPI Suscrop 2022, WheatSecurity DM.142692. Biotechnology and Biological Sciences Research Council, Decoding Biodiversity BBX011089/1. Agence Nationale de la Recherche, ANR‐24‐CE20‐2538. HORIZON EUROPE Framework Programme, PRO‐WILD GA number: 101134965 and PRO‐GRACE GA number: 101094738. German Federal Ministry of Education and Research, 031B0190 (de.NBI). Italian Ministry of University and Research PRIN 2020, PanWheatGrain (Grant 20209XY5R9). 4D Wheat project supported by Genome Canada, Genome Prairie, the Saskatchewan Ministry of Agriculture, Saskatchewan Wheat Development Commission, Alberta Grains, Secan, Agriculture and Agri‐Food Canada and the Manitoba Crop Alliance. Arab Fund for Economic and Social Development, Modernization of Crop Breeding Programs in Arab Countries. Natural Sciences and Engineering Research Council of Canada, Discovery Grant.

## Materials and Methods

Given to the limited space available all Materials and Methods and some Supplementary Results have been moved to Supporting information.

## Conflicts of Interest

The authors declare no conflicts of interest.

## Supporting information


**Figure S1:** Hi‐C contact map comparison between Svevo Rel2.0 and Rel1.0 chromosome assemblies. The heatmaps represent the pairwise intra‐chromosomal (A) and inter‐chromosomal (B) contact frequencies of the Hi‐C reads mapped to the Svevo Rel.2.0 and Rel.1.0 assemblies. The intensity of the colour corresponds to the interaction frequency.
**Figure S2:** Prediction of the centromeric domain in chr. 5B from Hi‐C data.
The genomic interval corresponding to the putative centromeric domain was identified as a low‐interacting region at the intersection of the diagonal and the antidiagonal in the Hi‐C contact probability heat map. The diagonal‐antidiagonal cross pattern, visible in the inset showing the whole 5B chromosome, reflects Rabl organization of Svevo chromosomes.
**Figure S3:** Major rearrangements detected between reference durum, wild emmer and bread wheat genome assemblies. The following assemblies were compared: Svevo Rel.2.0 and Svevo Rel.1.0 (Maccaferri et al. 2019), or Zavitan WEWSeq v2.1 (Zhu et al. 2019), or Chinese Spring IWGSC RefSeq v2.1 (Zhu et al. 2021). The plots identify sequence collinearity across the diagonal with blue dots, and inversions (+/− alignment orientations) between the two genomes as red dots. Duplicated genome segments are indicated by red or blue dots positioned away from the diagonal.
**Figure S4:** Sub‐genome distribution of the main TE classes in Svevo Rel2.0 durum wheat genome wheat. A table and a plot provide a summary of the relative TE distribution between the A and B sub‐genomes of Svevo durum wheat. TE acronyms are as follows: RLC (T*y1/copia* LTR–RT), RLG (*Ty3/gypsy* LTR–RT), RLX (unclassified LTR‐RT), RIX (LINE non‐LTR RT), RSX (SINE non‐LTR RT), RXX (unclassified RT), DTX (unclassified DNA transposon), DTA (*hAT* DNA transposon), DTC (*CACTA* DNA transposon), DTM (*Mutator* DNA transposon), DTT (*Tc1–Mariner* DNA transposon), DXX (MITE DNA transposon), and DTH (PIF– Harbinger DNA transposon).
**Figure S5:** Heatmap showing the genomic distribution of 13 transposable element (TE) families across chromosomes 1A and 1B of durum wheat ( *Triticum turgidum ssp. durum* ). TE densities were calculated as the fraction of bases covered within non‐overlapping 1 Mb windows. Colour intensity reflects the local density of each TE family, with independent colour scales adjusted to highlight both abundant (RLC, RLX, RLG; maximum fraction set to 0.6) and less abundant families (all others; maximum fraction set to 0.1). Vertical dashed red lines indicate the approximate position of the centromeres, marked with an asterisk. White vertical separators distinguish adjacent chromosomes.
**Figure S6:** Sub‐genome distribution of annotated genes in Svevo Rel.2.0. Table (A) and plot (B) provide a summary of the annotated gene distribution between the A and B sub‐genomes of Svevo durum wheat.
**Figure S7:** Comparison of gene models across Rel2.0 and Rel1.0 genome annotations and corresponding gene expression profiles. Gene structures for selected loci are shown for comparison between Svevo Rel2.0 and Rel1.0 genome annotations in panels A–D. For each locus, exon–intron structures of all annotated transcript isoforms are displayed for the corresponding annotations (left panels). Exons are represented as boxes and introns as connecting lines, with different colours indicating distinct genes within each annotation. Transcript identifiers are shown next to each isoform. Genomic coordinates correspond to the same reference genome assembly. The right panels show the screenshots from the Durum Wheat eFP Browser with the expression profiles for the genes represented in each locus. (A) TrturSVE3A02G00657080, encoding a dehydration‐responsive element‐binding (DREB) transcription factor, expressed in the late stages of grain filling and embryo maturation and dehydration. In Rel1.0, this locus was chimerically annotated, fused with the flanking locus. (B) TrturSVE3B02G00918510, encoding Gibberellin 20‐oxidase 2, highly expressed in developing inflorescence primordia and previously annotated as a low‐confidence gene in Rel1.0. (C) TrturSVE3B02G00798860–TrturSVE3B02G00798880, encoding class I heat shock proteins, induced by heat stress in both leaves and roots. The three loci were annotated as a single chimeric locus in Rel1.0. (D) TrturSVE5A02G01397160, corresponding to the VRN2/ZCCT‐A2 CCT domain‐containing protein locus. This copy of the two tandemly duplicated floral repressors ZCCT1 and ZCCT2 that made the VRN2 locus was not annotated in the Rel1.0 genome.
**Figure S8:** OMArk analysis (clade pooideae, v0.3) comparing the annotation of Svevo Rel.1.0 and Rel.2.0. OMArk analysis was performed to compare the completeness and annotation quality between the Rel.1.0 and Rel.2.0 annotations of cv. Svevo. The analysis assesses differences in the proportion of missing and unknown genes for all genes, high confidence (HC) plus low confidence (LC) genes, or only HC genes. HOGs (Hierarchical Orthologous Groups) represent sets of genes from different species that descend from a single gene in the last common ancestor and serve as the reference framework for assessing annotation quality and completeness.
**Figure S9:** Screenshots from the Durum Wheat eFP Browser. Pictograms showing different wheat developmental stages and tissues included in the development (A), nutrient and abiotic stress map (B) and abiotic stress view. False colour highlights the tissue/organ included in the atlas.
**Figure S10:** Protein sequence alignment of LMW‐GS from the Svevo genome. Cysteine residues predicted to be implicated in intrachain and interchain disulfide bonds are indicated by green and red boxes, respectively.
**Figure S11:** Schematic representation of the *Tg1‐B* locus on the short arm of chr. 2B, with a representation of the Svevo × Zavitan genetic map in the corresponding region. Genetic markers are aligned with their physical positions on chr. 2B in both the Zavitan WEWSeq v2.1 and the Svevo Rel.2.0 genome assemblies. Break points defining the 5 Mb inversion are indicated with red lines, with the physical positions on the two genome assemblies.
**Figure S12:** The QTL hotspot TKW_12. (A) QTL clustered by the hotspot TKW_12 are reported as coloured bars, sized on the basis of the Confidence Interval of each QTL, on the long arm of the chr. 4A. (B) The eight sucrose:sucrose 1‐fructosyltransferase coding genes included in the minimal interval region are listed, together with their position and manually curated annotation. (C) The gene expression pattern in the different tissues across the plant growth cycle is shown as absolute expression level (TPM).
**Figure S13:** The QTL hotspot Root_25. (A) QTL clustered by the hotspot Root_25 are reported as coloured bars, sized on the basis of the IC range of each QTL, on the long arm of the chr. 4B. (B) The list of genes encompassed by the minimum interval region included *TrturSVE4B02G01227910*, homologous to *TaMORb*, whose expression pattern across tissues of durum wheat genes is represented by the pictogram derived from the Durum Wheat eFP Browser.
**Figure S14:** The homeologous QTL hotspots Root_38 and Root_42. QTL clustered by the hotspot Root_38 and Root_42 are reported as coloured bars (blue for QTL from association mapping and green from linkage mapping), sized on the basis of the Confidence Interval range of each QTL, on segments of the chr. 6A and 6B, respectively, in (A) and (C). The pictogram, derived from the Durum Wheat eFP browser, shows the expression of the homologous candidate genes (*TrturSVE6A02G01686810* (B), *TrturSVE6B02G01842660* (D)) retrieved within the minimum interval region of each hotspot.
**Figure S15:** Svevo haplotype composition. The Svevo genome was divided into windows comprising 100 SNPs each. The windows were painted on the basis of the percentage of the composition of the SNP‐based clustering per window. Blue colour indicates origin from wild emmer accessions collected in Turkey, and yellow colour indicates origin from wild emmer collected in Israel‐Southern Levant. Intermediate colour tones indicate situations in which it is not possible to determine the origin. Some windows in the Svevo genome originate from the *T. timopheevii* wheat lineage (AAGG gene pool) and were labelled with purple colour. The white line indicates the robustness of the assignment of each window on the basis of the number of wild emmer accessions supporting the assignment to a particular subpopulation, whereas the black line indicates the minimal summed SNP score.
**Figure S16:** Alignment of amplicons to display insertions/deletions causing size variations among amplicons obtained by assessing diversity at the locus chr. 2B *Tg1‐B* in the 285 diverse panel. Alignment of the amplicon sequence mostly retrieved in hulled accessions (H1, 2, 3), positions are related to Zavitan WEWSeq 2.0 (more detailed in Table S12c).
**Figure S17:** The QTL hotspot Disesase_14. (A) QTL clustered by the hotspot Disease_14 are reported as coloured bars, sized on the basis of the IC range of each QTL, on the long arm of the chr. 4A; (B) the list of genes encompassed by the minimum interval region (both HC and LC), including a few non‐functional LRR‐CR genes; (C) the pictogram of the candidate gene *TrturSVE4A02G00978380*, derived from the Durum Wheat eFP browser.

**Table S1a:** Features of the Svevo Rel.2.0 genome assembly: general statistics by subgenome.
**Table S1b:** Features of the Svevo Rel.2.0 genome assembly: assembly length and N° of scaffolds for each chromosome.
**Table S1c:** Features of the Svevo Rel.2.0 genome assembly: comparison to Svevo Rel.1.0 (Full assembly).

**Table S2:** Centromere positions determined from Hi‐C data.

**Table S3:** Transposon composition of Svevo Rel. 2.0 reference genome determined by homology to a Triticeae repeat library.

**Table S4:** List of RNA sample pools sequenced with long read Oxford Nanopore Technologies (ONT) cDNA sequencing. The different combinations of tissues/organs, developmental stages and growth treatments that made.

**Table S5:** Biotype and gene statistic of annotation for Svevo Rel.2.0.

**Table S6:** Summary of the assessment of assembly and annotation on the basis of 4896 Busco genes on the new assembly Svevo Rel.2.0 and on the previous Svevo Rel.1.0.

**Table S7:** Summary of Svevo Rel2.0 and Rel1.0 annotation assessment through the evaluation of two dataset of experimentally validated genes.

**Table S8:** List of prolamin genes identified and manually curated in durum wheat Svevo Rel.2.0.

**Table S9:** Number of genes encoding LRR‐CRs per chromosome and family.

**Table S10:** Correspondence between LRR‐CR annotation and Svevo Rel.2.0 High Confidence (HC) and Low Confidence (LC) gene prediction IDs.

**Table S11:** Statistics on predicted protein composition related to LRR‐CR in the old and in the newly Svevo genome assembly.

**Table S12:** Passport data and haplotype assignment for 285 tetraploid genotypes belonging to different *T. turgidum* subspecies (DW: Durum Wheat cv and landraces ( *T. turgidum sspdurum* ); DEW: Domesticated Emmer Wheat ( *T. turgidum ssp. dicoccum* )).

**Table S13:** Passport data and haplotype assignment for 159 tetraploid genotypes (DW: Durum Wheat cv and landraces ( *T. durum* ); DEW: Domesticated Emmer Wheat ( *T. tirgidum ssp. dicoccum* ); WEW: Wild Emmer Wheat ( *T. turgidum ssp. dicoccoides* )).

**Table S14:** List of published QTL and MTA (as on December 31th 2024), considering both linkage and association mapping studies conducted in tetraploid wheat population/germplasm collections, and anchored on the Svevo.

**Table S15:** List of the 26 QTL hotspots clustering QTL for TKW.

**Table S16:** Absolute expression level of the genes annotated within the minimum overlapping region of 4 QTL hotspots (Disease_1, Disease_6, Disease_13, Disease_20, and Disease_22).

**Table S17:** Gene models from LRR‐CR annotation with correspondant gene IDs from the automatic annotation for the seven QTL hotspots (1, 6, 7, 13, 20, 22, and 40) related to Fusarium resistance (including at least two QTLs).

**Table S18:** Passport data of the 42 wild emmer wheat and 12 AAGG accessions used for assessment of ancestry of Svevo's haplotypes.

**Table S19:** Summary statistics of genome ancestry of durum wheat genome.

**Table S20:** Genomic ancestry patterns and selection signatures in tetraploid wheat on the basis of haplotype transmission.

**Table S21:** Genomic ancestry patterns and selection signatures of genes controlling vegetative‐to‐generative transition in tetraploid wheat on the basis of haplotype transmission.

**Table S22:** List of public RNA‐Seq datasets (Maccaferri et al. 2019). (https://trace.ncbi.nlm.nih.gov/Traces/?view=study&acc=SRP149116).

**Table S23:** Statistics of RNA‐Seq Illumina reads assembly produced for each tissue through StringTie2 v2.1.5, for both public data (Table S22) and newly generated short reads RNA‐seq experiments (the 3 replicates of each tissue/stage combinations) were combined.

**Table S24:** Statistics of RNA‐Seq Illumina reads assembly produced for each tissue through Scallop v0.10.5, for both public data (Table S22) and newly generated short reads RNA‐seq experiments.

**Table S25:** Statistics of ONT cDNA assembly produced for tissue pools through StringTie2 v2.1.5 for long read ONT cDNA sequencing (sample code detailed in Table S5b).

**Table S26:** Statistics of the REAT transcriptome runs: Mikado was run with all RNA‐Seq Scallop and StringTie2 assemblies, PacBio IsoSeq alignments and Nanopore assembled transcripts plus an additional run with only the long‐reads.

**Table S27:** NCBI references to Protein sequence datasets from 10 Poaceae species.

**Table S28:** Basic statistics of the gene models obtained after consolidation of cross species protein alignments (miniprot v0.3 and spaln v2.4.7) with the REAT Homology workflow.

**Table S29:** Basic statistics of the alternative predictions of protein coding genes on the basis of the repeat annotation, RNA‐Seq mappings, transcript assemblies and alignment of protein sequences via the REAT prediction workflow.

**Table S30:** Prolamin expression.

**Data S1–S9:** pbi70673‐sup‐0032‐Supinfo.zip.

## Data Availability

The data are freely available and publicly accessible through deposition on public databases. Genome assembly and sequencing raw data and Bionano Optical maps were deposited on European Nucleotide Archive—ENA under the Project number ERP171040. The annotation datasets are available through a Data Download site in the U.S. Department of Agriculture's GrainGenes database [REF: PMID: 35616118] at https://graingenes.org/GG3/genome_browser // https://graingenes.org/GG3/svevo_v2. The Data Download page in GrainGenes also serves as a landing page for access to genome browsers, tools, and BLAST services (Altschul et al. 1990). Expression data are deposited under GEO accessions: GSE318160, GSE318338, and GSE318339. The electronic Fluorescent Pictographic Browser dedicated to durum wheat (Durum Wheat eFP Browser) can be accessed through this link: https://bar.utoronto.ca/efp_durum_wheat/cgi‐bin/efpWeb.cgi.
