# Supplementary material for: Durum Wheat cv. Svevo Reference Genome Rel.2.0: A Comprehensive Tool for Wheat Genomics
Source: Plant Biotechnol J. 2026 Jul 13:10.1111/pbi.70673. Online ahead of print. doi: 10.1111/pbi.70673 (PMC13398682; doi:10.1111/pbi.70673)
Supplement: Supplementary file 1 — Figure S1: Hi‐C contact map comparison between Svevo Rel2.0 and Rel1.0 chromosome assemblies. The heatmaps represent the pairwise intra‐chromosomal (A) and inter‐chromosomal (B) contact frequencies of the Hi‐C reads mapped to the Svevo Rel.2.0 and Rel.1.0 assemblies. The intensity of the colour corresponds to the interaction frequency. Figure S2: Prediction of the centromeric domain in chr. 5B from Hi‐C data. The genomic interval corresponding to the putative centromeric domain was identified as a low‐interacting region at the intersection of the diagonal and the antidiagonal in the Hi‐C contact probability heat map. The diagonal‐antidiagonal cross pattern, visible in the inset showing the whole 5B chromosome, reflects Rabl organization of Svevo chromosomes. Figure S3: Major rearrangements detected between reference durum, wild emmer and bread wheat genome assemblies. The following assemblies were compared: Svevo Rel.2.0 and Svevo Rel.1.0 (Maccaferri et al. 2019), or Zavitan WEWSeq v2.1 (Zhu et al. 2019), or Chinese Spring IWGSC RefSeq v2.1 (Zhu et al. 2021). The plots identify sequence collinearity across the diagonal with blue dots, and inversions (+/− alignment orientations) between the two genomes as red dots. Duplicated genome segments are indicated by red or blue dots positioned away from the diagonal. Figure S4: Sub‐genome distribution of the main TE classes in Svevo Rel2.0 durum wheat genome wheat. A table and a plot provide a summary of the relative TE distribution between the A and B sub‐genomes of Svevo durum wheat. TE acronyms are as follows: RLC (Ty1/copia LTR–RT), RLG (Ty3/gypsy LTR–RT), RLX (unclassified LTR‐RT), RIX (LINE non‐LTR RT), RSX (SINE non‐LTR RT), RXX (unclassified RT), DTX (unclassified DNA transposon), DTA (hAT DNA transposon), DTC (CACTA DNA transposon), DTM (Mutator DNA transposon), DTT (Tc1–Mariner DNA transposon), DXX (MITE DNA transposon), and DTH (PIF– Harbinger DNA transposon). Figure S5: Heatmap showing the genomic distribution of [file PBI-9999-0-s016.docx]

**Supporting information for “Durum wheat cv. Svevo reference genome Rel.2.0: a comprehensive tool for wheat genomics”**

**Experimental procedures**

**Genetic materials**

The durum wheat (*Triticum turgidum L.* ssp*. durum*) *cv.* Svevo, a high-quality Italian variety released in 1996, has been obtained from the cross between a CIMMYT line (pedigree rok/fg//stil/3/dur1/4/sapi/teal//hui), related to the widely utilized Yavaros79 genetic background (Jori/Anhinga//Flamingo), and the cv. Zenit originating from a cross between Italian and American cultivars (Valriccardo/Vic). Svevo pure seeds, maintained at the Department of Agricultural and Food Sciences, University of Bologna (Italy), were used for High Molecular Weight (HMW) DNA extraction and transcriptomic analyses.

The analysis of haplotype diversity at the *Tg1-B* locus was carried out using two germplasm panels. A set of 285 tetraploid wheat genotypes selected from the Global Tetraploid wheat Collection (Maccaferri et al. 2019) and from the Global Durum Panel (Mazzucotelli et al. 2020) was made of 185 durum modern/landraces (ssp. *durum*), 30 primitive tetraploid wheat accessions (ssp. *polonicum, turgidum, carthlicum, turanicum, aethiopicum*), 45 domesticated (ssp. *dicoccum*) and 25 wild emmer (ssp. *dicoccoides*) genotypes (Table S12). A second set of 159 accessions described by Lev-Mirom et al. 2026) comprised 64 wild emmer wheat (*T. turgidum* ssp. *dicoccoides*), 44 domesticated emmer wheat (*T. turgidum ssp. dicoccum*) 44 durum wheat landraces (*T. turgidum* ssp. *durum*), and 5 durum wheat cultivars (Table S13).

A panel of 42 wild emmer wheat (ssp. *dicoccoides*) and 12 GGAA *T. timopheevii* ssp. *araraticum* and ssp*. timopheevii* accessions, derived from the previously analysed exome capture sequencing dataset of 287 tetraploid wheat accession (Dia Sow et al. 2025) was used for the haplotype heritage analysis (Table S18).

al.

**DNA extraction, Bionano Optical mapping and PacBio HiFi sequencing of libraries**

Ultra-HMW DNA was isolated from young leaf tissue using the method described in Fornasiero et al. (2024). Optical genome mapping was conducted as described by Hufford et al (2021), using the Bionano Direct Label and Stain chemistry (DLS) and Saphyr analyzers (Bionano Genomics; San Diego, CA). Molecules were imaged, digitized and filtered to a minimum length of 310 kb. The filtered dataset, consisting of 4,759,282 molecules with molecule N50 of 430 kb and combined length of 2.1 Tb, was assembled into 547 maps using the Bionano Genomics software Tools version 1.6.1. The selected assembly parameters were non-haplotype, no ES, no CMPR Cut. The final Bionano assembly was 10,462,420 Mb and consisted of 547 maps with a N50 of 67,373 Mb and a maximum map size of 290,967 Mb.

Prior to PacBio HiFi library construction, the highly viscous uHMW DNA was sheared to a center of mass of approximately 40 kbp by passing multiple times through a 26G Hamilton needle. The resulting sample exhibited no noticeable viscosity and was further sheared using a Megaruptor 3 (Diagenode) system into a narrow target range peaking between 10 and 20 kbp. Library construction followed the standard protocol for the SMRTbell Express Template Prep Kit 2.0 (Pacific Biosciences) with a few modifications. Upon removing fragments under 15 kb size using Pippin HT, fragments were sequenced on Pacific Biosciences Sequel IIe analyzers. PacBio HiFi long reads were generated from 13 SMRT cells yielding 32x coverage (read N50 = 17.9 kb) after applying minimum length (5 kb) and maximum length (30 kb) filtering.

**Genome assembly and pseudomolecule creation**

HiFi reads were assembled with hifiasm v0.16.1 (Cheng et al. 2021) into 868 primary contigs (contig N50 = 30.8 Mb, sum = 10,428 Gb) using the -l0 parameter for inbred/homozygous genomes. Contigs were filtered for a minimum length of 70 kb and minimum coverage of 15x (as reported by the asm.bp.p_ctg.noseq.gfa output file from hifiasm), resulting in 813 sequence contigs. Hybrid scaffolds were created from contig sequences and genome maps using the Bionano Access platform (Tools Version 1.6.1) and the DLE-1 configuration file hybridScaffold_DLE1_config.xml using auto-conflict resolution. The resulting 263 hybrid scaffolds (scaffold N50= 112.3 Mb), as well as the un-scaffolded contigs were aligned to the iwgcs-refseqv2.1 reference genome to build tentative pseudomolecules via homology-based scaffolding. The alignment was performed using a rapid chunking method where the assembly was broken down into 100 bp “reads” per scaffold/contig that were aligned to the reference using minimap2 v2.22 (Li, 2018) and the resulting chromosome location, coordinates and orientation were determined from the “read cloud”. The final placement of scaffolds and contigs was refined with Hi-C data previously generated for Svevo Rel.1.0 assembly (Maccaferri et al. 2019, TCC22 and TCC23 samples in the European Nucleotide Archive (ENA) project PRJEB22687, <https://www.ebi.ac.uk/ena/browser/view/PRJEB22687>) using Juicer/3D DNA tools (Dudchenko et al. 2017) to create de novo pseudomolecules. A 100 bp N-gap was inserted between scaffolds. Leftover un-placed scaffolds/contigs were added as 45 separate contigs into the final Svevo Rel.2.0 assembly.

**Generation of inter and intra-chromosomal Hi-C maps**

For generating the high-resolution inter and intra-chromosomal Hi-C contact maps, the Hi-C reads (PRJEB22687) were re-mapped to the final, post-curated Rel.2.0 and Rel.1.0 assemblies using bwa-mem (Li, 2013) and the name-sorted BAM files were processed using pairtools v1.1.3 (parse, sort, dedup and select – Open2C et al, 2024) for the identification of uniquely mapped contact pairs with MAPQ ≥ 30. Contact matrices were generated using cooler v0.10.4 – Abdennur and Mirny, 2020). Multi-resolution contact matrices were generated using cooler zoomify, then normalized using iterative correction and eigenvector decomposition (ICE) as implemented in cooler balance. Balanced genome-wide inter-chromosomal maps at 500 kb resolution were visualized using HiGlass (Docker-based deployment - Kerpedjiev et al. 2018). Intra-chromosomal matrices for each individual chromosome were extracted from the genome-wide 10 kb matrix using the cooler Python API. Single-chromosome .cool files were generated and visualized with HiGlass.

**Determination of centromere positions**

In large plant genomes with *Rabl* configuration, the position of functional centromeres in chromosomal pseudomolecules can be reliably determined from Hi-C matrices, which exhibit a characteristic cross pattern consisting of a strong main diagonal and a weaker anti-diagonal with the centromere located at their intersection. These midpoints also coincide with spots of high imbalance in the number of upstream and downstream contacts (Navrátilová et al. 2022). Here, we employed this approach to identify functional centromeres in the Svevo Rel.2.0 genome. Raw Hi-C data from PRJEB22687 (Maccaferri et al. 2019) were processed by HiC-Pro v3.0.0 pipeline. Utility hicpro2juicebox.sh and Juicer tools v1.9.9 (Robinson et al. 2018) were then applied to generate Hi-C contact matrices for all Svevo chromosomes and visualize them. Genomic intervals corresponding to putative centromeric domains were identified as low-interacting regions at intersections of the diagonals and antidiagonals of the individual chromosomes (Figure S2, Table S2). To assess the completeness of the centromeric sequences, we compared the predicted centromeric intervals with the coordinates of the PacBio contigs. In four chromosomes, the centromeric interval overlapped with a sequence gap, suggesting incompleteness of the centromeric sequence. The remaining ten centromeres resulted complete (Table S2).

**Genome wide comparisons**

The Svevo Rel. 2.0 assembly was compared, at the chromosome level, with Svevo Rel.1.0, Chinese Spring v2.1 (Zhu et al. 2021) and Zavitan WEWSeq v2.1 (Zhu et al. 2019). Pairwise chromosome alignments were conducted using the nucmer command from MUMmer4 tool, version 3.23 (Marçais et al. 2018). Alignments were performed for each chromosome using Svevo Rel.2.0 as the query and other genomes as references, with the --mum options to retain only unique matches between reference and query. The output delta file was filtered using the delta-filter command with the following options: -l 10000 to keep alignments longer than 10kb between reference and query and -r and -q, which retain only long and consistent alignments for reference and query respectively. The filtered.delta files for each chromosome were used to produce alignment dotplots using R packages ggplot2 (Wickham, 2016), GenomicRanges (Lawrence et al. 2013) and dplyr (Mailund, 2019).

**Plant treatments, RNA extractions and sequencing**

In this study, 174 RNA samples representative of: i) a developmental time-course across 30 tissues x timepoint combinations, ii) 20 combinations of abiotic/nutritional stress and iii) Fusarium Head Blight (FHB) infection profiling, were produced (Table S4) and sequenced using both long and short read platforms.

For the transcriptomic atlas of the durum wheat plant development**,** durum wheat seeds *cv*. Svevo were pre-germinated in Petri dishes with wet filter paper, at 4 °C for 5 days to obtain a synchronous germination. Germinating seeds were then transferred into pots filled with loam-based potting soil, plus 1/3 of sand and perlite (in 1:1 volume ratio) in a controlled environment starting with a 14/10 hours day/night length at 14/8 °C and progressively reaching 16/8 hours day night length at 24/20 °C. Three biological replicates, consisting of at least five individual plants each, were sampled at multiple developmental stages and from different tissues through the growth cycle, as detailed in Table S4, for a total of 90 samples. All tissues were harvested between 11 AM and 1 PM and immediately frozen in liquid nitrogen upon collection. Seedling shoot and root samples were collected from wheat plants germinated and vertically grown on filter paper in rhizoboxes. Total RNA was extracted from 100 mg fresh weight material; the Direct-Zol RNA Miniprep Kits (Zymo Research) was used for leaf, root, meristem and ovary samples, while the RNeasy Plant Mini Kit (Qiagen) was used for seed samples. In both protocols genomic DNA contamination was removed using the on-column DNase treatment.

To reveal the transcriptional response to abiotic stresses, pre-germinated Svevo seeds were transferred into pots filled with a mixture of vermiculite and sand (4:1 ratio), placed in a growth chamber set at 14/10 hours day/night length at 14/8 °C for 10 days. Temperatures were then changed to 23/18°C and plants were watered with half strength Hoagland solution until plants reached the third leaf stage. The following growth conditions were then applied (Table S4): i) heat stress [HS; 37/30 °C], ii) simulated drought/osmotic [DS; half strength Hoagland plus 300 mM Mannitol], iii) salinity [SS; half strength Hoagland plus 150 mM NaCl) and iv) nitrogen application [half strength Hoagland plus 3 mM NH_4_NO_3_]. Root and shoot samples were collected from treated and control plants at 4 and 24 hafter application of the treatment, in three biological replicates, each consisting of at least five individual plants, and immediately frozen in liquid nitrogen. Total RNA was extracted from 100 mg frozen material using NucleoSpin RNA Plant, Mini kit (Macherey-Nagel) including the on-column DNase treatment for genomic DNA removal.

For profiling the early transcriptional response of Svevo spikes to FHB infection, the *Fusarium graminearum* strain CS3005 was cultured at 22 °C on half-strength V8 juice (Campbell’s) Bacto agar (BD Difco; 15 g L^-1^) in 9 cm Petri dishes. Conidial inoculum of CS3005 strain was obtained as described in Beccaria et al. 2017 and adjusted to 5 × 10^5^ conidia mL^−1^ in deionized sterile water. Durum wheat plants, *cv.* Svevo, were grown as described for the plant development time-course. Point inoculation was performed at early-anthesis (Zadoks growth stage 61): two florets of the two central spikelets in the main tiller were inoculated with 10 μL of inoculum. Mock plants were inoculated with 10 μL of sterilized water. Inoculated spikelets were marked with a marker pen to facilitate the sampling. Inoculated and mock spikes were covered with plastic bags to maintain high humidity and promote disease development. Inoculated/mock spikelets and the three flanking spike internodes were sampled 2 and 4 days after inoculation from at least 4 spikes per replicate and immediately frozen in liquid nitrogen (Table S4). Total RNA was extracted from 100 mg frozen material using RNeasy Plant Mini Kit (Qiagen) with the optional on-column DNase treatment for genomic DNA removal.

Total RNA (with ribosomal RNA depletion) Illumina sequencing of the 174 samples was conducted on NovaSeq platform with a 2 x 150 bp paired-end strand-specific protocol, generating about 30-40 M of read pairs per sample (Table S4a; raw data is deposited in Gene Expression Omnibus (GEO) database). The long-read Oxford Nanopore Technologies (ONT) cDNA sequencing was conducted on 36 pools made by combining similar tissues/organs/developmental stages or treatments (Table S4b). cDNAs prepared using a strand-switching method and the PCR-cDNA Barcoding Kit (SQK-PCB111-24 with 18 barcodes) were sequenced on a PromethION 24 instrument generating 1.7 to 6 million of high-quality reads (Q >10) per pooled sample, with a N50 around 1 kb (roughly corresponding to 1 - 6 Gb of output per pooled sample).

**Genome annotation**

*Transposon Annotation by similarity to a TE Library*

Transposons (TEs) were identified and classified through similarity searches against the *Triticeae*-specific section of the REdat_9.7 PGSB transposon library (Spannagl et al. 2016), using an approach previously applied to annotate TEs in wheat pan-genomes (Walkowiak et al. 2020). For efficient matching in this large, highly repetitive genome, we utilized the program vmatch (<http://www.vmatch.de/>). Parameters for vmatch were set as follows: identity ≥ 70%, minimum hit length 75 bp, and seed length 12 bp (command line: -d -p -l 75 -identity 70 -seedlength 12 -exdrop 5). To ensure non-overlapping annotations, we filtered the vmatch output using a priority-based approach. Matches with higher scores were prioritized and retained, while overlapping, lower-scoring hits were either trimmed or discarded. Specifically, hits were removed if they overlapped ≥90% with a higher-scoring match, or if fewer than 50 bp remained after trimming. The resulting overlap-free transposon annotation identified 8.7 Gb as TE derived corresponding to 83.9 % of the Svevo Rel.2.0 assembly.

*Reference guided transcriptome reconstruction*

Gene models were derived from Svevo Illumina RNA-seq (this study and previously published in Maccaferri et al. 2019) plus long read ONT cDNA sequenced in this study and *T. aestivum* PacBio IsoSeq transcripts (HQ+LQ). Using the REAT transcriptome workflow (<https://github.com/EI-CoreBioinformatics/reat>), data were organised as two REAT transcriptome runs: 1) utilising short-read data newly generated in this study (90 samples representing 30 different tissue types across the whole plant growth cycle, Table S4), as well as 2) public *T. durum* short-read data (27 datasets from Maccaferri et al. 2019, [ID 473404 - BioProject - NCBI](https://www.ncbi.nlm.nih.gov/bioproject/PRJNA473404), Table S22). In addition, both runs also incorporated long read ONT cDNA sequencing data generated in this study on 36 sample combinations of the 174 RNA samples used in the Svevo Expression Atlas, Table S4b) and publicly available *T. aestivum* PacBio IsoSeq transcripts (Clavijo et al. 2017). ONT cDNA reads were oriented and trimmed with Pychopper v2.7.2 (https://github.com/epi2me-labs/pychopper).

HISAT2 v2.2.1 (Kim et al. 2019) was selected as the short-read aligner with IsoSeq transcripts aligned with minimap2 v 2.18-r1015 (Li, 2018), maximum intron length was set as 50,000 bp and minimum intron length to 20bp. IsoSeq alignments were required to meet 95% coverage and 90% identity. High-confidence splice junctions were identified by Portcullis v1.2.4 (Mapleson, et al. 2018). RNA-seq Illumina reads were assembled for each tissue with StringTie2 v2.1.5 (Kovaka et al. 2019), (Table S23) and Scallop v0.10.5 (Shao and Kingsford, 2017), (Table S24), while ONT cDNA reads were aligned with minimap2 and junctions filtered by 2passtools (Parker et al. 2021), the filtered set of high-quality junctions were provided to minimap2 in a second round of alignment. Aligned ONT reads were assembled with StringTie2, (Table S25). Gene models were derived from the RNA-seq assemblies and IsoSeq and ONT cDNA alignments with Mikado (https://github.com/EI-CoreBioinformatics/mikado, Venturini et al. 2018). For each of the two REAT transcriptome runs Mikado was run with all RNA-seq Scallop and StringTie2 assemblies, IsoSeq alignments and Nanopore assembled transcripts plus an additional run with only the long-read alignments and assemblies (Table S26).

*Cross-species protein alignment*

Protein sequences from 10 *Poaceae* species (Table S27) were aligned to the Svevo Rel.2.0 using the REAT Homology workflow (<https://github.com/EI-CoreBioinformatics/reat>) with options --annotation_filters aa_len --alignment_species Angiosp --filter_max_intron 20000 --filter_min_exon 10 --alignment_filters aa_len internal_stop intron_len exon_len splicing --alignment_min_coverage 90 --junction_f1_filter 40 --post_alignment_clip clip_term_intron-exon --term5i_len 5000 --term3i_len 5000 --term5c_len 36 --term3c_len 36. The REAT Homology workflow aligns proteins with spaln v2.4.7 (Gotoh et al, 2008) and filters and generates metrics to remove misaligned proteins. Simultaneously, the same protein set were also aligned using miniprot v0.3 (Li 2023) and similarly filtered as in the REAT homology workflow. The aligned proteins from both methods were clustered into loci and a consolidated set of gene models derived via Mikado (Table S28).

*Evidence guided gene prediction*

The evidence guided annotation of protein coding genes utilised the repeat annotation, RNA-seq mappings, transcript assemblies and alignment of protein sequences via the REAT prediction workflow. The pipeline has four main steps:

1. the REAT transcriptome and homology Mikado models are classified based on alignments to UniProt proteins to identify models with likely full-length CDS and which meet basic structural checks, i.e. having complete but not excessively long UTR’s and not exceeding a minimum CDS/cDNA ratio. A subset of gene models is then selected from the classified models and used to train the AUGUSTUS gene predictor (Stanke and Morgenstern 2005).
2. AUGUSTUS is run with extrinsic evidence generated in the REAT transcriptome and homology runs (repeats, protein alignments, RNA-seq alignments, splice junctions, classified Mikado models). Four evidence guided AUGUSTUS predictions are created using alternative bonus scores and priority levels for the various evidence types.
3. AUGUSTUS models, REAT transcriptome / homology models, protein and transcriptome alignments are provided to EVidenceModeler (EVM) (Haas et al. 2008) to generate consensus gene structures.
4. EVM models are processed through Mikado to add UTR features and splice variants.

Basic statistics of the alternative predictions are provided in Table S29.

*Projection of gene models from Triticum aestivum*

A reference set of hexaploid wheat gene models was derived from public gene sets (IWGSC and 10+ Wheat Genomes Project) projected onto the IWGSC RefSeq v2.1 assembly; a filtered and consolidated set of models was derived with Minos (<https://github.com/EI-CoreBioinformatics/minos>), with a primary model defined for each gene. Models were scored on a combination of intrinsic gene structure characteristics, evidence support (protein and transcriptome data) and consistency in gene structure across the input gene models. The Minos primary models were classified as full-length or partial based on alignment to a filtered Magnoliopsida Swiss-Prot and TrEMBL database. This assignment, together with criteria for gene structure characteristics and the original confidence classification, was used to classify models into 6 categories (Platinum, Gold, Silver, Bronze, Stone and Paper), with Platinum being the highest confidence category for models assessed as full-length, with an original confidence classification of "high", meeting structural checks for number of UTR and CDS/cDNA ratio and which were assessed as consistently annotated across the input gene sets. Reclassification resulted in 55319 Platinum, 24789 Gold, 11968 Silver, 61845 Bronze, 110518 Stone, and 115336 Paper genes. The four highest confidence categories Platinum, Gold, Silver and Bronze were projected onto the Svevo Rel.2.0 assembly with Liftoff-1.5.1 (Shumate and Salzberg 2021), only those models transferred fully with no loss of bases and identical exon/intron structure were retained (ei-liftover pipeline, https://github.com/lucventurini/ei-liftover).

Similarly, high confidence genes annotated in the hexaploid wheat *cv*. Chinese Spring IWGSC RefSeq v2.1 assembly were projected onto Svevo Rel.2.0 genome assembly with Liftoff, and only those models transferred fully with no loss of bases and identical exon/intron structure were retained. Among these, gene models with the attribute "manually curated" in the original IWGSC RefSeq v2.1 annotation were extracted as a set.

*lncRNA models*

Genes were annotated as lncRNA-related if all the corresponding transcripts passed a set of filters: to be defined as noncoding from both LGC and CPC2 tools (default parameters), a peptide length < 100 aa, an intronic size < 6000 nt and an annotation in RNAcentral <https://rnacentral.org/> as lncRNA or ncRNA or pre-miRNA or without annotation. Transcripts with an RNACentral annotations as rRNA, snoRNA, snRNA, tRNA, SRP-RNA were discarded (Wang et al. 2019; Kang et al. 2017).

*Gene model consolidation*

The final set of gene models was selected using Minos (<https://github.com/EI-CoreBioinformatics/minos>)**,** a pipeline that utilises metrics derived from proteins, transcripts, and expression data sets to create a filtered set of consolidated gene models. In this annotation, the following sets of gene predictions were filtered and consolidated into a single set of gene models using Minos:

- The gene models derived from the REAT transcriptome runs described earlier.
- The gene models derived from the REAT homology runs described earlier.
- The gene models derived from the REAT prediction run (AUGUSTUS and EVM-Mikado) described earlier.
- The gene models derived from projecting public and curated *T. aestivum* gene models of varying confidence levels onto the Svevo Rel.2.0 as described earlier.
- Iwgsc_refseqv2.1 models identified as "manually curated" projected onto the Svevo Rel.2.0 as described earlier.
- Manually annotated prolamin genes (coding and pseudogenes).
- lncRNA models.

Gene models were classified as biotypes protein_coding_gene, predicted_gene, transposable_element_gene, ncrna_gene, and pseudogene and assigned as high or low confidence based on the criteria below:

1. **High confidence protein_coding_gene:** Any protein coding gene where any of its associated gene models have a BUSCO v5.4.7 (Seppey et al. 2019) protein status of Complete/Duplicated OR have diamond (v0.9.36) coverage (average across query and target coverage) >= 90% against the listed protein datasets mentioned in the above paragraph “Projection of gene models from Triticum aestivum” or UniProt Magnoliopsida proteins. Or alternatively have average blastp coverage (across query and target coverage) ≥ 80% against the list protein datasets / UniProt Magnoliopsida AND have transcript alignment F1 score (average across nucleotide, exon and junction F1 scores based on RNA-seq transcript assemblies) ≥ 60%.
2. **Low confidence protein_coding_gene**: Any protein coding gene where all its associated transcript models do not meet the criteria to be considered as high confidence protein coding transcripts.
3. **High confidence transposable_element_gene**: Any protein coding gene where any of its associated gene models have coverage ≥ 40% against the combined interspersed repeats (see methods).
4. **Low confidence transposable_element_gene**: Any protein coding gene where all its associated transcript models do not meet the criteria to be considered as high confidence and assigned as a transposable_element_gene (see c).
5. **Low confidence predicted_gene**: Any protein coding gene where all the associated transcript models do not meet the criteria to be considered as high confidence protein coding transcripts. In addition, where any of the associated gene models have average blastp coverage (across query and target coverage) < 30% against the protein datasets mentioned AND having a protein-coding potential score < 0.25 calculated using CPC2 0.1 (Kong et al. 2007).
6. **Low ncrna gene:** lncRNA genes from the lncRNA analysis workflow.
7. **High confidence Pseudogenes**: From the manually curated prolamin genes.
8. **Discarded models**: Any models having no BUSCO protein hit and a protein alignment score (average across nucleotide, exon and junction F1 scores based on protein alignments) <0.2 and a transcript alignment F1 score (average across nucleotide, exon and junction F1 scores based on RNA-seq transcript assemblies) <0.2 and a diamond coverage (target coverage) <0.3 and Kallisto v0.44 (Bray et al. 2016) expression score <0.2 from across RNA-seq reads or having short CDS <30bp.

*Functional annotation*

The proteins were annotated using version 1.4.0 of the eifunannot pipeline (https://github.com/EI-CoreBioinformatics/eifunannot) in conjunction with AHRD v.3.3.3 (Hallab et al. 2014; <https://github.com/groupschoof/AHRD/blob/master/README.textile>). Sequences were used as queries in the BLASTN search against the reference proteins (Arabidopsis thaliana TAIR10, TAIR10_pep_20101214_updated.fasta.gz - https://www.araport.org) and the UniProt Viridiplantae sequences (data download 06-May-2023), both Swiss-Prot and TrEMBL datasets (The UniProt Consortium, 2014). Proteins were BLASTed (v2.6.0; blastp) with an e-value of 1e-5. We also provided InterProScan (v5.22.61; Jones et al. 2014) results to AHRD. We adapted the standard AHRD example configuration file path test/resources/ahrd_example_input_go_prediction.yml, distributed with the AHRD tool, changing the following apart from the location of input and output files:

- we included the GOA mapping from UniProt (ftp://ftp.ebi.ac.uk/pub/databases/GO/goa/UNIPROT/goa_uniprot_all.gaf.gz) as parameter 'gene_ontology_result',
- we also included the interpro database (ftp://ftp.ebi.ac.uk/pub/databases/interpro/61.0/interpro.xml.gz) and provided as parameter 'interpro_database',
- we changed the parameter 'prefer_reference_with_go_annos' to 'false'.

The following blast database specific weights were used: blast_dbs: i) swissprot: weight: 100, description_score_bit_score_weight: 0.2; ii) trembl: weight: 50; description_score_bit_score_weight: 0.4; iii) tair: weight: 50, description_score_bit_score_weight: 0.4

*Assessment of gene annotation*

Gene models were assessed via BUSCO v4.0.6 (Manni et al. 2021) with the poales_odb10 dataset and OMArk, v0.3.0 with clade Pooideae (Nevers et al. 2025). Gene models corresponding to BUSCO’s identified with a single A genome and single B genome copy were compared using multi_genome_compare (<https://github.com/EI-CoreBioinformatics/reat/tree/main/annotation/scripts/multi_genome_compare>); the average of identity + exon F1 and junction F1 (CDS exons only) was calculated for each AB gene pair.

In addition to the BUSCO analysis, the Svevo Rel 2.0 gene predictions were evaluated using 216 experimentally-determined genes (Maccaferri et al, 2019) and 6,241 Triticeae protein sequences of validated gene records in GrainGenes (<https://graingenes.org/cgi-bin/GG3/browse.cgi?class=gene>) kindly provided by Taner Sen. GMAP (version 2023-03-24; Wu and Watanabe, 2005) was used for cDNA mapping with the following parameters: --min-intronlength=20 --npaths=1 --ordered --min-identity=0.95, while miniprot (Li, 2023) was used for amino acid sequences requiring a fraction of positive amino acid matches > 0.95. Both sets of validated genes were mapped on both the Svevo Rel.2.0 and Rel1.0 genome assemblies, filtered for the best hit, and parsed for the match on the right homoeologous chromosome; the resulting gff3 files were intersected and compared with the respective reference annotations with BEDTools intersec (Quinlan and Hall et al. 2010) and gffcompare (<https://github.com/gpertea/gffcompare>, Pertea and Pertea, 2020) tools to estimate and compare the gene model accuracy. Gene structures for selected loci in Figure S7 were generated using the Gviz R-package (Hahne and Ivanek 2016).

*Transcription Factor Binding Sites (TFBS) and CpG-island prediction*

Regions of 2000 bps upstream the start codon of each predicted HC and LC gene and pseudogene were retrieved from the Svevo genome, based on the 5′ Untranslated Region (UTR) Svevo Rel.2.0 annotations. Overlapping regions were merged with BEDtools merge (<https://bedtools.readthedocs.io/en/latest/content/tools/merge.html>), and two sets of 15,541,069 and 18,367,054 regions were obtained for HC and LC genes and pseudogenes, respectively. The FIMO (Grant et al. 2011) tool from the MEME suite (Bailey et al. 2015) was used to scan these regions for the presence of motifs included in the Jaspar Core Non Redundant Plants Database (Castro-Mondragon et al. 2022), plus the motifs of the four canonical promoter elements: TATA-box (trained from unrelated plant promoter sequences), CCAAT-box, GC-box and Initiator, retrieved from the Expasy Promoter Elements Database (EPD: <https://epd.expasy.org/epd/promoter_elements/>) (Dreos et al. 2014). On the whole, Svevo 5’ UTRs were scanned with 660 motifs. FIMO results include absolute positions of motif matches on the Svevo genome, with associated score, p-value and q-value for each motif occurrence.

CpG-island prediction was carried out with CpGcluster (https://github.com/bioinfoUGR/cpgcluster) with the following parameters: d=50, P-value=1E-5.

**RNASeq analysis and generation of Durum wheat eFP Browser**

Raw Illumina reads of the 174 samples sequenced in this study (Table S4) were quality filtered and trimmed for adapter contaminants with Trimmomatic v0.39 (Bolger et al. 2014), then high quality reads were mapped to the Svevo Rel.2.0 genome and annotation (including both HC and LC protein-coding genes, transposable element genes, predicted genes, pseudogenes and ncRNA gene models) using STAR 2.7.10b (Dobin et al. 2013). Raw read counts and Transcripts Per Million (TPM) were calculated per gene using RSEM v1.3.3 (Li and Dewey, 2011). The structure of the datasets (i.e., sample dissimilarities between developmental stages, tissues, growth condition and inoculation together with replicate correlation) were revealed by count-based sample distance matrix and PCA plots using the R (https://www.r-project.org; v4.4.2) package DESeq2 (v1.40; Love et al. 2014). Finally, three TPM gene expression matrices, for i) plant development time-course dataset, ii) abiotic/nutritional stress response and iii) FHB infection profiling, were produced (Datasets 3-5).

Images depicting the approximate appearance (growth stage and plant organ) of the samples used for RNA extraction were drawn using Inkscape version 1.3.2. Images for the Durum Wheat eFP Browser were created using Adobe Illustrator (Adobe Inc.) and GIMP (The GIMP Development Team, 2019, available at: https://www.gimp.org). The parts of the images corresponding to the samples described in this paper were filled using unique colours using Adobe Photoshop (Adobe Inc.). Data as TPM values were databased on the BAR server located at the University of Toronto (Toufighi et al. 2005). XML configuration files for the three views presented in this paper (developmental map, abiotic stress, and biotic stress views) were generated using a custom tool. The Durum Wheat eFP Browser may be explored at <https://bar.utoronto.ca/~dev/efp_durum_wheat/cgi-bin/efpWeb.cgi>.

**Annotation of prolamin genes**

To identify and annotate glutenins (HMW-GS and LMW-GS), gliadins (α-, γ-, ω- and δ –types), and avenin-like proteins (ALP), representative sequences for each protein family were retrieved from previous studies (Kong et al. 2004; Gu et al. 2004; Chen et al. 2016; Huo et al. 2017; Huo et al. 2018a; Huo et al. 2018b; Huo et al. 2019; Zhang et al. 2018) and then used to perform BLASTN/BLASTP analysis against the Svevo Rel.2.0. All sequences identified were manually examined and classified according to their protein characteristics. The total of sequences identified includes both full-length open reading frame and sequences characterized by the presence of premature stop codons or frame shift mutations. Seven prolamin genes resulted in zero expression in RSEM expression matrix (Table S30) although one of them, HMW-B1 (TrturSVE1B02G00234400) is known to be expressed during seed development. Given the highly repetitive nature of the prolamin gene families, and the consequent challenges in accurately estimating their expression from RNA-seq data, transcript quantification analysis of prolamin genes was carried out using Salmon (Patro et al. 2017) in addition to RSEM. Salmon estimates transcript abundances using a lightweight mapping framework combined with a statistical model that probabilistically assigns reads to transcripts and corrects for sequence-specific and positional biases, providing robust TPM estimates even in the presence of highly similar transcripts. To ensure comparability with the RSEM analysis, Salmon quantification was performed using the same STAR-generated BAM files, allowing an independent evaluation of transcript abundance estimates. Data presented in Figure 2C are based on Salmon. Prolamin expression analysis performed with RSEM and Salmon are compared in Table S30. Considering the entire gene set, the correlation between RSEM and Salmon resulted to be very high (0.9) while at level of prolamin genes it was lower (0.5). The data confirmed the expression of six genes but not for ALP-A11 (TrturSVE7A02G01858940), which resulted zero in both analyses.

To compare the position of cysteine residues in B subgenome LMW-GS genes (excluding pseudogenes), the multiple alignment of amino acid sequences was conducted using Clustal omega (<https://www.ebi.ac.uk/jdispatcher/msa/clustalo>) with default parameters. To perform the comparison of chromosome sequences between chromosome 6A of this genome version and of the previous Svevo Rel.1.0, MUMmer4 v 3.23 (Marcais et al. 2018) was used with the default parameters. Heatmap was created starting from prolamin TPM expression values, log_2_ transformed and plotted in R using the Pheatmap package (https://cran.r-project.org/web/packages/pheatmap/index.html).

**Identification and annotation of Leucine Rich Repeat-Containing Receptors (LRR-CR)**

The analysis focused on the three gene families that encode receptors with an LRR domain and are key players in plant immunity (Han, 2019). The first includes LRR-Receptor Like Kinases (LRR-RLKs), which possess an extracellular LRR domain, a transmembrane domain, and an intracellular kinase domain. The second encompasses the LRR-Receptor Like Proteins (LRR-RLPs), which differ from RLKs by the absence of an intracellular kinase domain. The third one clusters the intracellular Nucleotide-binding Leucine-rich repeat Receptors (NLRs), characterized by a central NB-ARC (nucleotide-binding) domain and a C-terminal LRR domain. The N-terminal part of the protein is more variable and may contain either a TIR domain, an RPW8 domain or a DUF676 domain (Dievart et al. 2020).

Both full-length genes, which correspond to the expected intron-exon structures and are potentially functional, and gene copies containing nonsense mutations (i.e. internal stop codons, frameshifts, non-canonical GT-AG or GC-AG splicing sites and missing start or stop codons) were identified and annotated. For genes containing nonsense mutations (NsMCGs), all sequence fragments were recovered from the CDS of the original functional copy, i.e. before any nonsense mutations occurred. This ensures that the CDS of these NsMCGs can be translated into protein and compared with their full-length functional homologues. Annotation was performed using an iterative approach combining manual curation and the annotation transfer strategy described by Gottin et al. (2021). This approach uses a set of curated LRR-CR annotations to search the genome for regions with sequence similarity. Based on homology with the closest curated gene, the gene structure (intron/exon boundaries) of each identified region is derived.

The initial annotation was generated by transferring the curated Nipponbare LRR-CR annotation, as described by Gottin et al. (2021). Then, hundreds of the predicted Svevo LRR-CR annotations were manually curated based on the conservation of homology across monocotyledons and the available expression data for Svevo. A second annotation transfer was performed using the curated Nipponbare and the newly curated Svevo LRR-CR genes. This process was repeated three times. In total, 3,763 Svevo LRR-CRs were identified and annotated, with approximately one-third undergoing manual curation (Dataset 6, Tables S9 and S10). Dataset 6 is a .gff file of the 3,763 Svevo LRR-CRs.

LRRPROFLIER (Gottin et al. 2021) was used to identify, annotate and classify the LRR-CR protein sequences into gene families. The Svevo Rel.1.0 was scanned to identify LRR-CRs among both HC and LC predicted proteins. One protein per gene model was selected considering either the mRNA tagged as “representative=True”, if available, or the longest predicted protein. The resulting LC and HC predicted proteomes were then analysed using the LRRPROFLIER pipeline. LRRPROFLIER was also used to classify the LRR-CR predicted for Svevo Rel.2.0 (including those derived from the NsMCG gene models). In addition to characterising key domains of the three LRR-CR families studied (such as Kinase, NB-ARC, transmembrane, or Malectin), LRRPROFLIER also retrieved their positions and the total number of LRR motifs detected. The raw output of the two LRRPROFLIER runs, provided as a Dataset 7, was used for Table S11. For each analysed proteome (Svevo Rel.1.0 HC and LC predicted proteins, and Svevo Rel.2.0) the Dataset 7 contains three files: 1) LRR_classification_xx.csv which contains one line per gene with: gene ID / gene family (including F-box that we excluded from the analyses) / nb of LRR motif detected; 2) LRR_domains_filtered_xx.csv which contains one line per detected domain with 'gene ID / domain / first position in the predicted protein / last position in the predicted protein'; 3) LRR_structure_xx.html which contains a graphical representation of the detected domains for each gene.

**Physical map of the *Tg1-B* locus**

To provide evidence on the association between the 5 Mb inversion at the *Tg1-B* locus and the tough glume phenotype, the occurrence of the alternative orientation of the region in tetraploid germplasm panel having different domestication status and threshability efficiency was explored. The exact two inversion-breakpoints (distal at 39,735,777 bp on Zavitan WEWSeq v2.1 and 46,251,170 bp on Svevo Rel.2.0, proximal at 37,948,598 bp on Zavitan WEWSeq v2.1 and 50,790,070 bp on Svevo Rel.2.0) were identified to design markers to specifically detect the inversion (Figure 3A). The INV-M1 and INV-M2 three-primer assay were developed (for INV-M1 common primer: INV-M1-R1, 5’- CGTTTCGCTCTCCTTTCCAA-3’; specific for the Zavitan allele (600 bp): INV-M1-F1, 5’- CGTTTCGCTCTCCTTTCCAA-3’; specific for the Svevo allele (382 bp): INV-M1-F2, 5’- ATTAGTCATCGTCCGAGCGT-3’; for INV-M2: common primer: INV-M2-F1, 5’-GACAGCAGAGGAGGAGGAG-3’; specific for the Zavitan allele (369 bp): INV-M2-R1, 5’-TCCATTCCCCATTTCCACGA-3’; specific for the Svevo allele (228 bp): INV-M2-R2, 5’-GCCTGCCCCTCACTTACTTA-3’).

The marker INV-M2 was used to study the allelic diversity in 285 tetraploid wheat genotypes were selected from the Global Tetraploid Collection (Maccaferri et al. 2019) and from the Global Durum Panel (Mazzucotelli et al. 2020), resulting in the identification of 5 distinct haplotypes (H, Table S12). The M13-tailed primer method (Zhou et al. 2002) was utilized to label amplicons for visualizing PCR products, obtained by INV-M2, on a capillary DNA analyzer. The forward common primer was 5’-tailed with a 19-bp M13 sequence (5’-CACGACGTTGTAAAACGAC-3’) and was 5’-fluorescently tagged with 6-carboxyfluorescein (6-FAM). PCR was carried out in a 10 µL reaction volume containing 1 x PCR buffer, 1.5 mM MgCl2, 0.2 mM dNTPs, 0.1 µM of common forward primer (M13), 1 µM of each specific reverse primers and of 5’-fluorescently labelled M13 primer, 0.5 U Taq polymerase and 40 ng sample DNA. The thermal cycling conditions were as follows: initial denaturation at 94 °C for 3 min; 20 cycles of touch-down PCR comprising 94 °C for 45 s, 61/51°C for 45 s (- 0.5°C/s) and 72 °C for 60 s, followed by 23 cycles of 94 °C for 45 s, 51 °C for 45 s and 72 °C for 60 s, with a final extension at 72 °C for 10 min. The PCR fragments were separated using the Spectrum Compact Genetic Analyzer sequencer (Promega), with GeneScan ROX 1000 serving as the size standard. Visualization and sizing of the PCR fragments were conducted using GeneMarker® HID Software (Promega). In addition, the marker *Btr1-*A diagnostic for the Britte Rachis locus on chr. 3A (Zeng et al. 2020) was amplified with the following protocol. The PCR reactions contained 15 ng template DNA, 1.5 U of Taq polymerase (GoTaq DNA Polymerase, Promega), 1X Green GoTaq reaction buffer, 1.5 mM MgCl2, 400 mM dNTPs, and 500 nM of each primer in a total reaction volume of 10 µL with the following thermal profile: 3 min at 94 °C, followed by 38 cycles (30 sec 94 °C, 30 sec 62.5 °C, 20 sec 72 °C), and a final extension of 10 min at 72 °C. The PCR product was then digested with BstEII (Promega) following the manufacturer’s instructions. The *Brt1-B* marker was amplified with the following protocol. The PCR reactions contained 15 ng template DNA, 1.5 U of Taq polymerase (GoTaq DNA Polymerase, Promega), 1X Green GoTaq reaction buffer, 1.5 mM MgCl2, 400 mM dNTPs, and 500 nM of each primer in a total reaction volume of 10 µL with the following thermal profile: 5 min at 94°C, followed by 30 cycles (30 sec 94 °C, 30 sec 64 °C, 20 sec 72 °C), and a final extension of 10 min at 72 °C. The digested PCR and PCR products were then resolved on a 2% agarose gel.

***Tg1-B* locus haplotype characterization**

Haplotype diversity at the *Tg1-B* locus was characterised in a collection of 159 tetraploid wheat accessions (Lev-Mirom et al. 2026; Table S13) using whole-genome resequencing data (Dataset 8). A ~4.53 Mb chromosomal inversion on chr. 2B (BP1: 46,256,714 bp; BP2: 50,790,065 bp) was genotyped from whole-genome sequencing data (target ≥5× coverage; sequence data available under SRA project PRJEB61424; Table S13). Reads were mapped to the Svevo reference genome (Rel. 2.0) using bwa-mem2, and duplicate reads were removed with SAMtools markdup. Inversion genotyping was carried out by scanning alignments at each breakpoint for split reads whose supplementary alignment (SA tag) mapped to the opposite breakpoint (mapping quality ≥ 20; within ±1,500 bp), providing cross-validated evidence of the inversion junction. Accessions were classified as INV (ancestral orientation; ≥1 confirmed split read at both breakpoints), REF (Svevo-like derived orientation), or NO_CALL (local sequencing depth <3x). For a representative subset of accessions spanning the identified haplotypes, alignment files were additionally inspected in the Integrative Genomics Viewer (IGV; Thorvaldsdóttir et al. 2013) to confirm diagnostic signatures of the inversion, including split reads, discordant read pairs (anomalous insert size ~4.53 Mb; reversed R2R1 orientation), and coverage anomalies at the breakpoints. Inversion genotype calls were concordant with PCR marker results and with an independently derived haplotype table in 89.3% of sequenced samples (142/159); the 17 discordant accessions showed split-read evidence at only one breakpoint, consistent with low local coverage, and were therefore classified as INV.

SNP genotype data spanning chr. 2B: 50,790,476–50,792,842 bp of the Svevo Rel. 2.0 reference genome were extracted from a variant call file generated from the same resequencing dataset (Lev-Mirom et al. 2026, Dataset 8). A total of 24 biallelic SNPs were retained after quality filtering (Table S13). For each accession, genotypes were encoded as homozygous reference (REF), homozygous alternative (ALT), heterozygous (HET), or missing. Haplotypes were defined by the combination of alleles across all 24 SNP positions; only accessions with complete genotype data at all positions were used to define the canonical haplotype set. Accessions with missing data at one or more positions were assigned to a canonical haplotype if their observed alleles were fully compatible with exactly one complete haplotype; those compatible with multiple haplotypes were flagged as ambiguous, and those with no compatible haplotype were left unresolved. This procedure yielded 10 unique haplotypes from 126 fully typed accessions, numbered in descending order of frequency. Geographic origin data were incorporated from passport metadata accompanying the collection.

To construct the haplotype network, heterozygous and missing genotype calls were encoded as missing. Two accessions (WEW_PI_656869 and WEW_PI_656872) carrying divergent haplotype patterns inconsistent with *T. turgidum* subsp. *dicoccoides* were excluded, leaving 157 accessions. To distinguish haplotypes H2 and H3, which are identical at all 24 SNP positions but differ in inversion status, the chromosomal inversion genotype was appended as a 25^th^ binary character (Y = inversion present relative to Svevo; N = absent), as determined by split-read analysis described above. One sequence per haplotype was used as input, with the number of accessions per population group encoded in a PopART TRAITS block. A TCS statistical parsimony network was constructed in PopART v1.7 (Leigh and Bryant, 2015) using default parameters (connection limit 95%). Accessions were assigned to six population groups based on subspecies and geographic origin: durum wheat landraces (DWL), Ethiopian durum landraces (DWL_ETH), domesticated emmer (DEW), Ethiopian emmer (DEW_ETH), wild emmer from the Southern Levant, Israel, Jordan, Lebanon, Syria, and Palestine, (WEW_SL), and wild emmer from the Northern Levant, Iraq, Iran, and Turkey, (WEW_NL). The network was exported as SVG and redrawn for publication with node sizes scaled to the square root of haplotype frequency.**Projection of the durum QTLome onto the Svevo Rel.2.0**

All Quantitative Trait Loci (QTL) that had been mapped as of 31^st^ December 2023 for major trait categories in *T. turgidum* biparental populations by linkage mapping or by genome-wide association studies (GWAS) in tetraploid wheat collections, were considered for projection onto the Svevo Rel.2.0 assembly.

These trait categories include phenology, grain yield and its components, disease resistance, abiotic stress tolerance, grain quality and root architecture. For each QTL, a confidence interval (CI) was defined, based either on Darvasi and Soller (1997) in linkage mapping populations, or on the rate of LD decay in germplasm collections for GWAS. Where QTLs for different years/locations mapped in the same regions in the same study and for the same trait, only the most consistent QTL was retained for projection. Then, before anchoring to the genome assembly, each QTL/CI was redefined using either the tetraploid wheat consensus map (Maccaferri et al. 2015) or the DArT v.4 wheat consensus map (Sansaloni et al. 2020), to select genetic markers that were located as close as possible to the extremes of the confidence interval, taking advantage of the high density of markers. The consensus map was chosen for each target QTL according to the genotyping technology used for mapping: SSR, DArT, SNP (Illumina iSelect wheat 90K array), or DArT-Seq. The relative positions of QTL flanking markers shared between the linkage map and the selected consensus map were then used to calculate the ratio of genetic distances between the original map and the consensus map. Based on this ratio, the original CI was recalculated onto the consensus map and the nearest flanking markers were identified. Their physical location on the Svevo Rel.2.0 genome assembly defined the most likely anchoring position of the QTL on the durum wheat reference genome.

The position of the QTL peak was mapped onto the consensus map as the midpoint of the CI and was then redefined on the genome assembly in a similar way to the confidence extremes. QTLs identified through association mapping were managed in a similar way, with the only difference being that the CIs were defined directly on the consensus map based on the genetic position of the QTL peak marker ± the mean genetic distance of LD decay. If this procedure was not feasible, e.g. due to the unavailability of markers shared between the original and consensus maps or inconsistent genetic/physical positions, QTL were directly anchored on the Svevo Rel.2.0 genome by locating the physical position of the QTL peak marker. In these cases, the confidence interval was set according to LD calculated for germplasm collections of a similar composition: 1 cM = 5 Mb for wild collections, 2/3 cM = 9 Mb for durum landrace collections, and 5–6 cM = 12 Mb for durum variety and breeding line collections. The overall list of tetraploid QTLs anchored on Svevo Rel.2.0 constitutes the durum QTLome (Table S14).

The durum QTLome was inspected for a few specific traits, such as Thousand Kernel Weight (TKW), and for trait macro-categories, such as root architecture and function, drought tolerance and resistance to diseases and pests, to identify clusters of QTLs. These clusters are known as QTL hotspots and are defined as genomic regions where at least three QTLs, identified in different germplasm backgrounds for the same trait/category, overlap their corresponding confidence intervals and have peak markers at a close distance. The minimum overlapping region was defined for each QTL hotspot based on the positions of the most internal flanking markers. Candidate genes were sought within QTL hotspots with a minimum region of less than 10 Mb. Hotspot regions including QTLs for more than one disease or pest were inspected for genes annotated as disease resistance related on Svevo Rel.2.0 and the sequences of common wheat resistance genes retrieved from WheatOmics website ([wheatomics.sdau.edu.cn](http://wheatomics.sdau.edu.cn/)) were used in BLAST analysis against Svevo Rel. 2.0 genome to verify their co-location with the hotspot regions identified in this work, even if locating just outer than the minimum overlapping interval. For the other target traits, to enhance the informativeness of gene annotation, the classification of *Oryza sativa* genes for trait ontology available from the Rice Annotation Project ([RAP-DB | HOME](https://rapdb.dna.affrc.go.jp/index.html)) was leveraged to inform the annotation of homologous durum wheat gene. All rice genes associated with the trait ontologies targeted by the QTLome inspection were downloaded and used in BLAST analysis against Svevo Rel. 2.0 to identify the corresponding homologues. For the durum wheat candidate genes of QTL hotspots, the expression profile in different plant organs or in response to Fusarium or drought stress was inspected through the dedicated eFP browser ([Durum Wheat eFP Browser](https://bar.utoronto.ca/~dev/efp_durum_wheat/cgi-bin/efpWeb.cgi)). The relative expression in response to biotic/abiotic stress was considered (with a 2- fold threshold), while the absolute expression level was verified to assess gene expression in specific tissues or developmental stages.

**Exome sequencing analysis, SNP calling and haplotype-based ancestry inference**

The exome capture sequencing dataset (see Genetic materials) composed of 287 tetraploid wheat accessions (Dia Sow et al. 2025) was re-mapped on the Svevo Rel.2.0 genome assembly using bwa-mem (Li 2013) with default settings. Resulting BAM files were then sorted and filtered for uniquely mapping reads using SAMtools 1.21 (Danecek et al. 2021), and duplicate reads were removed using Picard MarkDuplicates (<https://broadinstitute.github.io/picard>). Variant calling was performed with GATK4 (Genomic Analysis toolkit), following the best practice workflow (Poplin et al. 2017): HaplotypeCaller and GenotypeCaller tools were used for variant calling and joining, respectively. Low quality calls ('QD < 2.0 || FS > 60.0 || MQ < 40.00 || SOR > 3.0'; QD = Quality by depth; FS = Fisher strand; MQ = RMS Mapping Quality; SOR = Strand Odds Ratio) were filtered out, while only high confidence SNPs (GQ - Genotyping Quality > 10 and DP - Depth > 5 in at least the 40% of the samples) were retained. Filters were applied using BCFtools (Danecek et al. 2021) and VCFtools (Danecek et al. 2011). After marking the heterozygous calls (0/1) as missing data, mean depth statistics and the percentage of missing data were calculated for each individual accession using VCFtools. Accessions with a low mean depth level (DP < 4) or a high percentage of missing data (more than 30%) were excluded from the dataset, resulting in 260 retained accessions. Only sites with minor allele count > 4 were kept, resulting in 778,448 SNPs in the final vcf file. Missing genotype calls were imputed with Beagle5.3 (Browning et al 2021) with the following settings: window=10 overlap=2 iterations=100 impute=false, and ne=30000.

Population structure was analysed at different values of sub-population runs ranging from K = 2 to K = 20 with 100 test sets for cross-validation using fastStructure (Raj et al. 2014). At optimal K-value of 16, we observed two specific subclusters for wild emmer wheat (T. turgidum ssp. dicoccoides) corresponding to Southern Levant Fertile Crescent and Turkey populations (Q01_WEW_ISR/SL and Q02_WEW_TRK, both composed of 21 accessions) and one group of GGAA-type tetraploid wheat (Q00_AAGG, including twelve *T. araraticum* and *T. timopheevii* accessions). These three ancestor subpopulations were also supported by Maximum-Likelihood (ML) tree inferred with iqtree (Minh et al. 2020).

The filtered vcf file including the 680,638 polymorphic SNPs from the 54 ancestor accessions (Dataset 9, Table S18) was then converted into the “geno” matrix format using the LEA R package (v3.10.2, Gain and Francois 2021), then parsed in non‐overlapping windows of 100 consecutive SNPs across the 54 wild‐relative accessions. Within each window, we computed a pairwise distance matrix reflecting the percentage identity of variant calls between every accession pair. To interrogate local ancestry, we applied k- means clustering (scikitlearn v1.2.2, Pedregosa et al. 2011) across k = 3–20, optimizing cluster compactness via inertia. For each window, the cluster harbouring the accession with the minimal summed SNP score (i.e., the greatest similarity to the durum reference Svevo) was designated as the “Svevo‐proximal” group. We then quantified the proportional membership of this cluster—painting each 100SNP window by the percentage of accessions assigned to it—and overlaid both the raw count of cluster members and the minimal summed SNP score, thereby providing a high‐resolution landscape of putative introgression tracts.

**Gene mapping and progenitor attribution**

A curated set of (n=396) cloned developmental and stress‐response genes from hexaploid wheat (*T. aestivum*) was functionally annotated and aligned to Svevo Rel.2.0 using Minimap2 (spliceaware, 1based PAF output, Li 2018). Only high‐confidence alignments (MAPQ > 30) were retained to disambiguate paralogous mappings. These alignments were intersected with Svevo gene models via BEDTools (Quinlan and Hall et al. 2010) to define orthologous gene coordinates. Each gene interval was stratified into distal, pericentromeric, or centromeric compartments based on recombination‐rate gradients. To infer progenitor lineage, we computed sequence‐identity and coverage distributions between Svevo intervals and wild emmer wheat panels from the Israel/Southern Levant (WEWISR/SL, “Q01”), Turkey (“Q02”), and *T. timopheevii* (“AAGG”, “Q00”), then applied empirical percentage thresholds (75^th^, 95^th^, 99^th^) to assign each gene segment to its most likely wild ancestor. This integrative framework yields a fine‐scale portrait of functional gene ancestries within the tetraploid wheat genome.

**Results**

**Genetic analysis of *Tg1-B* locus**

Besides the panel of 159 accessions described in the main text, we preliminarily explored the occurrence of the ~5 Mb inversion in a larger tetraploid germplasm panel having varying degree of threshability based on their domestication level. The panel was comprised of 185 modern durum or durum landraces and 30 primitive tetraploid wheat accessions, all having free-threshing spikes, as well as 45 domesticated (ssp. *dicoccum*) and 25 wild emmer (ssp. *dicoccoides*) genotypes (Table S12a). A marker tagging the inversion (INV-M2, Figure 3A) was verified by amplicon sequencing and employed to assess the germplasm a panel. INV-M2 amplified a 369 bp fragment in Zavitan (haplotype H2) and a 228 bp fragment in *cv.* Svevo (H4). Notably, the long and short amplicons were present in the tetraploid germplasm collection with a range of frequencies, with additional variations within these two major groups which were classified as insertions/deletions (Figure S16). Amplicon sequences of haplotypes were aligned against Svevo Rel.2.0 and Zavitan WEWSeq v2 genomes to verify their association with the 5 Mb inversion, in addition to confirming the target locus on chr. 2B (Figure S15). We identified five distinct haplotypes (Table S12b). Haplotypes H1, H2 and H3 were exclusive of hulled accessions and differ by a 6 bp deletion in H2, and an 11 bp insertion in H3. Notably, H5 was found only in *T. turgidum* ssp. including *durum* and other primitive subspecies, while H4, characterized by a 33 bp insertion compared to H5, mainly occurred in free-threshing genotypes (96%, with the exception of five wild and four domesticated emmer, with one wild emmer accession having the domesticated *Btr1* allele and thus likely misclassified). Sequences from haplotypes retrieved in free threshing genotypes (H4, H5) aligned at a unique site on Svevo Rel.2.0, while were bipartite at two sites 6 Mb apart by BLAST analysis against Zavitan WEWSeq v2.1, thus demonstrating their association with the ~5 Mb inversion. An opposite result was observed for amplicon sequences of haplotypes retrieved in hulled genotypes (H1, H2, H3). These findings confirm that the ~5 Mb inversion on chr. 2B is strongly associated with the free-threshing phenotype and suggest that it played a key role in the transition from hulled to free-threshing wheat during domestication.

**The durum wheat QTLome**

To define the durum QTLome, all QTLs that had been mapped using either linkage mapping or genome-wide association studies (GWAS) were gathered as of 31 December 2023. QTL confidence intervals were calculated based on the same rules applied to all loci. The genetic positions of QTL flanking and peak markers were then converted into physical genomic coordinates by using BLAST analysis of the corresponding marker sequences to Svevo Rel.2.0, either via an intermediate projection step onto available consensus maps to increase mapping resolution or directly onto the reference genome. Clusters of QTLs, known as QTL hotspots, were identified when at least three QTLs, which had been detected in different germplasm backgrounds for the same trait/category, overlapped in their respective confidence intervals and had peak markers in close proximity.

The 6,621 QTLs were mapped onto the Svevo Rel.2.0 assembly. They were grouped by macro-trait category as follows (Table S14):

- 680 QTLs for phenology;
- 1,692 QTLs for grain yield-related traits (e.g. number of fertile tillers, grain number per spike and spikelets, grain weight and yield)
- 215 QTLs for biomass-related traits;
- 544 QTLs for traits describing the morphology of epigeal plant organs (e.g. morphological traits of spikes, stems and leaves, including 313 QTL for plant height);
- 618 QTLs associated with root architecture and function;
- 1,046 QTLs associated with resistance to wheat pathogens (including all rusts, Zymo*septoria tritici*, *Parastagonospora nodorum* blotch and *Fusarium*-related diseases, e.g. FHB and crown rot, tan spot, wheat blast and powdery mildew, as well as minor diseases such as loose smut and ergot, and pests such as Hessian fly and stem sawfly).
- 802 QTLs associated with abiotic stress resilience, mainly in relation to physiological traits of stress tolerance or stress indices calculated based on a range of plant performance traits;
- 890 QTLs associated with grain/flour traits related to technological and nutritional quality;
- 134 QTLs associated with other minor traits such as domestication and selection, and germination-related traits.

To prove how the durum QTLome can allow for the definition and refinement of genomic regions of relevant agronomic traits at higher resolution, QTL hotspot regions were defined for TKW and a few traits macro-categories (root architecture and function, drought tolerance, disease, and pest resistance) and then inspected for candidate genes, supported by functional evidence of homologous genes in related crop species (bread wheat and rice), or solely based on evidence of gene expression and gene annotation in durum wheat (Table S15 and S16).

**Thousand Kernel Weight (TKW):** a candidate gene was pointed out for 3 hotspots, besides the two described in the main text. The TKW_1 on chr. 1A (9.3 Mb with 97 HC genes) encompassed the gene *TrturSVE1A02G00098520* which is the homologue of *OsSec18* that regulates vacuolar morphology in rice endosperm cells and whose overexpression was found associated to lower grain weight (Sun et al. 2015). Notably, *TrturSVE1A02G00098520* exhibited high expression levels during the seed development phases at 3 DPA and 5 DPA, but also in inflorescence organs, and a noticeable expression in the flag leaf and peduncle tissues. For the TKW_21 on chr. 6A (5.7 Mb and 54 HC genes), both functional and gene expression evidence support the candidate gene *TrturSVE6A02G01661230*. Indeed, its expression increased during pistil and seed development reaching a maximum at 3 DPA, but also in flag leaves following senescence progresses with a maximum at 30 DPA, besides being highly expressed in other plant organs. It encodes for a glutamine synthetase-like protein, and notably it is homologue to the gene *TaGS1c* which contributes to remobilization of vegetative nitrogen to developing grain in bread wheat (Tian et al. 2015). A role in protein content was suggested also for the candidate gene of the hotspot TKW_13, the gene *TrturSVE4B02G01133480,* which has homology relationship with the rice gene *OsTudor-SN*. This gene encodes for a RNA-Binding Protein, a multifunctional protein required for storage protein expression, seed development and protein body formation during endosperm development (Chou et al. 2019).

To discover other candidates, HC genes at the smallest TKW hotspots were also evaluated for the gene expression profile combined with the gene annotation (Table S16). Besides the example reported in the main text, in TKW_15 on chr. 4B, two genes (*TrturSVE4B02G01216530* and *TrturSVE4B02G01216700*) out of the 14 HC identified were expressed in early seed development stages and encode a phosphoserine aminotransferase and a DEAD-box ATP-dependent RNA helicase, respectively, while *TrturSVE4B02G01216710*, encoding a photosynthetic NDH subunit of subcomplex B2, was found highly expressed in flag leaf and peduncle. Two small hotspots, TKW_4 and TKW_26, where 4 and 9 HC genes were detected respectively, no genes with an informative expression pattern were identified.

**Drought resilience**: QTLs for traits associated to leaf water content and leaf transpiration were considered, including Water Use Efficiency (WUE), Osmotic Adjustment and Osmotic Potential (OA and OP), Canopy Temperature Depression (CTD), Carbon Isotope Discrimination (CID), Stomatal Conductance (StC), Relative Water Content (RWC), and Transpiration Rate (TrR), for a total of 137 QTLs. Among them, 22 QTLs were clustered in seven hotspot regions, which span from 2.1 to 9.7 Mb. We were able to hypothesize candidate genes for five hotspots based on phenotypic evidence related to drought resilience in rice or wheat. They were mostly proteins with a role in signal transduction or transcriptional regulation as likely for annotations like “C2 domain containing protein” or “Homeobox domain-containing protein” found in the Drought_4, 6 and 7 hotspots, or with a protective role, as suggested by “Late embryogenesis abundant protein” annotation and the Δ1-pyrroline-5-carboxylate dehydrogenase (P5CDH) for proline catabolism, both falling within the Drought_2 hot spot. A significant and fast drought responsiveness was observed only for the expression level of the WRKY transcription factor *TrturSVE1A02G00102630* in the hotspot Drought_2. The candidates *TrturSVE5A02G01391030* and *TrturSVE2A02G00436070* of the hotspot Drought_6 and 4, respectively and 2.1 and 7 Mb wide on chr. 5A and 2A, are durum wheat gene homologues of the rice homeodomain transcription factor *OsHB22* and of the protein transporter *OsFTIP6.* Notably, OsFTIP6 mediates the nucleocytoplasmic shuttling of OsHB22 in rice and both belong to a regulatory protein module of the drought response (Yang et al. 2022).

**Root morphology and function:** some correspondences with a gene known for its role in root morphology/development were found for 22 hotspots.

Candidate genes for root traits were searched by analysing gene expression profiles of HC genes in small QTL hotspot regions encompassing a few annotated genes as well as . Alternatively, based on homology with genes characterised in model cereal species as rice . An example of a candidate gene translated from rice is that of the hot spot region Root_9, which groups seven QTLs from three studies (Maccaferri et al. 2016; Rosello et al. 2019; Mehrabi et al. 2020) in 7.1 Mb on the short arm of chr. 2B (8.1-15.2 Mb). Within the confidence interval of this region, the high confidence gene *TrturSVE2B02G00449510* has been identified (13,127,609-13,131,745 bp), which corresponds to the rice gene *BR6ox_2/OsCYP85A1/OsDWARF*, coding for a brassinosteroid‐6‐oxidase 2 involved in the biosynthesis of brassinosteroids, hormones with a role in regulating cell wall composition in different organs (Percio et al. 2025). A role for *BR6ox_2* gene in root development in drought conditions has been demonstrated for spinach (Duan et al. 2017). Interestingly, the gene *TrturSVE2B02G00449510* is strongly overexpressed in roots following drought stress (4 hours) in durum wheat seedlings (Table S16).

**Disease and pest resistance**: The forty-six hotspot regions identified for diseases and pests are spread on different chromosomes, except 3A and 4B, and the chromosome with the highest number of hotspot regions was 6A with seven regions, followed by 4A with six regions respectively, and 2B, 5B, 7A and 7B with five hotspot regions. Most of the hotspots included QTLs for resistance to more than one rust species, for example those located on chr. 7B including QTLs for leaf, stem and stripe rust resistance, and all regions included at least one QTL for rust resistance, except three regions on chr. 1A (FHB-TS), 5B (TS-SNB) and 6B (PM-CR). Some of the QTL included in the hotspot regions were found in the same conditions/treatments/plant stages for all the diseases involved, for example the hotspot Disease_4 or 9, involving QTL identified under artificial inoculum for specific races, at seedling stages and in controlled conditions. Nevertheless, most cases were located in regions where QTL found in different conditions and plant stages are located, such as hotspot Disease_37 or 46 that included QTL identified for diseases evaluated in open field and in greenhouse, at adult plant stage and seedling, respectively, and finally under natural and artificial infection. Moreover, in all cases QTL contributing to each region were identified in different genetic backgrounds, including landraces, modern cultivars and breeding lines.

The number of candidate HC genes related to diseases identified within the hotspots (disease resistance proteins, kinases, wall-receptor proteins, sugar transporters, pathogenesis-related proteins, callose synthases) ranged from one in the hotspot regions n. 3, 9, 13 and 38, to thirty in Disease_5 and 40. Interestingly, the hotspot Disease_38 involving QTLs for powdery mildew and crown rot resistance and spanning 2.4 Mb, was characterized by a unique durum wheat gene with a function related to disease resistance (*TrturSVE6B02G01828340*) and did not include any NsMCG. *TrturSVE6B02G01828340* is annotated as disease resistance protein Pik-2-like, which belongs to a class of genes able to interact with a pathogen avirulence (*Avr*) gene, resulting in the so-called race-specific resistance.

Moreover, hotspot disease regions were inspected for the presence of durum wheat genes with significant sequence similarity to known bread wheat resistance genes retrieved from the WheatOmics website. For instance, the putative homologous of *Lr10* (Feuillet et al. 2003), already characterised for allelic diversity in tetraploid wheat (Loutre et al. 2009) was mapped close to the hotspot Disease_1 on chr. 1A, although no QTL for leaf rust resistance were included in that region. Analogously, the homologous gene of *Sr27*, first identified in a wheat line carrying an introgression of the 3R chromosome from rye, and effective against Ug99 (Upadhyaya et al. 2021), was found in the Disease_37 where many QTLs effective against different races of *Puccinia graminis* f. sp. *Tritici,* out of which TTKSK, were mapped. Interestingly, genes encoding Mediator complex subunit 15 KIX domain-containing proteins were annotated on chr. 7A and 7B (*TrturSVE7A02G02005600* and *TrturSVE7B02G02161520*) close or within two hotspots (Disease_42 and 46, respectively) and showed a high level of similarity to the bread wheat genes *SuSr-D1-7A* and *SuSr-D1-7B-1*. Notably, the locus *SuSr-D1* on chromosome 7D*,* encoding the Med15b.D subunit of the conserved Mediator complex, was found associated to suppression of stem rust resistance (Hiebert et al. 2020). Indeed, in the Disease_46 region on 7B QTLs for yellow and brown rust resistance only were found. Finally, two durum wheat genes (*TrturSVE7B02G02156770* and *TrturSVE5A02G01386300*) involved in powdery mildew resistance were found to be very similar to the bread wheat genes *Pm5e* on chr. 7B and *TaMLO-A1* on chr. 5A. Notably, the known durum wheat gene *Sr13* (Zhang et al. 2017) is very close to the hotspot Disease_34, while *Lr14* (Kolodziej et al. 2021), present in both common and durum wheat but absent in Svevo Rel.1.0, was genetically anchored on Svevo Rel.2.0 under the hotspot Disease_46.

Considering the seven hotspots, including at least two known QTLs for FHB resistance, the inspection for genes expressed in response to Fusarium infection gave positive results in all cases (Table S16). Disease_14 contains the gene *TrturSVE4A02G00978380* that was 94-fold more expressed upon infection in the rachis after 4 days (Figure S17), while in Disease_21 two genes, out of the three annotated as disease-related genes, showed 215-fold and 58-fold upregulation in response to Fusarium (*TrturSVE5A02G01361870* and *TrturSVE5A02G01361880*, respectively). Other examples were four genes in the hotspot Disease_1 (*TrturSVE1A02G00001740*, *TrturSVE1A02G00001930*, *TrturSVE1A02G00002020*, and *TrturSVE1A02G00003110*), among the ten annotated as disease-related, which were overexpressed in the rachis at 4 days after inoculum. Other two genes were found over-expressed in spikelet and rachis (*TrturSVE2A02G00296000* and *TrturSVE2A02G00296110*, respectively) under the hotspot Disease_6, whereas under the hotspot Disease_7 only one out of the annotated genes (*TrturSVE2A02G00440490*) was overexpressed in the spikelet both after 2 and 4 days after the experiment.

**Inference of durum genome ancestry back to wild emmer wheat**

The ancestry landscape of the *T. durum* cv. Svevo genome, resolved through 8,372 haplotype windows (100 -SNP intervals), revealed pronounced spatial and progenitor-specific biases. The Svevo genome exhibited pronounced ancestry stratification (Table S18), with Wild Emmer Wheat from Israel/South Levant (WEW-ISR/SL) contributing 41.96% (2,089.42 Mb) and 42.95% (2,198.96 Mb) of genome content to the A and B subgenome respectively, at 75% conservation threshold, dwarfing contributions from WEW-Turkey (34.01% to each A and B subgenome; 1693.53 Mb and 1497.77 Mb, respectively) and *T. timopheevii* (AAGG; 0.34%, 17.43 Mb). Conservation diminished at higher thresholds (95%/99%), retaining a total 68.02% (3,191.3 Mb) of WEW-ISR/SL ancestry versus 36.17% (1,821.51 Mb) for WEW-Turkey, while *T. timopheevii* contributions remained negligible (<0.24%). Genomic architecture further shaped these patterns: gene-dense distal regions retained 18.46% WEW-ISR/SL ancestry (vs. 12.47% WEW-Turkey), whereas pericentromeric and centromeric regions conserved larger haplotype blocks (29.34–37.1% for WEW-ISR/SL; 19.73–34.82% for WEW-Turkey), reflecting recombination-driven selection.

Distal regions consistently show higher WEW-Turkey membership, suggesting that recombination‐rich chromosomal arms were preferentially “captured” from the Turkish wild pool, perhaps for adaptive alleles that conferred stress tolerance or yield components. Pericentromeric regions often lean toward WEW-ISR/SL, hinting that reduced recombination there has preserved large ancestral haploblocks—potentially sheltering clusters of interacting genes (e.g. vernalization and flowering‐time regulators) that breeders unknowingly maintained. The heterogeneous distribution of signals along each chromosome argues against a simple single‐origin model: Svevo’s genome is a dynamic patchwork forged by both Levantine and Anatolian wild‐emmer contributions.

Notably, critical domestication genes were traced to WEW-ISR/SL at stringent thresholds (Table S20). *Pinb-A1* (grain texture) and *Rht-1* (plant height), retained exclusively at 99%, localized to distal regions enriched for ancestral diversity. Similarly, *Vrn-A1* (flowering time) and *Q* (spike architecture) persisted in WEW-ISR/SL-derived blocks, underscoring their roles in adaptive selection. In contrast, phenology-associated *FT* alleles on chromosome 3A originating from WEW-Turkey, were conserved only at the 75%, indicative of weaker selection. This asymmetry extended to functional categories: 39.9% of developmental genes (e.g., *AP1* on chr. 7A) and 34.8% of stress-response genes (*Fhb1* on chr. 3B) traced to WEW-ISR/SL at 95%, while WEW-Turkey contributions were restricted to lower thresholds, aligning with its diminished genomic footprint.

**Supporting figures**

**Figure S1 Hi-C contact map comparison between Svevo Rel2.0 and Rel1.0 chromosome assemblies.** The heatmaps represent the pairwise intra-chromosomal (**A**) and inter-chromosomal (**B**) contact frequencies of the Hi-C reads mapped to the Svevo Rel.2.0 and Rel.1.0 assemblies. The intensity of the colour corresponds to the interaction frequency.


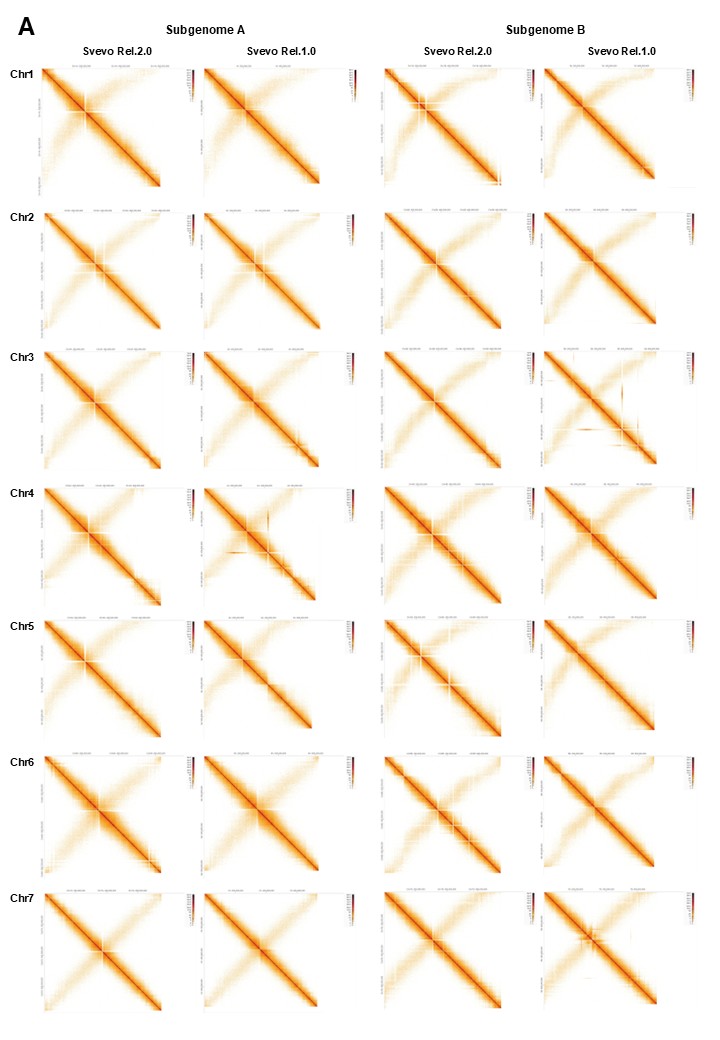


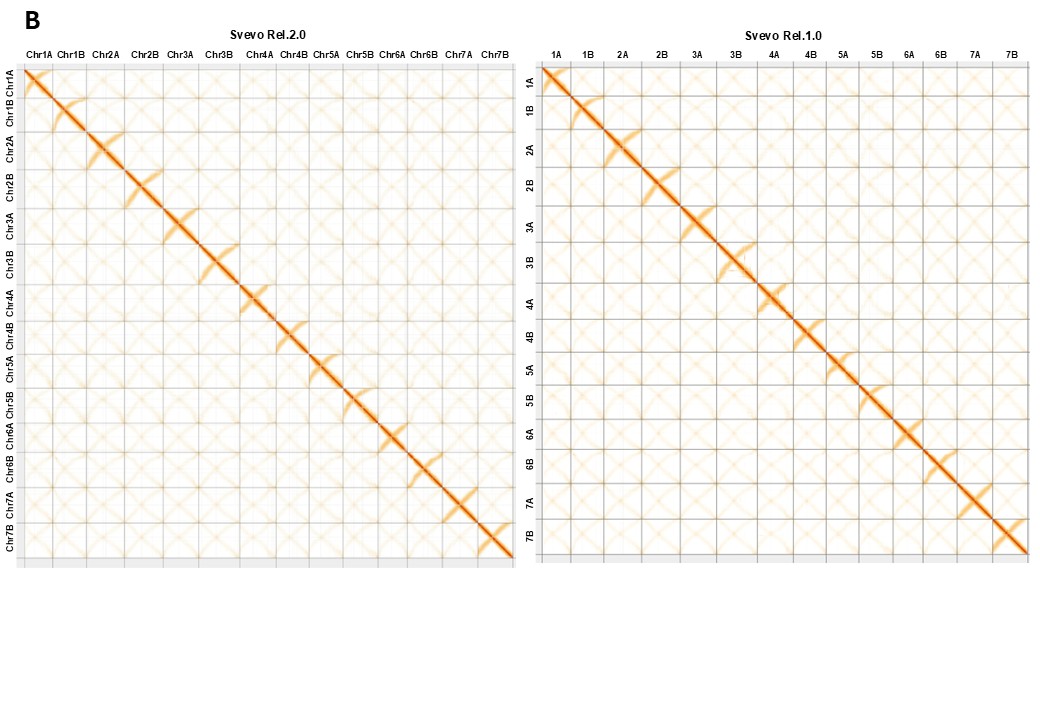


**Figure S2 Prediction of the centromeric domain in chr. 5B from Hi-C data.**

The genomic interval corresponding to the putative centromeric domain was identified as a low-interacting region at the intersection of the diagonal and the antidiagonal in the Hi-C contact probability heat map. The diagonal-antidiagonal cross pattern, visible in the inset showing the whole 5B chromosome, reflects Rabl organization of Svevo chromosomes.


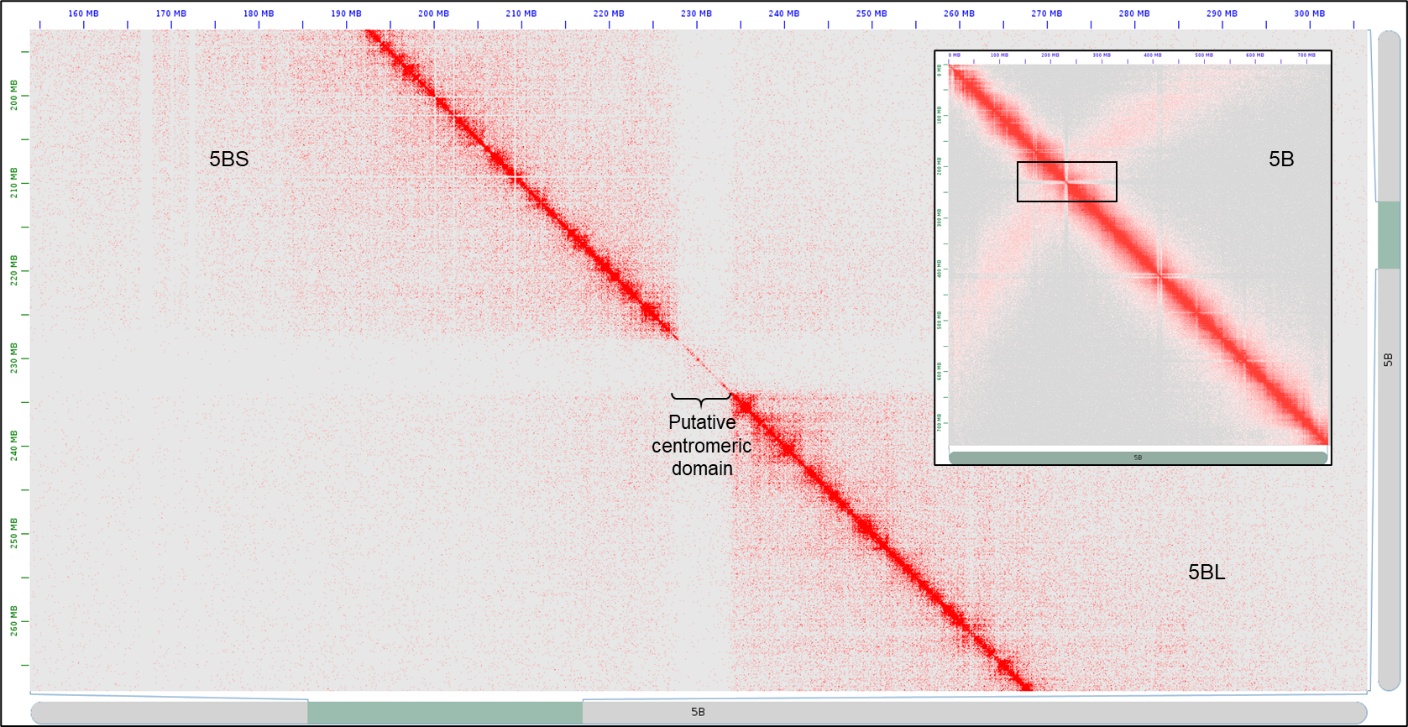


**Figure S3** **Major rearrangements detected between reference durum, wild emmer and bread wheat genome assemblies.** The following assemblies were compared: Svevo Rel.2.0 and Svevo Rel.1.0 (Maccaferri et al. 2019), or Zavitan WEWSeq v2.1 (Zhu et al. 2019), or Chinese Spring IWGSC RefSeq v2.1 (Zhu et al. 2021). The plots identify sequence collinearity across the diagonal with blue dots, and inversions (+/- alignment orientations) between the two genomes as red dots. Duplicated genome segments are indicated by red or blue dots positioned away from the diagonal.


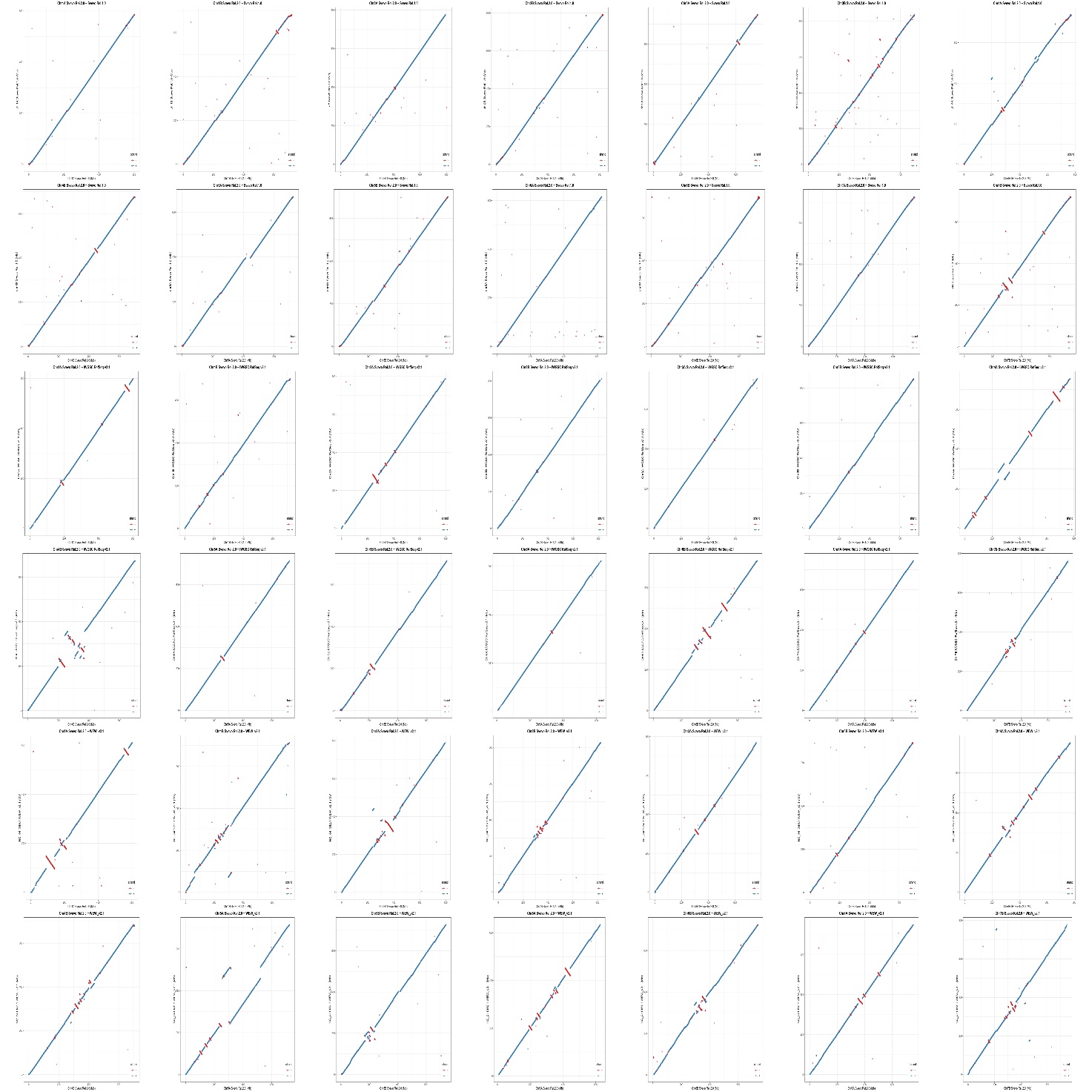


**Figure S4 Sub-genome distribution of the main TE classes in Svevo Rel2.0 durum wheat genome wheat.** Table and plot provide a summary of the relative TE distribution between the A and B sub-genomes of Svevo durum wheat. TE acronyms are as follows: RLC (T*y1/copia*  LTR–RT), RLG ( *Ty3/gypsy* LTR–RT), RLX (unclassified LTR-RT), RIX (LINE non-LTR RT), RSX (SINE non-LTR RT), RXX (unclassified RT), DTX (unclassified DNA transposon), DTA (*hAT* DNA transposon), DTC (*CACTA* DNA transposon), DTM (*Mutator* DNA transposon), DTT (*Tc1–Mariner* DNA transposon), DXX (MITE DNA transposon), DTH (PIF– Harbinger DNA transposon).

**
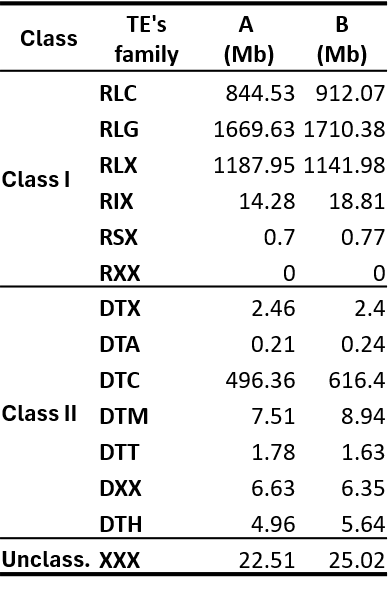
**
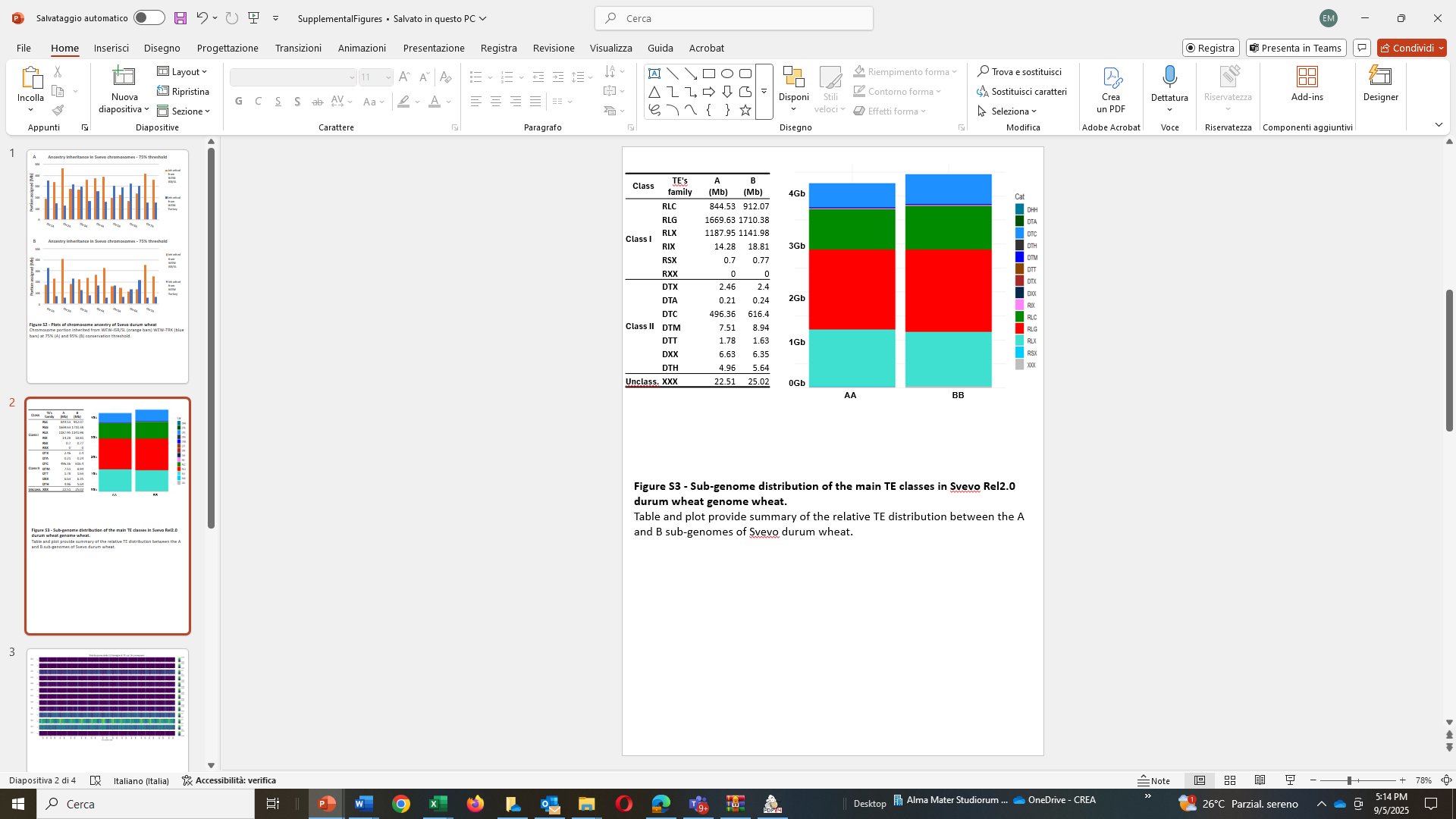


**Figure S5 Heatmap showing the genomic distribution of 13 transposable element (TE) families across chromosomes 1A and 1B of durum wheat (*Triticum turgidum* ssp. *durum*).** TE densities were calculated as the fraction of bases covered within non-overlapping 1 Mb windows. Color intensity reflects the local density of each TE family, with independent color scales adjusted to highlight both abundant (RLC, RLX, RLG; maximum fraction set to 0.6) and less abundant families (all others; maximum fraction set to 0.1). Vertical dashed red lines indicate the approximate position of the centromeres, marked with an asterisk. White vertical separators distinguish adjacent chromosomes.


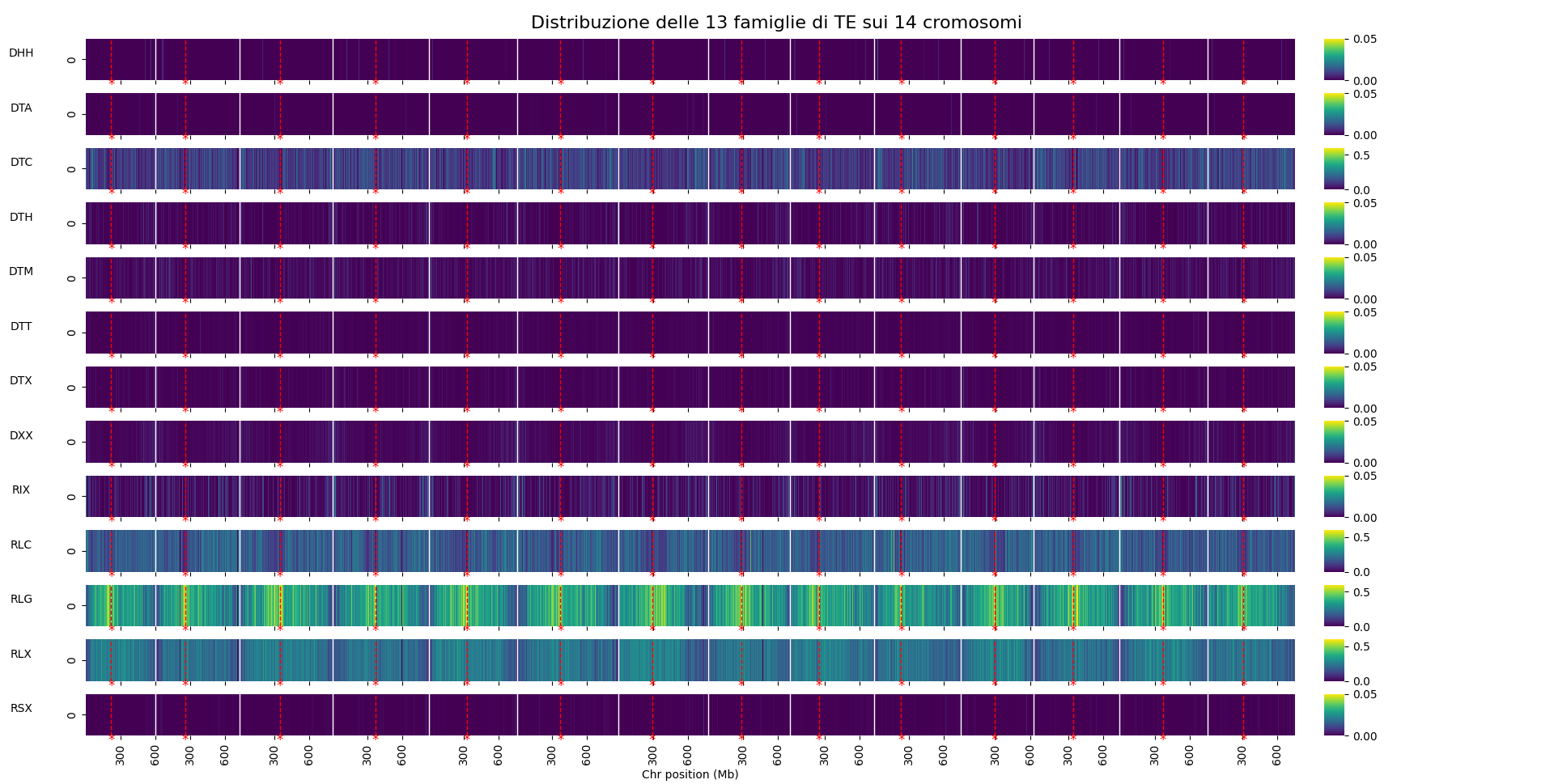


**Figure S6 Sub-genome distribution of annotated genes in Svevo Rel.2.0.** Table (A) and plot (B) provide summary of the annotated gene distribution between the A and B sub-genomes of Svevo durum wheat.

**A**

| **Gene biotype** | **Confidence** | **A (Mb)** | **B (Mb)** |
| --- | --- | --- | --- |
| **Protein coding gene** | HC | 122.15 | 122.82 |
| **Protein coding gene** | LC | 138.01 | 160.25 |
| **Pseudogene** | HC | 0.04 | 0.02 |
| **Transposable element gene** | HC | 3.77 | 3.9 |
| **Transposable element gene** | LC | 62.31 | 71.28 |
| **ncrna gene** | LC | 3.26 | 3.85 |
| **Predicted gene** | LC | 4.79 | 6.1 |

**B**


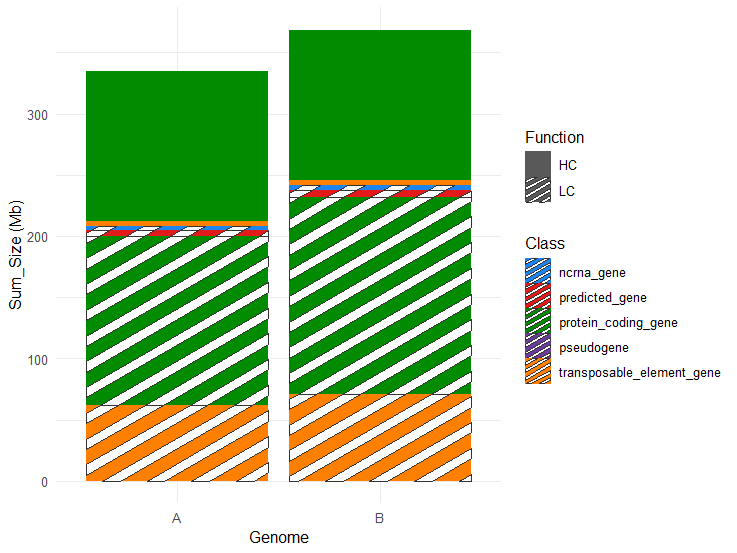


**Figure S7 Comparison of gene models across Rel2.0 and Rel1.0 genome annotations and corresponding gene expression profiles.** Gene structures for selected loci are shown for comparison between Svevo Rel2.0 and Rel1.0 genome annotations in panels A-D. For each locus, exon–intron structures of all annotated transcript isoforms are displayed for the corresponding annotations (left panels). Exons are represented as boxes and introns as connecting lines, with different colors indicating distinct genes within each annotation. Transcript identifiers are shown next to each isoform. Genomic coordinates correspond to the same reference genome assembly. The right panels show the screenshots from the Durum Wheat eFP Browser with the expression profiles for the genes represented in each locus. **A:** TrturSVE3A02G00657080, encoding a dehydration-responsive element-binding (DREB) transcription factor, expressed in the late stages of grain filling and embryo maturation and dehydration. In Rel1.0 this locus was chimeric annotated, fused with the flanking locus. **B:** TrturSVE3B02G00918510, encoding Gibberellin 20-oxidase 2, highly expressed in developing inflorescences primordia and previously annotated as low-confidence gene in Rel1.0. **C:** TrturSVE3B02G00798860–TrturSVE3B02G00798880, encoding class I heat shock proteins, induced by heat-stress in both leaves and roots. The three loci were annotated as single chimeric locus in Rel1.0. **D:** TrturSVE5A02G01397160, corresponding to the VRN2/ZCCT-A2 CCT domain-containing protein locus. This copy of the two tandemly duplicated floral repressors ZCCT1 and ZCCT2 that made the VRN2 locus was not annotated in the Rel1.0 genome.


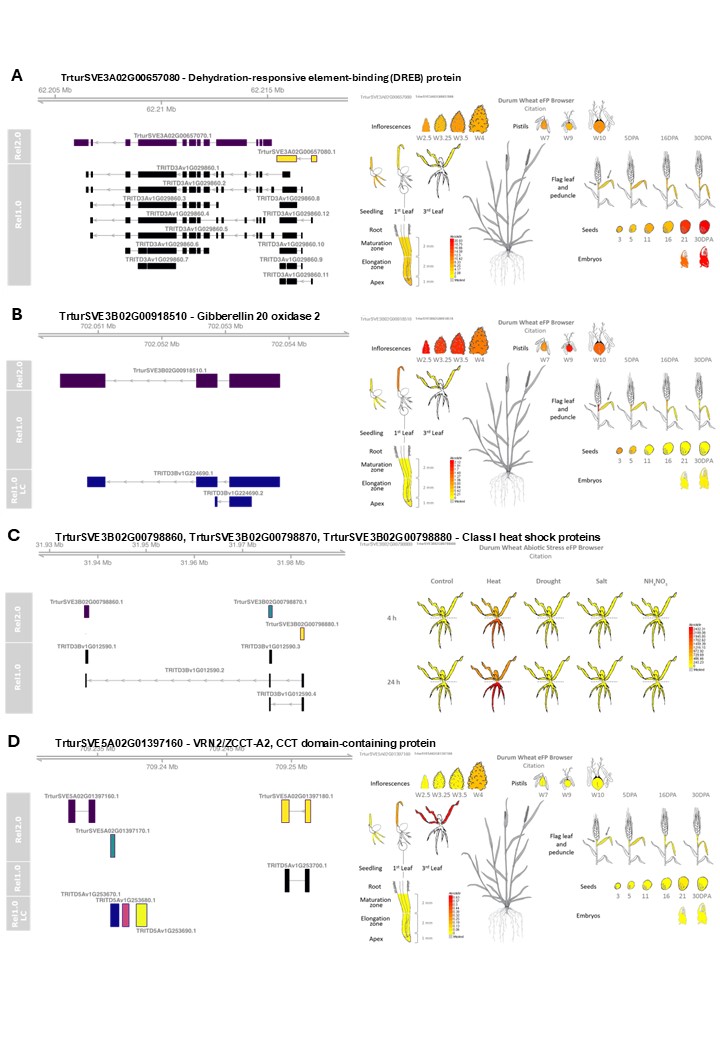


**Figure S8 OMArk analysis (clade pooideae, v0.3) comparing the annotation of Svevo Rel.1.0 and Rel.2.0.** OMArk analysis was performed to compare the completeness and annotation quality between the Rel.1.0 and Rel.2.0 annotations of *Triticum durum* cv. Svevo. The analysis assesses differences in the proportion of missing and unknown genes for all genes, high confidence (HC) plus low confidence (LC) genes or only HC genes. HOGs (Hierarchical Orthologous Groups) represent sets of genes from different species that descend from a single gene in the last common ancestor and serve as the reference framework for assessing annotation quality and completeness.


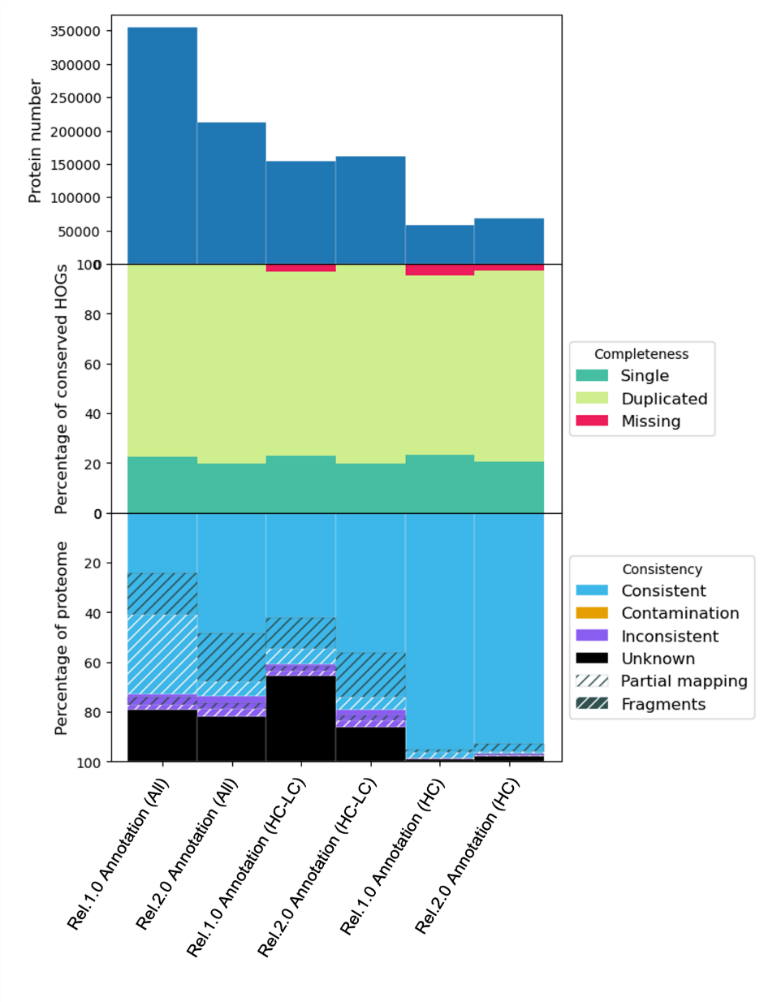


**Figure S9** **Screenshots from the Durum Wheat eFP Browser.** Pictograms showing different wheat developmental stages and tissues included in the development (**A**), nutrient and abiotic stress map (**B**) and abiotic stress view. False color highlights the tissue/organ included in the atlas.


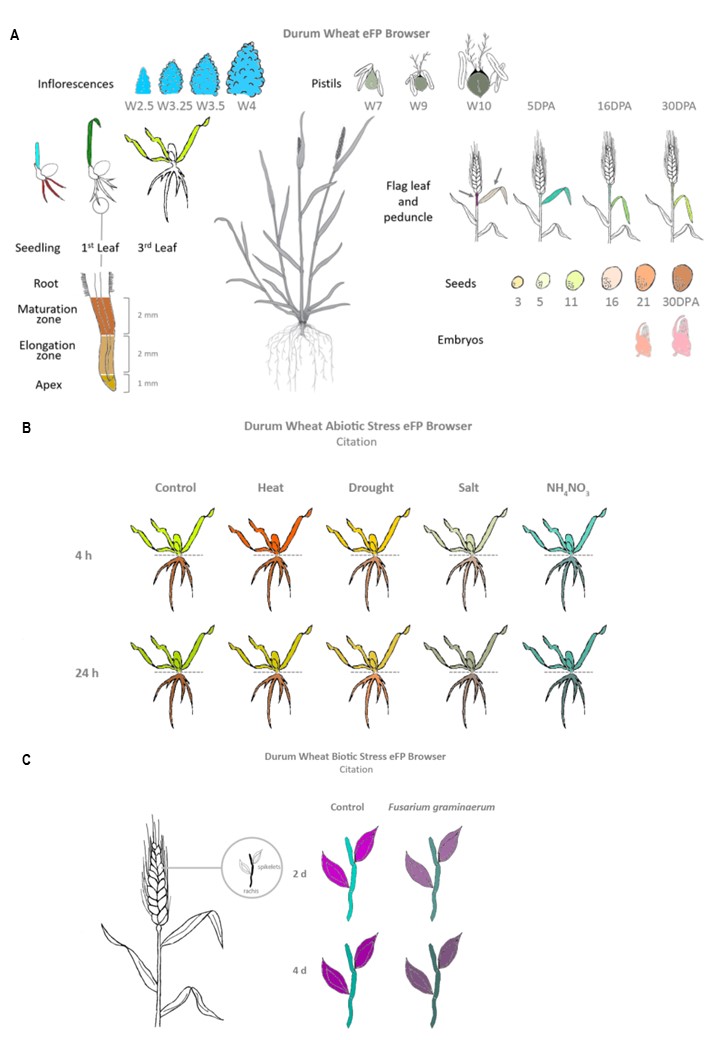


**Figure S10**: Pr

**Figure S10** **Protein sequence alignment of LMW-GS from the Svevo genome.** Cysteine residues predicted to be implicated in intrachain and interchain disulfide bonds are indicated by green and red boxes, respectively.


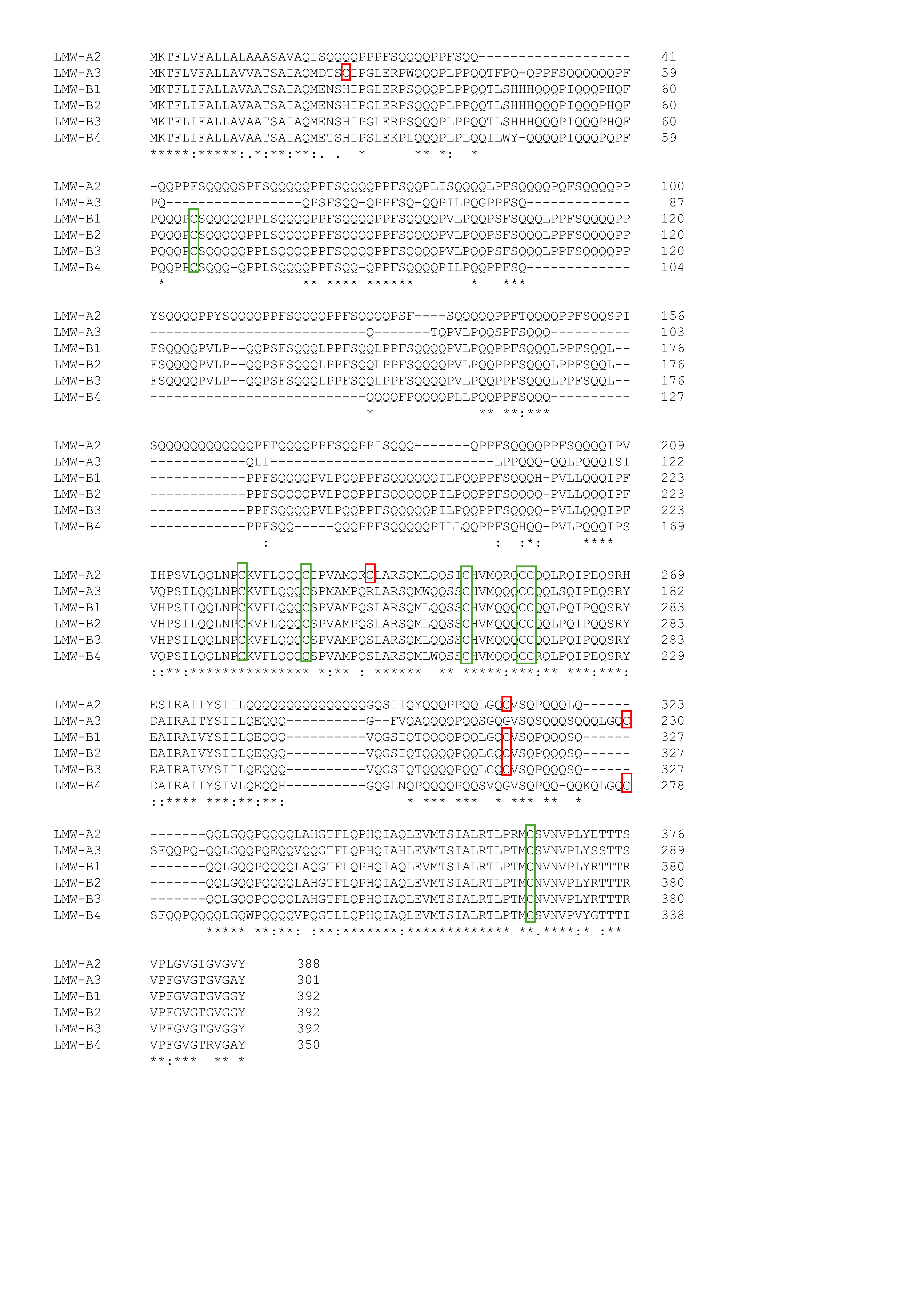


**Figure S11 Schematic representation of the *Tg1-B* locus on the short arm of chr. 2B, with a representation of the Svevo × Zavitan genetic map in the corresponding region.** Genetic markers are aligned with their physical positions on chr. 2B in both the Zavitan WEWSeq v2.1 and the Svevo Rel.2.0 genome assemblies. Break points defining the 5Mb- inversion are indicated with red lines with the physical positions on the two genome assemblies.


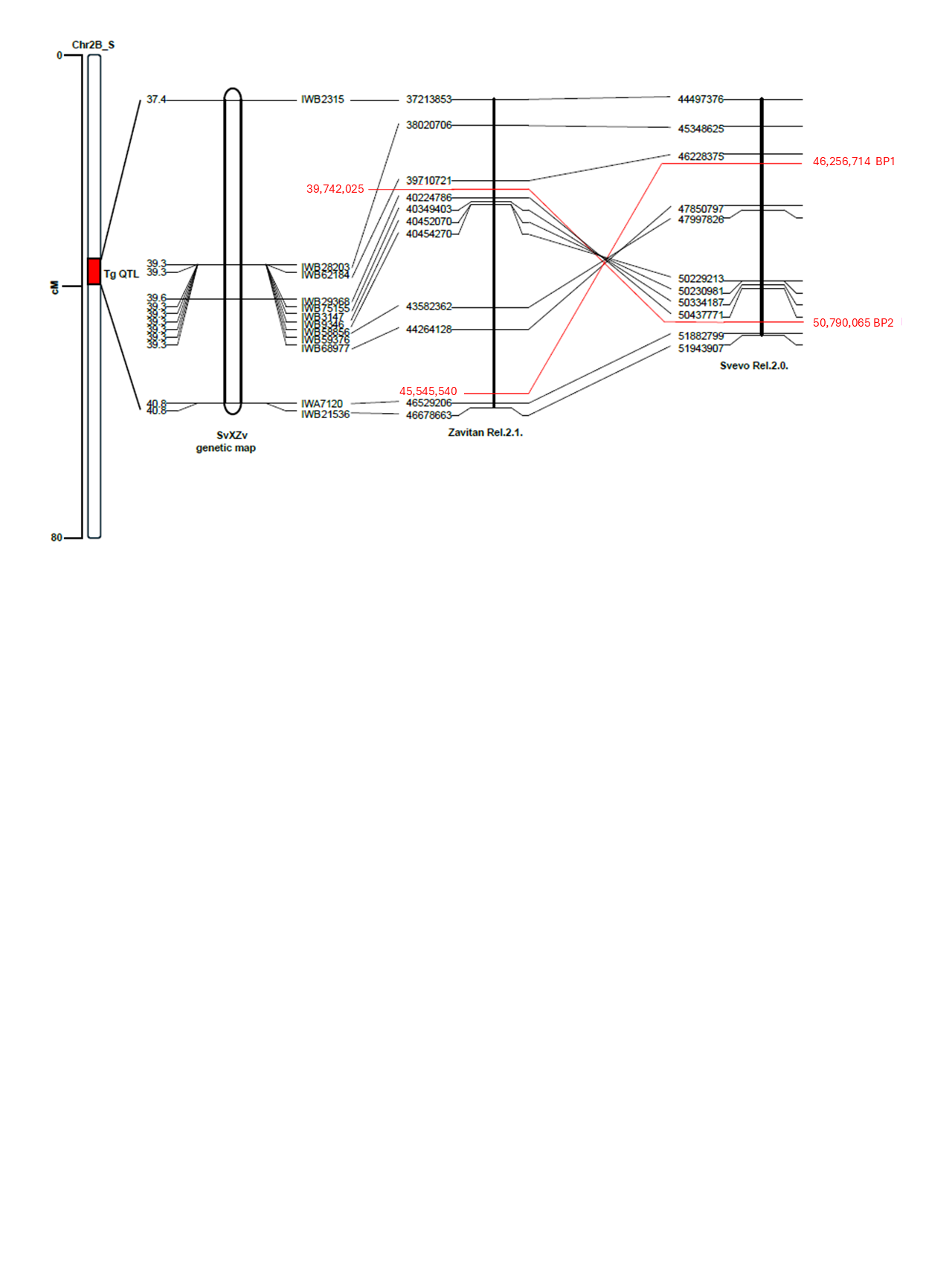


**Figure S12 The QTL hotspot TKW_12. A:** QTL clustered by the hotspot TKW_12 are reported as coloured bars, sized based on the Confidence Interval of each QTL, on the long arm of the chr. 4A. **B:** The eight sucrose:sucrose 1-fructosyltransferase coding genes included in the minimal interval region are listed, together with their position and manual curated annotation. **C:** The gene expression pattern in the different tissues across the plant growth cycle is shown as absolute expression level (TPM).


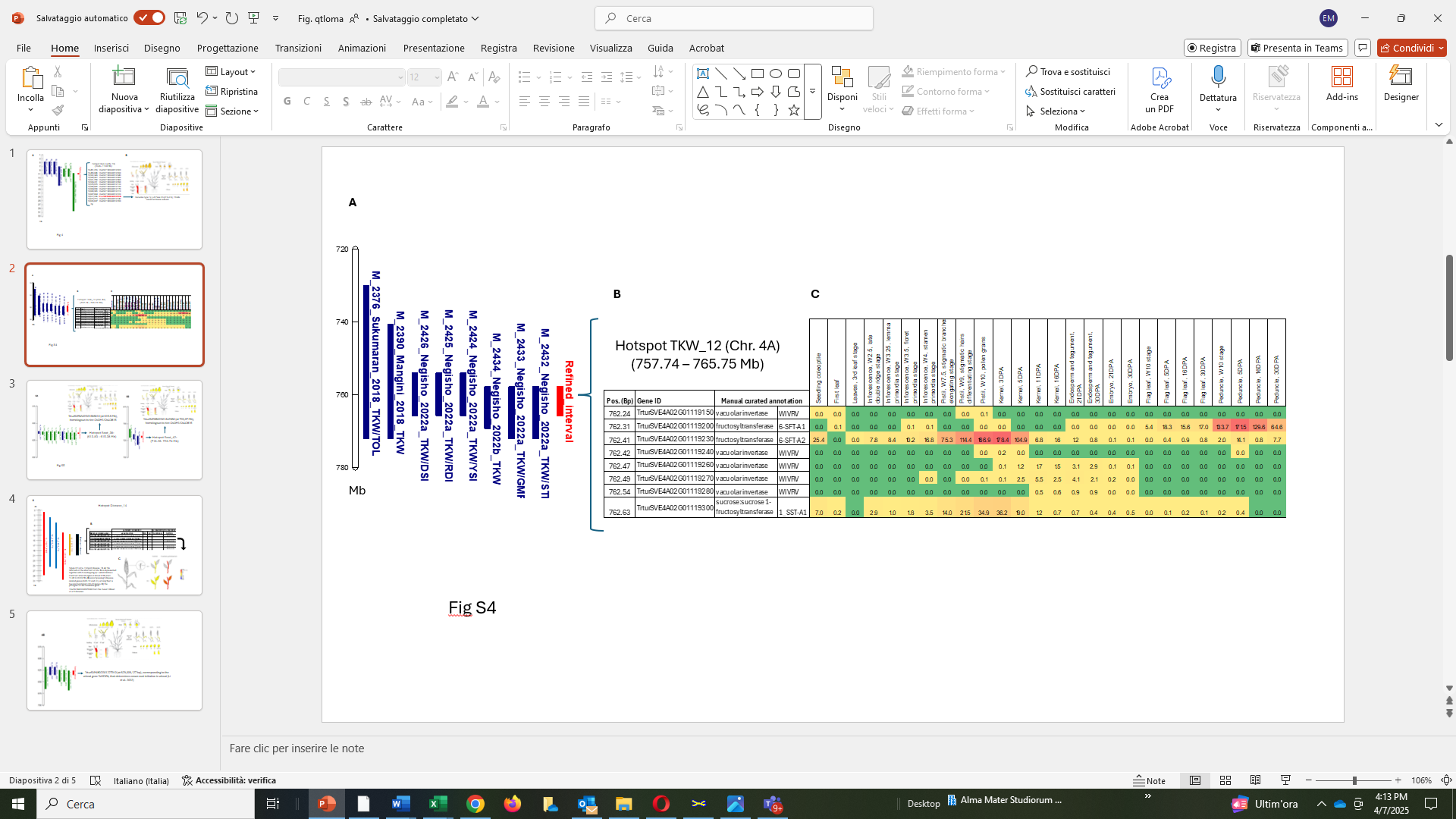


**Figure S13 The QTL hotspot Root_25. A:** QTL clustered by the hotspot Root_25 are reported as coloured bars, sized based on IC range of each QTL, on the long arm of the chr. 4B. **B:** The list of genes encompassed by the minim interval region included *TrturSVE4B02G01227910*, homologus of *TaMORb*, whose expression pattern across tissues of durum wheat genes in represented by the pictogram derived from the Durum Wheat eFP Browser.

**
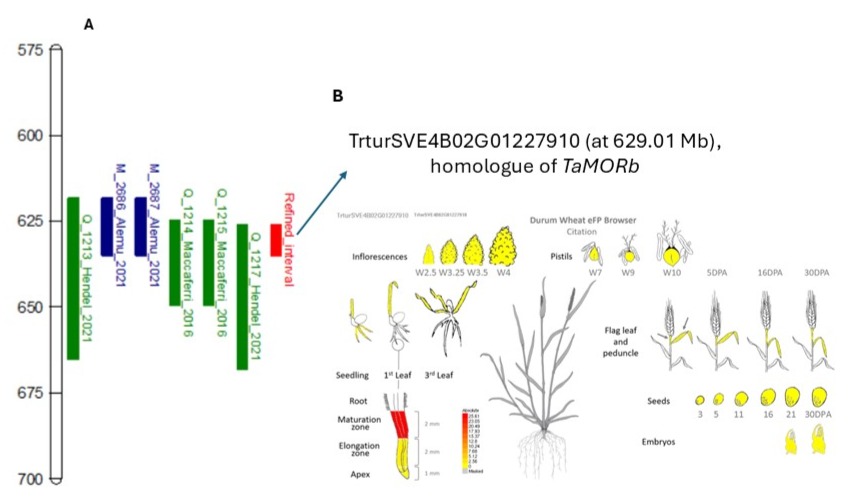
**

**Figure S14 The homeologous QTL hotspots Root_38 and Root_42.** QTL clustered by the hotspot Root_38 and Root_42 are reported as coloured bars (blue for QTL from association mapping and green from linkage mapping), sized based on the Confidence Interval range of each QTL, on segments of the chr. 6A and 6B, respectively in **(A)** and **(C)**. The pictogram, derived from the Durum wheat eFP browser, shows the expression of the homelogous candidate genes (*TrturSVE6A02G01686810* **(B)**, *TrturSVE6B02G01842660* **(D)**) retrieved within the minimum interval region of each hotspot.


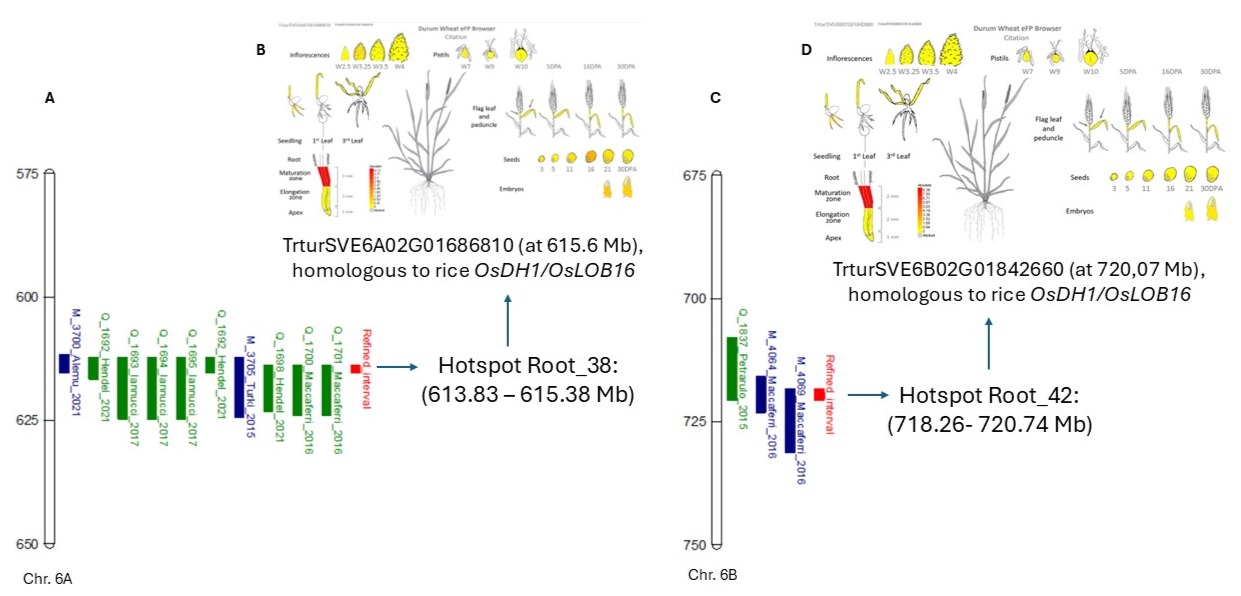


**Figure S15 Svevo haplotype composition.** The Svevo genome was divided into windows comprising 100 SNPs each. The windows were painted based on the percentage of the composition of the SNP-based clustering per window. Blue color indicates origin from wild emmer accessions collected in Turkey and yellow color indicates origin from wild emmer collected in Israel-Southern Levant. Intermediate color tones indicate situations in which it is not possible to determine the origin. Some windows in the Svevo genome originate from the *T. timopheevii* wheat lineage (AAGG gene pool) and were labelled with purple color. The white line indicates the robustness of the assignment of each window based on the number of wild emmer accessions supporting the assignment to a particular subpopulation, while the black line indicates the minimal summed SNP score.


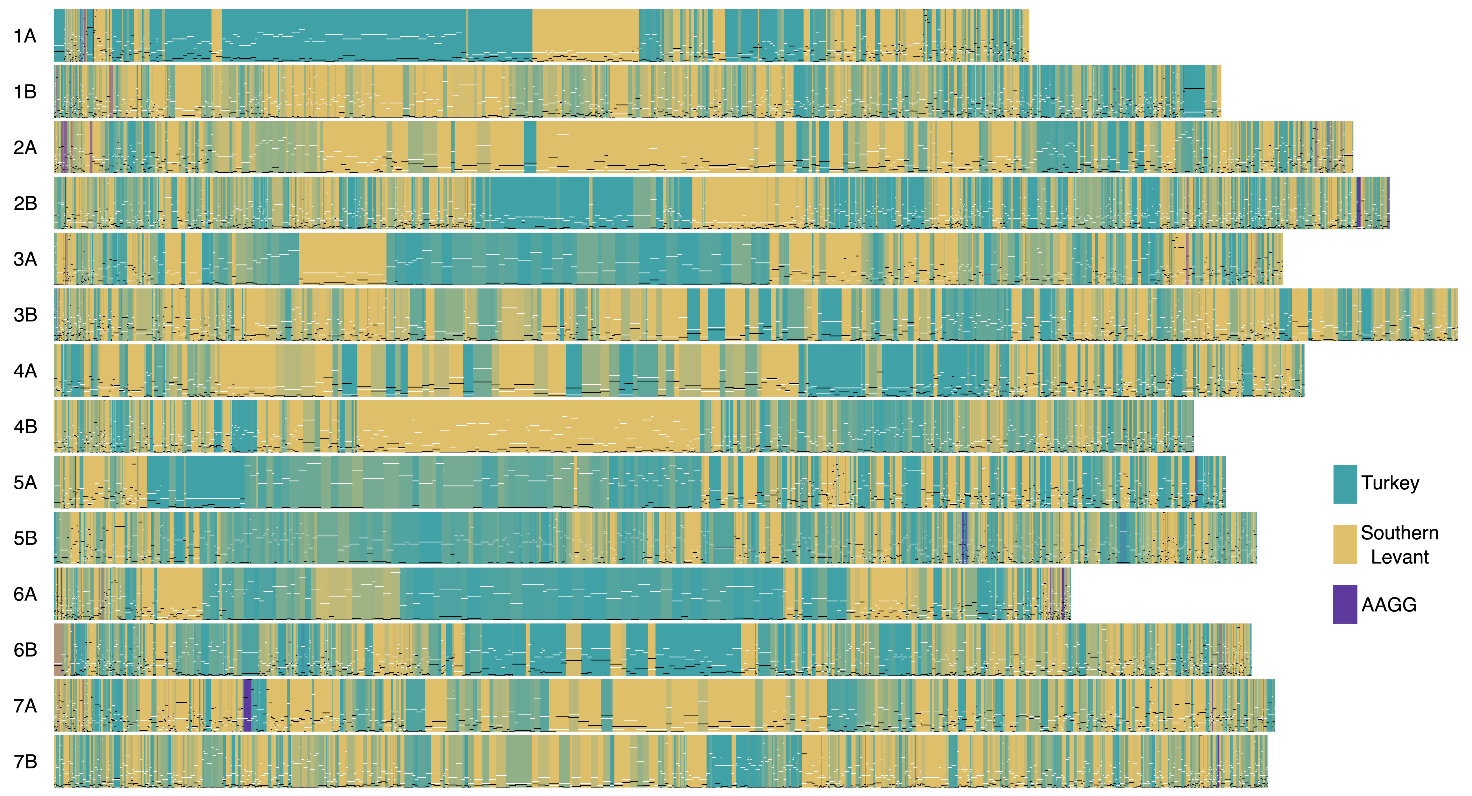


**Figure S16 Alignment of amplicons to display insertions/deletions causing size variations among amplicon obtained by assessing diversity at the locus chr. 2B *Tg1-B* in the 285 diverse panel.** Alignment of amplicon sequence mostly retrieved in hulled accessions (H1, 2, 3), positions are related to Zavitan WEWSeq 2.0 (more detailed in Table S12c).


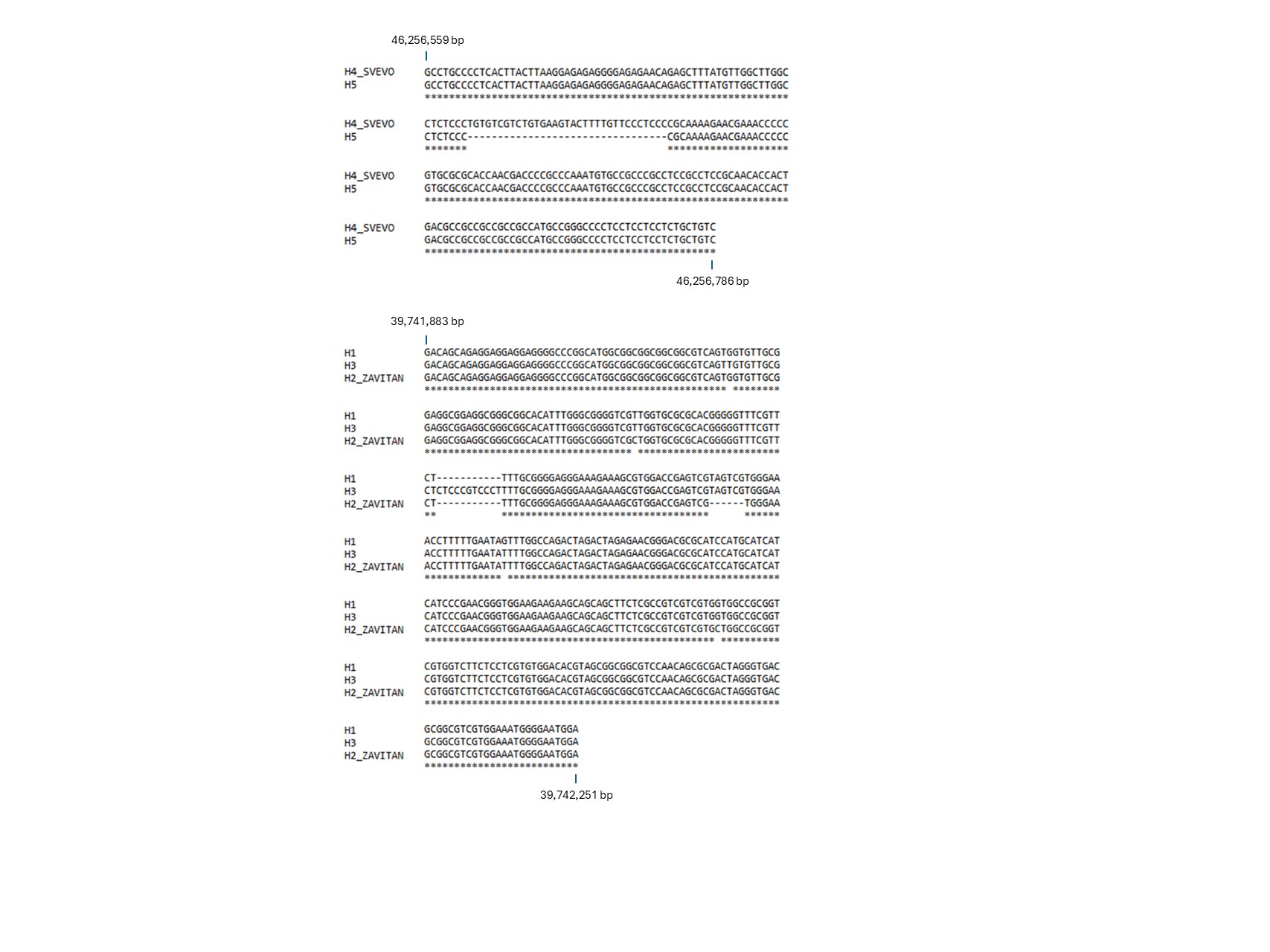


Alignment of amplicon sequence mostly retrieved in free-threshing accessions (H4, H5), positions are related to Svevo Rel.2.0 (more detailed in Table S12c).


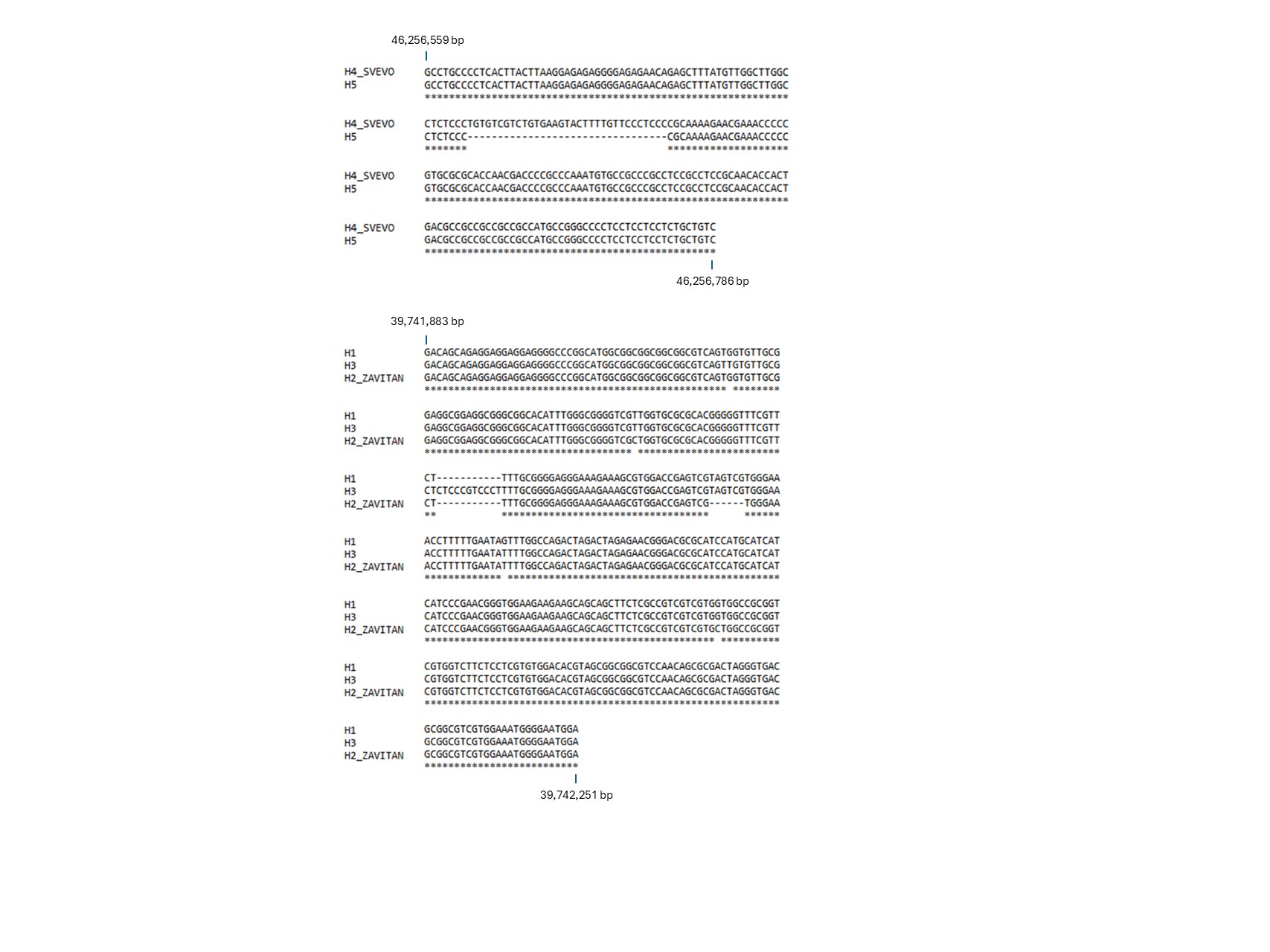


**Figure S17 The QTL hotspot Disesase_14. A)** QTL clustered by the hotspot Disease_14 are reported as coloured bars, sized based on IC range of each QTL, on the long arm of the chr. 4A; **B**) the list of genes encompassed by the minim interval region (both HC and LC) including a few non functional LRR-CR genes; **C**) the pictogram of the candidate gene *TrturSVE4A02G00978380*, derived from the Durum wheat eFP browser.


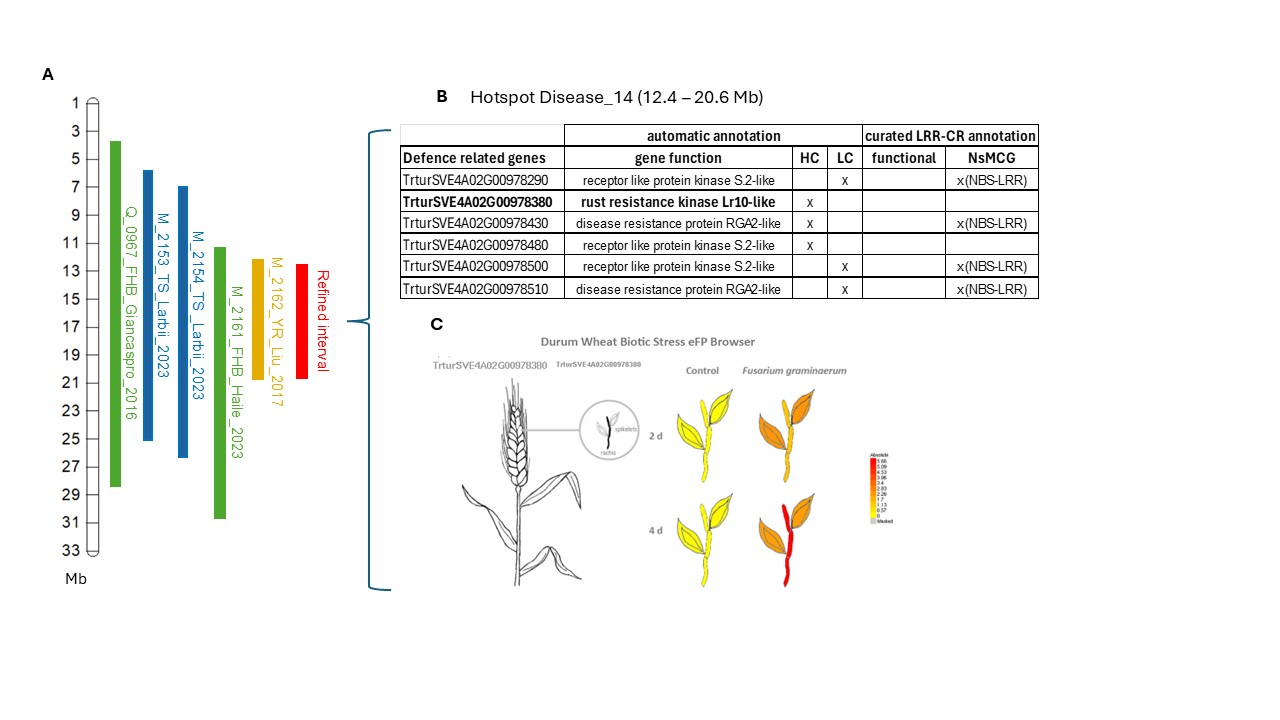


**References**

Abdennur N, Mirny LA (2020) Cooler: scalable storage for Hi-C data and other genomically labeled arrays. *Bioinformatics*. doi: [10.1093/bioinformatics/btz540](https://doi.org/10.1093/bioinformatics/btz540).

Bailey TL, Johnson J, Grant CE, Noble WS (2015) The MEME Suite. *Nucleic Acids Research* 43(W1): W39-W49. doi: [10.1093/nar/gkv416](https://doi.org/10.1093/nar/gkv416)

Beccari G, Prodi A, Pisi A, Nipoti P, Onofri A, Nicholson P, Pfohl K, Karlovsky P, Gardiner DM, Covarelli L (2017) Development of three fusarium crown rot causal agents and systemic translocation of deoxynivalenol following stem base infection of soft wheat. *Plant Pathology* 67: 1055-1065. doi: [10.1111/ppa.12821](https://doi.org/10.1111/ppa.12821)

Bolger AM, Lohse M, Usadel B (2014) Trimmomatic: a flexible trimmer for Illumina sequence data. *Bioinformatics* 30: 2114-20. doi: 10.1093/bioinformatics/btu170.

Bray NL, Pimentel H, Melsted P, Pachter L (2016) Near-optimal probabilistic RNA-seq quantification. *Nature Biotechnology* 34: 525–527. doi: [10.1038/nbt.3519](https://doi.org/10.1038/nbt.3519)

Browning BL, Tian X, Zhou Y, Browning SR (2021) Fast two-stage phasing of large-scale sequence data. *American Journal Humane Genetics* 108: 1880-1890. [doi: 10.1016/j.ajhg.2021.08.005](https://doi.org/10.1016/j.ajhg.2021.08.005)

Castro-Mondragon JA, Riudavets-Puig R, Rauluseviciute I, et al. (2022) JASPAR 2022: the 9th release of the open-access database of transcription factor binding profiles. *Nucleic Acids Research* 50(D1):D165-D173. doi: 10.1093/nar/gkab1113

Chen XY, Cao XY, Zhang YJ, IslamS, Zhang JJ, Yang RC, Liu JJ, Li GY, Appels R, Keeble-Gagnere G, Ji WQ, He ZH, Ma WJ (2016) Genetic characterization of cysteine-rich type-b avenin-like protein coding genes in common wheat. *Scientific Reports* 6: 30692. doi: 10.1038/srep30692

Cheng H, Concepcion GT, Feng X, Zhang H, Li H (2021) Haplotype-resolved de novo assembly using phased assembly graphs with hifiasm. *Nature Methods* 18: 170-175. doi 10.1038/s41592-020-01056-5

Chou HL, Tian L, Fukuda M, Kumamaru T, Okita TW (2019) The role of RNA-binding protein OsTudor-SN in post-transcriptional regulation of seed storage proteins and endosperm development. *Plant Cell Physiology* 60: 2193-2205. doi: 10.1093/pcp/pcz113.

Clavijo BJ, Venturini L, Schudoma C, et al. (2017) An improved assembly and annotation of the allohexaploid wheat genome identifies complete families of agronomic genes and provides genomic evidence for chromosomal translocations. *Genome Research* 27: 885-896. doi: [10.1101/gr.217117.116](https://doi.org/10.1101/gr.217117.116)

Danecek P, Auton A, Abecasis G, Albers CA, Banks E, DePristo MA, Handsaker R, Lunter G, Marth G, Sherry ST, McVean G, Durbin R (2011) The variant call format and VCFtools *Bioinformatics* 27: 2156–2158. doi: [10.1093/bioinformatics/btr330](https://doi.org/10.1093/bioinformatics/btr330)

Danecek P, Bonfield JK, Liddle J, Marshall J, Ohan V, Pollard MO, Whitwham A, Keane T, McCarthy SA, Davies RM, Li H (2021) Twelve years of SAMtools and BCFtools. *GigaScience* 10: giab008. doi: [10.1093/gigascience/giab008](https://doi.org/10.1093/gigascience/giab008)

Darvasi A, Soller M (1997) A simple method to calculate resolving power and confidence interval of QTL map location. *Behavour Genetics* 27:125-32. doi: 10.1023/a:1025685324830.

Dia Sow M, Forestan C, Pont C (2025) Striking convergent selection history of wheat and barley and its potential for breeding. *Nature Plants 11, 2268–2285* , doi: 10.1038/s41477-025-02128-0

Dievart A, Gottin C, Périn C, Ranwez V, Chantret N (2020) Origin and diversity of plant Receptor-Like Kinases. *Annual Review in Plant Biology* 71: 131-156. doi: 10.1146/annurev-arplant-073019-025927

Dobin A, Davis CA, Schlesinger F, Drenkow J, Zaleski C, Jha S, Batut P, Chaisson M, Gingeras TR (2013) STAR: ultrafast universal RNA-seq aligner. *Bioinformatics* 29: 15-21. doi: 10.1093/bioinformatics/bts635

Dreos R, Ambrosini G, Périer R, Bucher P (2014) The Eukaryotic Promoter Database: expansion of EPDnew and new promoter analysis tools. *Nucleic Acids Research* 43(Database issue): D92-96. doi: 10.1093/nar/gku1111; PMID: 25378343.

Duan F, Ding J, Lee D, Lu X, Feng Y, Song W (2017) Overexpression of SoCYP85A1, a spinach Cytochrome p450 gene in transgenic tobacco enhances root development and drought stress tolerance. *Frontiers in Plant Science* 8:1909. doi: 10.3389/fpls.2017.01909

Dudchenko O, Batra SS, Omer AD, Nyquist SK, Hoeger M, Durand NC, Shamim MS, Machol I, Lander ES, Aiden AP, Aiden EL. De novo assembly of the *Aedes aegypti* genome using Hi-C yields chromosome-length scaffolds. *Science*. 2017 Apr 7;356(6333):92-95. doi: 10.1126/science.aal3327.

Feuillet C, Travella S, Stein N, Albar L, Nublat A, Keller B (2003) Map-based isolation of the leaf rust disease resistance gene Lr10 from the hexaploid wheat (*Triticum aestivum* L.) genome. *Proceedings of the National Academy of Sciences USA* 100: 15253-15258. doi: 10.1073/pnas.2435133100

Fornasiero A, Feng T, Al-Bader N, et al. (2024) Oryza genome evolution through a tetraploid lens. *bioRxiv* 596369; doi: 10.1101/2024.05.29.596369

Gain C, François O (2021) LEA 3: Factor models in population genetics and ecological enomics with R. *Molecular Ecology Resources* 21. 2738-2748. doi: [10.1111/1755-0998.13366](https://doi.org/10.1111/1755-0998.13366)

Gotoh O (2008) A space-efficient and accurate method for mapping and aligning cDNA sequences onto genomic sequence. *Nucleic Acids Research* 36*:* 2630–2638. doi: [10.1093/nar/gkn105](https://doi.org/10.1093/nar/gkn105)

Gottin C, Dievart A, Summo M, Droc G, Périn C, Ranwez V, Chantret N (2021) A new comprehensive annotation of leucine-rich repeat-containing receptors in rice. *The Plant Journal* 108: 492-508. doi: 10.1111/tpj.15456

Grant CE, Bailey TL, Stafford Noble W (2011) FIMO: Scanning for occurrences of a given motif. *Bioinformatics* 27: 1017-1018. doi: [10.1093/bioinformatics/btr064](https://doi.org/10.1093/bioinformatics/btr064)

Gu YQ, Coleman-Derr D, Kong X, Anderson OD (2004) Rapid genome evolution revealed by comparative sequence analysis of orthologous regions from four Triticeae genomes. *Plant Physiology* 135: 459–470. doi: 10.1104/pp.103.038083

Haas BJ, Salzberg SL, Zhu W, Pertea M, Allen JE, Orvis J, White O, Buell CR, Wortman JR (2008) Automated eukaryotic gene structure annotation using EVidenceModeler and the Program to Assemble Spliced Alignments. *Genome Biology* 9: R7. <https://doi.org/10.1186/gb-2008-9-1-r7>

Hahne F, Ivanek R (2016) Visualizing genomic data using Gviz and Bioconductor. *Methods Molecular Biology* 1418:335-51. doi: 10.1007/978-1-4939-3578-9_16

Hallab A (2014). Protein Function Prediction using Phylogenomics, Domain Architecture Analysis, Data Integration, and Lexical Scoring. PhD thesis, University of Bonn, http://hdl.handle.net/20.500.11811/ 6420.

Han GZ (2019) Origin and evolution of the plant immune system. *New Phytologist* 222: 70–83. doi: [10.1111/nph.15596](https://doi.org/10.1111/nph.15596)

Hiebert CW, Moscou MJ, Hewitt T et al. (2020) Stem rust resistance in wheat is suppressed by a subunit of the mediator complex. *Nature Communication* 11: 1123. doi: 10.1038/s41467-020-14937-2

Hufford MB, Seetharam AS, Woodhouse MR, et al. (2021) De novo assembly, annotation, and comparative analysis of 26 diverse maize genomes. *Science* 373: 655-662. doi: 10.1126/science.abg5289

Huo N, Dong L, Zhang S, et al. (2017) New insights into structural organization and gene duplication in a 1.75‐Mb genomic region harboring the α‐gliadin gene family in *Aegilops tauschii* , the source of wheat D genome. *The Plant Journal* 92: 571–583. doi: 10.1111/tpj.13675.

Huo N, Zhang S, Zhu T, Dong L, Wang Y, Mohr T, Hu T, Liu Z, Dvorak J, Luo M-C, Wang D, Lee J-Y, Altenbach S, Gu YQ (2018a) Gene duplication and evolution dynamics in the homeologous regions harboring multiple prolamin and resistance gene families in hexaploid wheat. *Frontiers in Plant Science* 9:673. doi: 10.3389/fpls.2018.00673

Huo N, Zhu T, Altenbach S, Dong L, Wang Y, Mohr T, Liu Z, Dvorak J, Luo M-C, Gu YQ (2018b) Dynamic evolution of α-gliadin prolamin gene family in homeologous genomes of hexaploid wheat. *Scientific Reports* 8: 5181. doi: 10.1038/s41598-018-23570-5.

Huo N, Zhu T, Zhang S, Mohr T, Luo M-C, Lee J-Y, Distelfeld A, Altenbach S, Gu YQ (2019) Rapid evolution of α-gliadin gene family revealed by analyzing Gli-2 locus regions of wild emmer wheat. *Functional Integrative Genomics* 19: 993–1005. doi: [10.1007/s10142-019-00686-z](https://doi.org/10.1007/s10142-019-00686-z).

Jones P, Binns D, Chang HY, Fraser M, Li W, McAnulla C, McWilliam H, Maslen J, Mitchell A, Nuka G, Pesseat S, Quinn AF, Sangrador-Vegas A, Scheremetjew M, Yong SY, Lopez R, Hunter S (2014) InterProScan 5: Genome-scale protein function classification. *Bioinformatics* 30, 1236–1240. doi: [10.1093/bioinformatics/btu031](https://doi.org/10.1093/bioinformatics/btu031)

Kang YJ, Yang DC, Kong L, Hou M, Meng YQ, Wei L, Gao G (2017) CPC2: a fast and accurate coding potential calculator based on sequence intrinsic features. *Nucleic Acids Research* 45(W1): W12-W16. doi: 10.1093/nar/gkx428]

Kerpedjiev P, Abdennur N, Lekschas F, McCallum C, Dinkla K, Strobelt H, Luber JM, Ouellette SB, Azhir A, Kumar N, Hwang J, Lee S, Alver BH, Pfister H, Mirny LA, Park PJ, Gehlenborg N (2018) [HiGlass: Web-based visual comparison and exploration of genome interaction maps.](https://genomebiology.biomedcentral.com/articles/10.1186/s13059-018-1486-1) Genome Biology*,* 19:125. doi: 10.1186/s13059-018-1486-1

Kim D, Paggi JM, Park C, Bennett C, Salzberg SL (2019) Graph-based genome alignment and genotyping with HISAT2 and HISAT-genotype. *Nature Biotechnology* 37: 907–915. doi: [10.1038/s41587-019-0201-4](https://doi.org/10.1038/s41587-019-0201-4)

Kolodziej MC, Singla J, Sánchez-Martín J et al. (2021) A membrane-bound ankyrin repeat protein confers race-specific leaf rust disease resistance in wheat. *Nature Communication* 12: 956. doi: 10.1038/s41467-020-20777-x

Kong L, Zhang Y, Ye Z-Q, Liu X-Q, Zhao S-Q, Wei L, Gao G (2007) CPC: assess the protein-coding potential of transcripts using sequence features and support vector machine. *Nucleic Acids Research* 35(Web Server issue): W345–9. doi: 10.1093/nar/gkm391

Kong X-Y, Gu YQ, You FM,Dubcovsky J, Anderson OD (2004) Dynamics of the evolution of orthologous and paralogous portions of a complex locus region in two genomes of allopolyploid wheat. *Plant Molecular Biology* 54: 55–69. doi: 10.1023/B:PLAN.0000028768.21587.dc.

Kovaka S, Zimin AV, Pertea GM, Razaghi R, Salzberg SL, Pertea M (2019) Transcriptome assembly from long-read RNA-seq alignments with StringTie2. *Genome Biology* 20: 1–13. doi: [10.1186/s13059-019-1910-1](https://doi.org/10.1186/s13059-019-1910-1)

Lawrence M, Huber W, Pagès H, Aboyoun P, Carlson M, Gentleman R, Morgan MT, Carey VJ (2013) Software for computing and annotating genomic ranges. *PLoS Computation Biology* 9: e1003118. doi: 10.1371/journal.pcbi.1003118.

Leigh JW, Bryant D (2015) Popart: full-feature software for haplotype network construction. *Methods in Ecology and Evolution* 6: 1110-1116. <https://doi.org/10.1111/2041-210X.12410>

Lev-Mirom Y, Ashkenazy N, Klymiuk V, Enns J, Wiebe K, Schuenemann VJ, Eilam T, Kulikovsky S, Lavi BA, Günther T, Krugman T, Sela H, Klein E, Ganor A, Davidovich U, Porat R, Ben-Yosef E, David M, Frankin S, Ben David R, Cavalet-Giorsa E, Krattinger SG, Korol A, Mascher M, Himmelbach A, Stein N, Krause J, Distelfeld A, Weiss E, Fahima T, Pozniak CJ, Hübner S (2026) Ancient grains illuminate the mosaic origin of domesticated wheat. *Nature Plants*, in press.

Li B, Dewey CN (2011) RSEM: accurate transcript quantification from RNA-seq data with or without a reference genome. *BMC Bioinformatics* 12: 323. doi: 10.1186/1471-2105-12-323.

Li H (2018) Minimap2: pairwise alignment for nucleotide sequences. *Bioinformatics* 34: 3094–3100. doi: [10.1093/bioinformatics/bty191](https://doi.org/10.1093/bioinformatics/bty191)

Li H (2018) Minimap2: pairwise alignment for nucleotide sequences. *Bioinformatics* (Oxford, England) 34: 3094–3100. doi: [10.1093/bioinformatics/bty191](https://doi.org/10.1093/bioinformatics/bty191)

Li H (2023) Protein-to-genome alignment with miniprot. *Bioinformatics* (Oxford, England) 39: btad014. [doi: 10.1093/bioinformatics/btad014](https://doi.org/10.1093/bioinformatics/btad014)

Li H (2013) Aligning sequence reads, clone sequences and assembly contigs with BWA-MEM. [arXiv:1303.3997v2](http://arxiv.org/abs/1303.3997)

Loutre C, Wicker T, Travella S, Galli P, Scofield S, Fahima T, Feuillet C, Keller B (2009) Two different CC-NBS-LRR genes are required for Lr10-mediated leaf rust resistance in tetraploid and hexaploid wheat. *The Plant Journal* 60: 1043-54. doi: 10.1111/j.1365-313X.2009.04024.x.

Love MI, Huber W, Anders S (2014) Moderated estimation of fold change and dispersion for RNA-seq data with DESeq2. *Genome Biology* 15: 550. doi: 10.1186/s13059-014-0550-8.

Maccaferri M, El-Feki W, Nazemi G, Salvi S, Canè MA, Colalongo MC, Stefanelli S, Tuberosa R (2016) Prioritizing quantitative trait loci for root system architecture in tetraploid wheat. *Journal of Experimental Botany* 67: 1161-78. doi: 10.1093/jxb/erw039.

Maccaferri M, Harris NS, Twardziok SO et al. (2019) Durum wheat genome highlights past domestication signatures and future improvement targets. *Nature Genetics* 51**:** 885–895. doi: [10.1038/s41588-019-0381-3](https://doi.org/10.1038/s41588-019-0381-3).

Maccaferri M, Ricci A, Salvi S, et al. (2015) A high-density, SNP-based consensus map of tetraploid wheat as a bridge to integrate durum and bread wheat genomics and breeding. *Plant Biotechnology Journal*: 13: 648-663. doi: 10.1111/pbi.12288.

Mailund T (2019) Manipulating data frames: dplyr. In: R data science quick reference. Apress, Berkeley, CA. <https://doi.org/10.1007/978-1-4842-4894-2_7>

Manni M, Berkeley MR, Seppey M, Simão FA, Zdobnov EM (2021) BUSCO update: novel and streamlined workflows along with broader and deeper phylogenetic coverage for xcoring of eukaryotic, prokaryotic, and viral genomes. *Molecular Biology Evolution* 38: 4647-4654. doi: [10.1093/molbev/msab199](https://doi.org/10.1093/molbev/msab199)

Mapleson D, Venturini L, Kaithakottil G, Swarbreck D (2018) Efficient and accurate detection of splice junctions from RNA-seq with Portcullis. *GigaScience* 7:giy131. doi: 10.1093/gigascience/giy131

Marçais G, Delcher AL, Phillippy AM, Coston R, Salzberg SL, Zimin A. MUMmer4: A fast and versatile genome alignment system. *PLoS Computational Biology* 2018 14: e1005944. doi: 10.1371/journal.pcbi.1005944.

Mazzucotelli E, Sciara G, Mastrangelo AM, Desiderio F, Xu SS, Faris J, Hayden MJ, Tricker PJ, Ozkan H, Echenique V, Steffenson BJ, Knox R, Niane AA, Udupa SM, Longin FCH, Marone D, Petruzzino G, Corneti S, Ormanbekova D, Pozniak C, Roncallo PF, Mather D, Able JA, Amri A, Braun H, Ammar K, Baum M, Cattivelli L, Maccaferri M, Tuberosa R, Bassi FM (2020) The Global Durum Wheat Panel (GDP): An International Platform to Identify and Exchange Beneficial Alleles. *Frontiers in Plant Science* 11:569905. doi: 10.3389/fpls.2020.569905

Mehrabi AA, Pour-Aboughadareh A, Mansouri S et al. (2020) Genome-wide association analysis of root system architecture features and agronomic traits in durum wheat. *Molecular Breeding* 40: 55. doi: [10.1007/s11032-020-01136-6](https://doi.org/10.1007/s11032-020-01136-6)

Minh BQ, Schmidt HA, Chernomor O, Schrempf D, Woodhams MD, von Haeseler A, Lanfear R (2020) IQ-TREE 2: new models and efficient methods for phylogenetic inference in the genomic era, *Molecular Biology and Evolution* 37: 1530–1534. doi: [10.1093/molbev/msaa015](https://doi.org/10.1093/molbev/msaa015)

Navrátilová P, Toegelová H, Tulpová Z, Kuo YT, Stein N, Doležel J, Houben A, Šimková H, Mascher M (2022) Prospects of telomere-to-telomere assembly in barley: Analysis of sequence gaps in the MorexV3 reference genome. *Plant Biotechnology Journal* 20: 1373-1386. doi: 10.1111/pbi.13816

Nevers Y, Warwick Vesztrocy A, Rossier V, Train CM, Altenhoff A, Dessimoz C, Glover NM (2025) Quality assessment of gene repertoire annotations with OMArk. *Nature Biotechnology* 43: 124–133. doi: [10.1038/s41587-024-02147-w](https://doi.org/10.1038/s41587-024-02147-w)

Open2C, Abdennur N, Fudenberg G, Flyamer IM, Galitsyna AA, Goloborodko A, et al. (2024) Pairtools: From sequencing data to chromosome contacts. *PLoS Computational Biology* 20(5): e1012164. https://doi.org/10.1371/journal.pcbi.1012164

Parker MT, Knop K, Barton GJ, Simpson GG (2021) 2passtools: two-pass alignment using machine-learning-filtered splice junctions increases the accuracy of intron detection in long-read RNA sequencing. *Genome Biology* 22: 72. [doi: 10.1186/s13059-021-02296-0](https://doi.org/10.1186/s13059-021-02296-0)

Patro R, Duggal G, Love MI, Irizarry RA, Kingsford C (2017) Salmon provides fast and bias-aware quantification of transcript expression. *Nature Methods* 14(4):417-419. doi: 10.1038/nmeth.4197

Pedregosa F, Varoquaux G, Gramfor A, et al. (2011) Scikit-learn: machine learning in python. *The Journal of Machine Learning Research* 12: 2825 – 2830.

Percio F, Rubio L, Amorim-Silva V, Botella MA (2025) Crucial Roles of Brassinosteroids in cell wall composition and structure across species: new insights and biotechnological applications. *Plant Cell* *Environment* 48: 1751-1767. doi: 10.1111/pce.15258

Pertea G, Pertea M (2020) GFF Utilities: GffRead and GffCompare. *F1000Research* 9: 304 doi: [10.12688/f1000research.23297.1](https://doi.org/10.12688/f1000research.23297.1)

Poplin R, Ruano-Rubio V, DePristo MA, Fennell TJ, Carneiro MO, Van der Auwera GA, Kling DE, Gauthier LD, Levy-Moonshine A, Roazen D, Shakir K, Thibault J, Chandran S, Whelan C, Lek M, Gabriel S, Daly MJ, Neale B, MacArthur DG, Banks E (2017) Scaling accurate genetic variant discovery to tens of thousands of samples.*BioRxiv,* 201178. doi: 10.1101/201178

Quinlan AR, Hall IM (2010) BEDTools: a flexible suite of utilities for comparing genomic features. *Bioinformatics* 26: 841–842. DOI: [10.1093/bioinformatics/btq033](https://doi.org/10.1093/bioinformatics/btq033)

Raj A, Stephens M, Pritchard JK (2014) FastSTRUCTURE: Variational inference of population structure in large SNP data sets. *Genetics* 197: 573–589. doi: 10.1534/genetics.114.164350

Robinson JT, Turner D, Durand NC, Thorvaldsdóttir H, Mesirov JP, Aiden EL (2018) Juicebox.js provides a cloud-based visualization system for Hi-C data. *Cell Systems* 6: 256-258.e1. doi: 10.1016/j.cels.2018.01.001

Roselló M, Royo C, Sanchez-Garcia M, Soriano JM (2019) Genetic dissection of the seminal root system architecture in Mediterranean durum wheat landraces by Genome-Wide Association Study. A*gronomy* 9:364. doi: [10.3390/agronomy9070364](https://doi.org/10.3390/agronomy9070364)

Sansaloni C, Franco J, Santos B et al. (2020) Diversity analysis of 80,000 wheat accessions reveals consequences and opportunities of selection footprints. *Nature Communication* 11: 4572. doi: 10.1038/s41467-020-18404-w

Seppey M, Manni M, Zdobnov EM (2019) BUSCO: assessing genome assembly and annotation completeness. In: Kollmar M (ed) Gene prediction. *Methods in Molecular Biology*, vol 1962. Humana, New York, NY. [doi.org/10.1007/978-1-4939-9173-0_14](https://doi.org/10.1007/978-1-4939-9173-0_14)

Shao M, Kingsford C (2017) Accurate assembly of transcripts through phase-preserving graph decomposition. *Nature Biotechnology* 35: 1167–1169. [doi: 10.1038/nbt.4020](https://doi.org/10.1038/nbt.4020)

Shumate A, Salzberg SL (2021) Liftoff: accurate mapping of gene annotations. *Bioinformatics* (Oxford, England) 37: 1639–1643. [doi: 10.1093/bioinformatics/btaa1016](https://doi.org/10.1093/bioinformatics/btaa1016)

Spannagl M, Nussbaumer T, Bader KC, Martis MM, Seidel M, Kugler KG, Gundlach H, Mayer KF. (2017) PGSB PlantsDB: updates to the database framework for comparative plant genome research. Nucleic Acids Res. 2016:44

Stanke M, Morgenstern B (2005) AUGUSTUS: a web server for gene prediction in eukaryotes that allows user-defined constraints. *Nucleic Acids Research* 33(Web Server): W465–W467. [doi: 10.1093/nar/gki458](https://doi.org/10.1093/nar/gki458)

Sun Y, Ning T, Liu Z et al. (2015) The *OsSec18* complex interacts with P0(P1-P2)2 to regulate vacuolar morphology in rice endosperm cell. *BMC Plant Biology* 15: 55. doi: 10.1186/s12870-014-0324-1

Thorvaldsdóttir H, Robinson JT, Mesirov JP (2013) Integrative Genomics Viewer (IGV): high-performance genomics data visualization and exploration. *Brief in Bioinformatics* 14(2):178-92. doi: 10.1093/bib/bbs017

Tian H, Fu J, Drijber RA, Gao Y (2015) Expression patterns of five genes involved in nitrogen metabolism in two winter wheat (*Triticum aestivum* L.) genotypes with high and low nitrogen utilization efficiencies. *Journal of Cereal Science* 61: 48-54

Toufighi K, Brady SM, Austin R, Ly E, Provart NJ (2005) The Botany Array Resource: e-Northerns, Expression Angling, and promoter analyses. *The Plant Journal* 43: 153-63. doi: 10.1111/j.1365-313X.2005.02437.x.

UniProt Consortium (2015) UniProt: a hub for protein information. *Nucleic Acids Research* 43(Database issue): D204-12. doi: 10.1093/nar/gku989

Upadhyaya NM, Mago R, Panwar V et al. (2021) Genomics accelerated isolation of a new stem rust avirulence gene–wheat resistance gene pair. *Nature Plants* 7: 1220–1228. doi: 10.1038/s41477-021-00971-5.

Venturini L, Caim S, Kaithakottil GG, Mapleson DL, Swarbreck D (2018) Leveraging multiple transcriptome assembly methods for improved gene structure annotation. *GigaScience*, 7: giy093. doi: 10.1093/gigascience/giy093

Walkowiak S, Gao L, Monat C, et al. (2020) Multiple wheat genomes reveal global variation in modern breeding. *Nature* 588, 277–283. doi: 10.1038/s41586-020-2961-x

Wang, G, Yin H, Li B, et al. (2019) Characterization and identification of long non-coding RNAs based on feature relationship. *Bioinformatics* 35: 2949-2956. doi: 10.1093/bioinformatics/btz008. doi:10.1093/bioinformatics/btz008

Wickham H (2016) ggplot2: Elegant graphics for data analysis. Edited by Springer.

Wu TD, Watanabe CK (2005) GMAP: a genomic mapping and alignment program for mRNA and EST sequences. *Bioinformatics* 21:1859-75. doi: 10.1093/bioinformatics/bti310

Yang L, Chen Y, Xu L, Wang J, Qi H, Guo J, Zhang L, Shen J, Wang H, Zhang F, Xie L, Zhu W, Lü P, Qian Q, Yu H, Song S. (2022) The *OsFTIP6*-*OsHB22*-*OsMYBR57* module regulates drought response in rice. *Molecular Plant* 15: 1227-1242. doi: 10.1016/j.molp.2022.06.003.

Zeng X, Mishina K, Jia J, Distelfeld A, Maughan PJ, Kikuchi S, Sassa H and Komatsuda T (2020) The Brittle Rachis trait in species belonging to the Triticeae and its controlling genes *Btr1* and *Btr2*. *Frontiers in Plant Science* 11:1000. doi: 10.3389/fpls.2020.01000

Zhang W, Chen S, Abate Z, Nirmala J, Rouse MN, Dubcovsky J (2017) Identification and characterization of *Sr13*, a tetraploid wheat gene that confers resistance to the Ug99 stem rust race group, *Proceedings of the National Academy of Sciences USA* 114: E9483-E9492. doi: 10.1073/pnas.1706277114

Zhang Y, Hu X, Islam S, et al (2018) New insights into the evolution of wheat avenin-like proteins in wild emmer wheat (*Triticum dicoccoides*). *Proceedings of the National Academy of Sciences USA* 115: 13312–13317. doi.org/10.1073/pnas.1812855115.

Zhou Y, Thomas Bui, Lisa D. Auckland & Claire G. Williams (2002) Direct fluorescent primers are superior to M13-tailed primers for *Pinus taeda* microsatellites. *BioTechniques* 32: 1, 46-52, doi: 10.2144/02321bm05

Zhu T, Wang L, Rimbert H, Rodriguez JC, Deal KR, De Oliveira R, Choulet F, Keeble-Gagnère G, Tibbits J, Rogers J, Eversole K, Appels R, Gu YQ, Mascher M, Dvorak J, Luo MC (2021) Optical maps refine the bread wheat *Triticum aestivum* cv. Chinese Spring genome assembly. *The Plant Journal* 107: 303-314. doi: 10.1111/tpj.15289.

Zhu T, Wang L, Rodriguez JC, Deal KR, Avni R, Distelfeld A, McGuire PE, Dvorak J, Luo MC (2019) Improved genome sequence of wild emmer wheat Zavitan with the aid of Optical Maps. *G3* (Bethesda) 9: 619-624. doi: 10.1534/g3.118.200902.
