# Supplementary material for: Durum Wheat cv. Svevo Reference Genome Rel.2.0: A Comprehensive Tool for Wheat Genomics
Source: Plant Biotechnol J. 2026 Jul 13:10.1111/pbi.70673. Online ahead of print. doi: 10.1111/pbi.70673 (PMC13398682; doi:10.1111/pbi.70673)
Supplement: Supplementary file 32 — Data S1–S9: pbi70673‐sup‐0032‐Supinfo.zip. [file PBI-9999-0-s020.zip › Dataset 1_CpGI_Svevo.zip!/CpGI_Svevo/CpG-island prediction README.pdf]

Svevo Platinum genome assembly CpG-island prediction

Svevo Platinum assembly CpG-island prediction was carried out with CpGcluster (https://bmcbioinformatics.biomedcentral.com/articles/10.1186/1471-2105-7-446, https://github.com/bioinfoUGR/cpgcluster) with the following parameters: d=50, P-value=1E-5.

1,804,712 putative CpG-islands were detected along all chromosomes and contigs (Table 1).

The **Durum-wheat-Svevo.assembly_v4_CpGcluster.txt** file contains the prediction results. Each line corresponds to a CpG-island prediction. Columns values are as follows:

Chr	Chromosome
CGI	CpG-island nr
From	Start position
To	End position
Length	CpG-island length
Count	Nr of CpGs in island
OEratio	CpG O/E ratio: ratio between observed and expected CpG frequencies
%G+C	Molecular fraction of guanine and cytosine
PatDen	Pattern density
PValue	PValue
logPValue	logPValue

Table 1: CpG-island nr in all chromosomes and contigs.

Chr/contig	Length	Nr of CpGs in sequence	Islands nr
Chr1A	601409218	24344800	93992
Chr1B	720129471	30378717	144685
Chr2A	801978772	32739222	125293
Chr2B	823820094	33941603	126100
Chr3A	757581553	30780977	120644
Chr3B	865721599	35630936	134489
Chr4A	771205369	31340335	123194
Chr4B	703076932	29577505	152032
Chr5A	723247106	29645768	126995
Chr5B	741435680	30992882	137130
Chr6A	627959976	26077765	138164
Chr6B	737505161	30907570	139625
Chr7A	753738911	30491065	125625
Chr7B	748382270	30602066	113837
Contigs (01-45)	19777986	773568	2907
Total	10396970098	428224779	**1804712**
