# Supplementary material for: Durum Wheat cv. Svevo Reference Genome Rel.2.0: A Comprehensive Tool for Wheat Genomics
Source: Plant Biotechnol J. 2026 Jul 13:10.1111/pbi.70673. Online ahead of print. doi: 10.1111/pbi.70673 (PMC13398682; doi:10.1111/pbi.70673)
Supplement: Supplementary file 32 — Data S1–S9: pbi70673‐sup‐0032‐Supinfo.zip. [file PBI-9999-0-s020.zip › Dataset 7_LRRPROFILER_RAW_OUTPUT.zip!/04_LRRPROFILER_RAW_OUTPUT/Svevo_Rel1_HC/LRR_structure_Rel1_HC.html]

LRR\_structure


# LRR\_structure
